# Evaluating methods for testing publication bias in meta-analyses with confounding covariates: A simulation study

**DOI:** 10.3758/s13428-026-03206-7

**Published:** 2026-10-21

**Authors:** Man Chen

**Affiliations:** Department of Educational Psychology, The University of Texas at Austin, 1912 Speedway, Austin, TX 78712 USA

**Keywords:** Meta-analysis, Small-study effects, Publication bias, Between-study heterogeneity

## Abstract

Selective publication of favorable results can bias meta-analytic estimates and threaten the validity of meta-analyses. Many methods that detect and adjust for publication bias perform poorly under substantial between-study heterogeneity. Heterogeneity complicates publication bias assessment because study characteristics may be associated with both effect size estimates and standard errors. These characteristics can induce an effect-precision association that regression-based diagnostics may misattribute to selective publication when such characteristics are omitted. However, it remains unclear how best to incorporate these confounding characteristics across methods and how their performance varies with confounding level, selective publication, residual heterogeneity, and the number of studies. To address this gap, I conduct an extensive Monte Carlo simulation evaluating Egger-type regression tests, residual-based tests, and a likelihood-ratio test of the three-parameter selection model (3PSM) under the one-step *p* value selection mechanism. Results show that when confounding covariates were ignored, Egger-type regression tests and residual analyses exhibited inflated Type I error rates, whereas the 3PSM likelihood-ratio test maintained near-nominal, albeit slightly inflated, Type I error rates across conditions. When confounding covariates were incorporated, Egger-type tests had well-calibrated Type I error rates, residual analyses based on residualizing effect sizes alone tended to be conservative, and the 3PSM test showed slight inflation. Nevertheless, 3PSM consistently yielded higher power than other methods. Overall, the findings suggest that Egger-type regression tests and 3PSM can be used as complementary analyses for assessing selective publication when confounding covariates are present.

**Supplementary Information:**

The online version contains supplementary material available at https://doi.org/10.3758/s13428-026-03206-7.

## Introduction

Meta-analysis is a statistical method that has been widely used for synthesizing results from multiple studies on the same or closely related topics. One goal of meta-analysis is to provide a summary estimate of the effect size of an intervention or the association between constructs. Another important aim of meta-analysis is to examine the variation in the underlying effects across studies, which is referred to as the between-study heterogeneity. Literature reviews show that between-study heterogeneity is prevalent in education, psychology, and many other disciplines (Van Erp et al., 2017; Williams et al., 2022; Wu et al., 2026).

In the presence of between-study heterogeneity, meta-regression provides a way to explore study characteristics that are associated with intervention effects. The overall variability in effect sizes across studies can thus be partitioned into two parts in meta-regression models: *explainable heterogeneity* and *residual heterogeneity* (Peters et al., 2010). Explainable heterogeneity is the variability in effect sizes that can be explained by study characteristics (e.g., study design) when they are included as predictors (or moderators) in meta-regression. Residual heterogeneity, on the other hand, represents the remaining unexplained variability in results across studies. Residual heterogeneity can arise from sampling error or omitted study characteristics that are unmodeled or unmeasured.

The validity of meta-analysis can be greatly threatened by selective publication (Rothstein et al., 2005). Selective publication occurs when studies with favorable results (e.g., statistically significant and positive) are more likely to be reported and included in meta-analysis. Selective publication leads to over-representation of positive findings in literature, biased model estimates in meta-analysis, and incorrect statistical inferences about intervention effects (Rothstein et al., 2005). The bias caused by selective publication is often referred to as publication bias.

Various statistical techniques have been developed to assess and correct for publication bias in meta-analysis. These methods include the visual tool of funnel plot (Light & Pillemer, 1984), diagnostic tests such as the rank order correlation test (Begg & Mazumdar, 1994) and the Egger’s regression test (Egger et al., 1997), non-parametric detection and adjustment methods such as trim-and-fill (Duval & Tweedie, 2000b, a), regression-based methods such as PET/PEESE (Stanley & Doucouliagos, 2014), and selection modeling approach based on statistical significance (Vevea & Hedges, 1995; Vevea & Woods, 2005). However, researchers have found that most of these methods perform inadequately in the presence of high between-study heterogeneity (Carter et al., 2019; Hong & Reed, 2021; McShane et al., 2016; Moreno et al., 2009). For instance, simulation studies found that trim-and-fill and PET/PEESE methods exhibited inflated Type I error rates and reduced power in the presence of between-study heterogeneity.

Between-study heterogeneity poses challenges for assessing publication bias for several reasons. First, the existing diagnostic tests for small-study effects (e.g., Egger’s regression) may produce spurious conclusions about potential publication bias when high between-study heterogeneity presents (Lin, 2020; Sterne et al., 2011). Egger’s regression and its variants are used to detect selective publication by testing the association between the effect size estimate and its precision (e.g., standard error). The pattern that small studies tend to report larger effects indicates small-study effects, potentially caused by selective publication. However, funnel plot asymmetry or small-study effects can arise from many sources other than selective publication. One such cause is between-study heterogeneity that might be explained by methodological factors (e.g., methodological quality) or clinical factors (e.g., intervention intensity). For example, small studies might report exaggerated intervention effects due to poor quality, or they might show large effects because of careful implementation of the intervention (Sterne et al., 2005). If these study characteristics are ignored, either because they are unmeasured or unmodeled, the diagnostic tests can have inflated Type I error rates and lead to incorrect inferences about the existence of potential selective publication (Reed, 2023a, b).

Second, it is unclear how to account for confounding covariates that are correlated with the effect size estimate and its precision in the investigation of publication bias. One way to incorporate these covariates is to include them as predictors in Egger’s meta-regression models while allowing for residual heterogeneity (Peters et al., 2010; Stanley & Doucouliagos, 2014). The test for the association between the effect size and precision is expected to be more reliable after accounting for the potential confounding effects of observed covariates. Another approach is to use residual regression analyses after removing the impact of covariates on effect size estimates (Nakagawa et al., 2022). That is, one can calculate the residuals of a random-effects meta-regression model that includes relevant covariates, plot residuals against effect size standard errors, and test for their association. Another class of methods is the selection model, which incorporates covariates into the effect-size model. Although these methods can incorporate observed confounders, it is unclear how they perform under different conditions. More importantly, it is impossible to identify and report all sources of heterogeneity (Sterne et al., 2011). It remains a question of how unmeasured or unmodeled confounders affect the assessment of publication bias.

Third, there is a lack of methodological evaluation of methods that address publication bias in the presence of confounding. Existing simulation studies have examined the performance of various detection and adjustment methods for publication bias, assuming heterogeneity is purely unexplained or random (Carter et al., 2019; Hong & Reed, 2021; McShane et al., 2016; Rodgers & Pustejovsky, 2021). Peters and colleagues discussed several approaches, including two regression tests, trim-and-fill, and *p* value selection models, for identifying and adjusting publication bias in meta-analysis with both explainable and residual heterogeneity (Peters et al., 2010). They used two empirical meta-analysis examples to illustrate the assessment of publication bias without conducting simulations to evaluate the performance of different methods. In addition, they only considered a few methods while more techniques are currently available. To the best of my knowledge, no methodological work has been conducted to compare statistical methods for assessing publication bias in meta-analysis with confounding covariates. A comprehensive methodological investigation of different methods is particularly necessary to determine how ignoring or partially ignoring confounding might affect methods that rely on different assumptions about the selective publication process.

The purpose of this paper is twofold. First, I review available methods for assessing publication bias in meta-regression models involving explainable and residual between-study heterogeneity, highlighting their assumptions and limitations. I focus on methods that incorporate observed confounding covariates while modeling residual heterogeneity as a random effect. Second, I conduct a Monte Carlo simulation study to evaluate the Type I error rates and statistical power of several publication bias tests, including the Egger-type regression tests, residual analyses, and a likelihood-ratio test based on the three-parameter selection model (3PSM), under the one-step *p* value selection mechanism. I focus on the one-step selection mechanism because it is prevalent in practice (e.g., Banks et al., 2016; Pigott et al., 2013) and has been widely used in simulation studies (e.g., Carter et al., 2019; McShane et al., 2016; Pustejovsky & Rodgers, 2019). I consider heterogeneity due to two confounding covariates: a binary and a continuous covariate. Specifically, I investigate the consequences of completely ignoring or partially ignoring the explainable heterogeneity in these methods when testing for potential selective publication. The study aims to understand under what conditions and through what mechanisms omitting covariates might undermine the detection of publication bias. I also examine the scenarios where confounding is fully incorporated for a comprehensive comparison across different approaches.

This paper is organized as follows. In the next two sections, I introduce the meta-regression model and review available statistical methods for identifying publication bias when there exist both explainable and residual heterogeneity. The next section presents the simulation study, including the data-generating process, analytical methods, simulation design, and performance measures. I discuss the simulation findings in the following simulation results section. I close with a discussion of the findings, limitations, and further research directions, and implications for applied meta-analyses.

## Methods for assessing publication bias in meta-regression

### Meta-regression model

In the univariate meta-analysis where each study reports a single effect size estimate, a fixed-effect (i.e., common-effect) or random-effects meta-analytic model can be used to synthesize effect sizes across studies. In a fixed-effect model, all effect size estimates are assumed to be measures of a single underlying effect size parameter, and the observed differences among effect size estimates are purely random. The pooled effect is calculated by the weighted average of individual study effects. The most commonly used weight for the fixed-effect model is the inverse sampling variance of the effect size estimate.

In a random-effects model, however, we assume effect size estimates are drawn from an underlying distribution of true effects. The effect sizes from different studies are assumed to be normally distributed with an average effect parameter and some between-study heterogeneity. An optimal weight used in a random-effects model is the inverse of the sum of between-study heterogeneity variance and sampling variance. In order to explain the variation in effect sizes across studies, one can add study-level characteristics as covariates in a random-effects model. This leads to a random-effects meta-regression model, or more broadly, a mixed-effects model. Because it is almost impossible to observe or model all covariates, there often remains residual heterogeneity that is not explained by the included covariates.

Random-effects meta-regression models can account for residual heterogeneity in two ways (Thompson & Sharp, 1999). One way is to employ a multiplicative factor to the sampling variance of the effect size estimate. Another way is to incorporate an additive between-study heterogeneity component. I adopt the additive heterogeneity approach because it is recommended and most often used in meta-analyses (Pellegrini et al., 2025; Thompson & Sharp, 1999).

Consider a meta-analysis with k studies. Let $Y_i$ denote the effect size estimate from study i for i=1,⋯,k with known sampling variance $s_i^2$. A random-effects meta-regression model with two predictors has the form

1 $$\begin{aligned} Y_i = \beta _0 + \beta _1 X_{1i} + \beta _2 X_{2i} + u_i + e_i, \end{aligned}$$

where $\beta _0$ is the intercept and $\beta _1$ and $\beta _2$ are slopes for $X_1$ and $X_2$, respectively. $u_i$ is the study-level random effect with $u_i \sim N(0, \tau ^2)$ and $e_i$ is the sampling error with $e_i \sim N(0, s_i^2)$. In this meta-regression, the residual between-study heterogeneity, $\tau ^2$, represents the remaining effect size heterogeneity after a certain amount of variation is predicted by the set of covariates.

### Egger’s regression tests

A funnel plot provides a visual inspection tool for investigating small-study effects and potential selective publication. It is a scatter plot of the effect size estimate against some measure of its precision (Light & Pillemer, 1984). When the effect size estimate is plotted on the horizontal axis and precision (e.g., standard error) on the vertical axis, the funnel plot has the shape of an inverted funnel with more variability among effects from smaller studies than those from larger studies. In the absence of selective publication, effect size estimates are distributed around the underlying effect parameter, showing a symmetric funnel plot. However, if small studies tend to report larger effect sizes, the funnel plot tends to be asymmetric, potentially caused by selective publication or between-study heterogeneity.

Egger’s regression test (Egger et al., 1997) and its variants have been widely used to test for funnel plot asymmetry or small-study effects. One approach to specify Egger’s regression test is to use a meta-regression model where the effect size estimate is regressed against its standard error with the inverse sampling variance weight. A random-effects meta-regression model with additive residual heterogeneity is given by

2 $$\begin{aligned} Y_i = \beta _0 + \beta _1 s_i + u_i + e_i, \end{aligned}$$

where the slope $\beta _1$ captures the association between the effect size estimate and standard error. A key assumption in Egger’s regression for testing small-study effects and potential selective publication is that no confounding covariates might cause the relationship between the effect estimate and standard error. However, this assumption may be violated when some study characteristics (e.g., study quality) influence both the magnitude of effect size estimate and its standard error. Ignoring such confounding effects can result in a spurious association between effect size estimates and standard errors, leading to incorrect tests for funnel plot asymmetry, small-study effects, and potential publication bias.

Several methods have been proposed to control for the confounding effects of covariates in the examination of small-study effects or selective publication bias while incorporating between-study heterogeneity in meta-analysis. One method extended Egger’s regression by including explanatory covariates or moderators as control variables and using a multiplicative factor for the residual heterogeneity (Stanley & Doucouliagos, 2014) or an additive residual heterogeneity term (Nakagawa et al., 2022). Another method is to compute residuals of effect sizes after controlling for covariates and assess the association between effect size residuals and precision measures (Nakagawa et al., 2022). Both the extended Egger’s regression and residual regression method test for small-study effects, which are potentially caused by selective publication. The selection model, on the other hand, provides a more direct way to model the underlying selection mechanism and can incorporate covariates and residual heterogeneity in the effect size model.

#### Egger-SE

Sterne and Egger (2005) extended Egger’s regression to a meta-regression model with additive residual heterogeneity. The model with two covariates has the form

3 $$\begin{aligned} Y_{i} = \beta _0 + \beta _1 s_i + \beta _2 X_{1i} + \beta _3 X_{2i} + u_i + e_i, \end{aligned}$$

where the slope $\beta _1$ represents the relationship between effect size and standard error after accounting for covariates, $X_1$ and $X_2$, that might be associated with both effect sizes and standard errors. The random effect, $u_i \sim N(0, \tau ^2)$, is the additive residual heterogeneity term and $e_i \sim (0, s_i^2)$ is the sampling error with known sampling variance $s_i^2$. I call this model *Egger-SE* because it includes the standard error as the predictor.

#### Egger-V

Although standard error has been widely used in funnel plots and Egger-type regression tests, other predictors have also been considered, including sampling variance, primary-study sample size, and the inverse of these quantities (Moreno et al., 2009; Sterne & Egger, 2001). I call this model *Egger-V*, which is specified with

4 $$\begin{aligned} Y_{i} = \beta _0 + \beta _1 s_i^2 + \beta _2 X_{1i} + \beta _3 X_{2i} + u_i + e_i, \end{aligned}$$

where the slope $\beta _1$ indicates the association between effect size and sampling variance after incorporating potential confounding covariates for the association. The PEESE method models a quadratic relationship between the effect size estimate and standard error to account for the possibility that small-study effects might be stronger for effect sizes from studies with higher standard errors and lower power.

Note that the *Egger-SE* and *Egger-V* are equivalent to PET (i.e., precision-effect test) and PEESE (i.e., precision-effect estimator with standard error) in a meta-regression model with additive heterogeneity, respectively (Stanley, 2017; Stanley & Doucouliagos, 2014). I use the terms *Egger-SE* and *Egger-V* to highlight that these regression-based tests are modifications of Egger’s regression test.

In Egger-SE and Egger-V, the slope $\beta _1$ captures the association between the effect size estimate and its standard error or sampling variance. If effect sizes are coded so that larger values indicate more favorable outcomes, then a positive slope indicates that smaller studies with larger standard errors tend to report larger effects, a pattern consistent with small-study effects. In contrast, a negative slope implies that smaller studies with higher uncertainty are less likely to report larger effects, which does not suggest small-study effects or selective publication. Thus, we can assess small-study effects by conducting a one-tailed hypothesis test for $H_0: \beta _1 \le 0$.

The use of Egger-type meta-regression models for assessing small-study effects or publication bias is contingent on the assumption that the association between the effect size estimate and standard error or sampling variance is not confounded by other factors. However, the sampling variance of the standardized mean difference effect size is correlated with the effect size estimate (Pustejovsky & Rodgers, 2019). Following the suggestion in Pustejovsky and Rodgers (2019), I remove the artificial correlation between the effect size estimate and sampling variance when using Egger-SE and Egger-V methods. Moreover, the Egger-type regression tests can be compromised by covariates that are correlated with both the effect estimate and the predictor. If all covariates are included as control variables, the test for the slope is expected to have a rejection rate close to the nominal level in the absence of selective publication. However, if some important covariates are omitted, the detection of small-study effects would be exaggerated, leading to inflated Type I error rates in testing small-study effects.

### Residual-based tests

An alternative method for examining small-study effects while controlling for confounding covariates is the residual analysis approach (Nakagawa et al., 2022). In the univariate meta-analysis where each study reports one effect size, a residual regression involves two steps. In the first step, a random-effects model is fit by regressing effect sizes on a set of covariates. The residuals of the fitted model are calculated by subtracting the predicted values of effect sizes from the observed effects. That is,

5 $$\begin{aligned} R_i = Y_i - \hat{Y}_i = Y_i - (\hat{\beta }_0 + \hat{\beta }_1 X_{1i} + \hat{\beta }_2 X_{2i}), \end{aligned}$$

where $R_i$ is the marginal residual for the effect size estimate in study i, after accounting for the relationship between effect size estimates and covariates.^1^

In the second step, the residuals calculated in the first step are regressed on standard errors:

6 $$\begin{aligned} R_i = \gamma _0 + \gamma _1 s_i + \nu _i + e_i, \end{aligned}$$

where $\gamma _1$ is the slope coefficient of the standard error, $\nu _i$ is the between-study random effect, and $e_i$ is the within-study random error. To be consistent with the Egger-type regression tests, I use the modified standard error, removing the artificial correlation between the effect size estimate and its standard error. $\gamma _1$ can be used to test for the association between effect size residuals and modified standard errors. An alternative model might include the sampling variance as a predictor to explore the nonlinear association between effect size residuals and sampling variances.

Residualizing effect sizes alone is limited because it assumes that the sampling standard error (or sampling variance) is independent of covariates (Nakagawa et al., 2022). If covariates are correlated with standard errors, the slope estimate will underestimate the relationship between effect size estimates and standard errors (Freckleton, 2002), making it less likely to reject the null hypothesis. To obtain an unbiased estimate of the relationship between effect size estimates and standard errors, we can regress the effect-size residuals on the standard-error residuals using the same set of covariates (Frisch & Waugh, 1933; Lovell, 1963). That is, in the second step, the residual of the effect size is regressed on the residual of the standard error using

7 $$\begin{aligned} R_i = \gamma _0 + \gamma _1 R_{s,i} + \nu _i + e_i, \end{aligned}$$

where $R_{s,i}$ is the residual from regressing the standard error $s_{i}$ on the confounding covariates $X_{1i}$ and $X_{2i}$. $\gamma _1$ is the slope coefficient representing the association between effect size residuals and standard error residuals. This extended two-step residual regression method is equivalent to Egger-SE as they produce the same point estimate of the association between effect size estimates and standard errors.^2^ Note that the standard error of the slope coefficient in the residual analysis can be slightly different from the one in Egger-SE due to the small discrepancies in the between-study heterogeneity estimate. Nevertheless, the residual analyses offer no clear practical advantage over Egger-type regression tests and are less straightforward to apply.

### Three-parameter selection model

Another class of statistical methods for dealing with selective publication is selection models that assume certain selection mechanisms by which studies are reported or censored. The selection model technique works by incorporating a specific selection model into the estimation of the meta-analytic parameters, removing the distortions caused by the selection process. One commonly used selection model is *p* value-based selection model, where the probability of observing a study depends on the statistical significance of the result. One conventional form of *p* value selection model is the three-parameter selection model (3PSM, Hedges, 1992; Vevea & Hedges, 1995).

The 3PSM entails two components: a random-effects meta-regression model for the effect size distribution in the absence of selection and a selection function based on the p value of the result. The random-effects meta-regression model includes the fixed-effects parameters ($\boldsymbol{\beta }$) and the (residual) between-study heterogeneity variance $\tau ^2$ (see Eq. 1 for details). The original selection function in 3PSM is a step function with a single parameter λ that quantifies the relative probability of observing a non-significant result versus a significant result. It is assumed that a study is always reported if the effect size estimate is in the favorable direction and statistically significant at a certain step or α level (e.g., p≤.025). A study is reported with a probability λ<1 if the effect size estimate is not favorable in the expected direction or it is not significant. Based on the effect size distribution defined in the random-effects meta-regression model and the one-step selection function, maximum likelihood estimation can be used to estimate the model parameters in a 3PSM model after accounting for the underlying form of selective publication.

A likelihood-ratio test can be used to test for the presence of selective publication in the 3PSM (Rodgers & Pustejovsky, 2021; Vevea & Hedges, 1995). The likelihood-ratio test compares two meta-analytic models, one assumes a certain level of selective publication ($\lambda \ge 0$) and another assumes no selection λ=1. A significant test statistic suggests the potential selective reporting of significant or positive results in the expected direction (Vevea & Hedges, 1995). Note that a one-tailed 3PSM likelihood-ratio test is preferred because it is unlikely that censoring occurs in favor of results that are non-significant or not in the expected direction (Rodgers & Pustejovsky, 2021).

## Simulation methods

I conducted a Monte Carlo simulation study to evaluate the performance of existing methods for detecting publication bias in meta-analyses involving both explainable and residual between-study heterogeneity. I assumed a random-effects meta-regression model with one binary and one continuous covariate, although the simulation also applies for more generalized meta-regression models. I adopted the one-step *p* value selective publication mechanism because it is the conventional form of selection commonly assumed in simulation studies (Carter et al., 2019; Pustejovsky & Rodgers, 2019). The simulation study was designed to answer two questions:

1. What are the effects of ignoring the covariates that are correlated with the effect size estimate and its precision when testing for publication bias in meta-analysis?
2. How do available methods for detecting selective publication perform across varying conditions while controlling for covariates correlated with the effect size estimate and precision?

### Data generation process

I simulated independent meta-analytic datasets with standardized mean difference as the effect size measure. I generated the full meta-analytic data using the random-effects meta-regression model with one binary covariate and one continuous covariate in several steps. First, I sampled primary study sample size, N, from an empirical distribution (Carter et al., 2019) and generated two covariates that were negatively correlated with the primary study sample size N. Note that a negative correlation between covariates and primary study sample size indicates a positive correlation between covariates and the sampling variance or standard error of the effect size estimate. For generating the continuous covariate $X_2$, I standardized the sampled primary study sample size using $Z_N = \frac{N - \bar{N}}{S_N}$, where $\bar{N}$ and ${S_N}$ are the mean and standard deviation of the sample size drawn from the empirical distribution. Given the correlation between the continuous covariate and primary study sample size, $\text {Corr}(X_2, N) = \rho _2$, I computed a continuous variable $X_2 = \rho _2 \times Z_N + \sqrt{1-\rho _2^2} \times E$, where E is a normalized random error that is independent of $Z_N$. For generating the binary covariate, I first generated a latent continuous variable $X_1^*$ following the same process described above for generating the continuous variable. I then dichotomized the generated latent continuous variable to obtain a binary variable $X_1$. I selected the cutoff value of the latent variable so that the resulting correlation between the binary variable and primary study sample size was as close as possible to the target correlation parameter $\text {Corr}(X_1, N) = \rho _1$.

After simulating covariates, I generated the effect size parameter $\delta _i$ from each primary study i for i=1,⋯,k based on the simulated covariates and specified meta-regression fixed and random-effects components. Specifically, I generated the study-specific standardized mean difference effect sizes based on $\delta _i \sim N(\beta _0 + \beta _1 X_1 + \beta _2 X_2, \tau ^2)$, where $\beta _0, \beta _1, \beta _2$ are the intercept, slope parameter of the binary variable, and slope parameter of the continuous covariate, respectively; and $\tau ^2$ is the residual heterogeneity variance.

I then generated the individual participant-level data for each study based on the study-specific effect size parameter $\delta _i$ and the sample size $N_i$. I simulated raw data for a two-group experiment, assuming equal sample sizes. I calculated the small-sample adjusted Hedges’ g effect size estimate and its sampling variance for each primary study. I obtained the one-sided *p* value for the independent-samples t test of each study. I also calculated the modified sampling variance and standard error of each effect size estimate by removing the correlation between the effect size estimate and its sampling variance or standard error.

After simulating meta-analytic data sets, I applied the one-step *p* value selection mechanism where significant and positive effect sizes were more likely to be selected and reported. That is, effect estimates with one-sided p values less than .025 were always selected, while the non-significant or negative effects with one-sided p value greater than .025 were assumed to be reported with a probability (i.e., $\lambda \le 1$). In order to simulate a meta-analysis with exactly k primary studies, I repeated the data-generation process until k primary studies were produced.

### Estimation methods

I programmed the simulations in R (R Core Team, 2024) using the high-throughput computing cluster at the University of Wisconsin - Madison (Center for High Throughput Computing, 2006). I used the metafor (Viechtbauer, 2010) R package for running meta-analytic models. I calculated the modified standard error and sampling variance of the effect size after removing the correlation between the effect estimate and sampling variance (Pustejovsky & Rodgers, 2019). I used the modified standard error and sampling variance when fitting meta-analytic models for all the estimation methods. I investigated the consequences of ignoring both covariates in Egger-SE, Egger-V, and the 3PSM likelihood-ratio test. I then conducted the Egger-SE, Egger-V, residual analyses, and 3PSM test when the binary moderator or both covariates were considered.

The Egger-SE, Egger-V, and residual regression methods were applied using the metafor package, and the 3PSM likelihood-ratio test was calculated using the custom R code developed by Rodgers and Pustejovsky to handle non-convergence issues (Rodgers & Pustejovsky, 2021). The Knapp-Hartung adjustment was used to obtain reliable standard errors and test statistics (Hartung & Knapp, 2001; Knapp & Hartung, 2003) in Egger-SE and Egger-V. The residual analyses were conducted by first calculating residuals from additive random-effects models and then regressing the effect size residual on the modified SE residual or the sampling variance residual. For Egger-SE, Egger-V, and the residual analyses, the between-study heterogeneity was estimated with the restricted maximum likelihood estimator (REML, Viechtbauer, 2005) and the DerSimonian–Laird estimator (DL, DerSimonian & Laird, 1986) if REML does not converge. I used one-sided t-tests to examine the significance of the slopes of the modified precision in Egger-type tests and residual analyses for detecting small-study effects. The 3PSM likelihood-ratio test was calculated for the additive random-effects effect size models. I used a one-sided likelihood-ratio test to assess the significance of selective publication.

### Simulation design

The simulation varied eight factors related to meta-analytic structure and selective publication, including the number of primary studies (k) and residual between-study heterogeneity ($\tau ^2$); the intercept ($\beta _0$) of the meta-regression model and slope parameters for the binary and continuous covariates ($\beta _1$ and $\beta _2$) that define the relations between covariates and the true effects; the correlations between covariates and primary study sample size ($\rho _1$ for the binary covariate and $\rho _2$ for the continuous covariate) that represent the confounding structure; and the degree of censoring (λ). These factors were chosen to evaluate how tests for small-study effects and potential publication bias behave when covariates are associated with true effects, and hence with observed effect size estimates, as well as with study precision, across meta-analyses varying in the number of studies, residual heterogeneity, and degree of censoring. Table 1 summarizes the simulation design. I used a full factorial design with a total of $3 \times 3 \times 1 \times 3 \times 3 \times 6 \times 6 \times 6 = 17,496$ simulation conditions. Each condition was examined via 5000 replications. The R code for replicating the simulation is available on the Open Science Framework at https://osf.io/cjx75.

Each simulated meta-analysis data set included 20, 50, or 80 primary studies, reflecting from small to relatively large meta-analysis sizes. These values were commonly used in meta-analytic simulation studies (Lee & Beretvas, 2023; Pustejovsky & Rodgers, 2019; Rodgers & Pustejovsky, 2021). These numbers were also within the range of the number of studies reported in empirical research syntheses and meta-analyses in psychology, education, and medicine (Tipton et al., 2019).

**Table 1:** Simulation conditions

Simulation parameters	Values
Number of studies (*k*)	20, 50, 80
Between-study residual heterogeneity (τ)	0, 0.2, 0.5
Intercept ($\beta _0$)	0.1
Slope for the binary covariate ($\beta _1$)	0.1, 0.2, 0.5
Slope for the continuous covariate ($\beta _2$)	0.1, 0.2, 0.5
Correlation between binary covariate and N ($\rho _1$)	0.4, 0.2, 0, -0.2, -0.4, -0.6
Correlation between continuous covariate and N ($\rho _2$)	0.4, 0.2, 0, -0.2, -0.4, -0.6
Probability of censoring (λ)	1, 0.8, 0.6, 0.4, 0.2, 0.05

The residual heterogeneity was τ=0,0.2,0.5, corresponding to pure random heterogeneity, small residual heterogeneity, residual heterogeneity, and high residual heterogeneity. These values were similar to those used in a previous simulation study (Lee & Beretvas, 2023) and were selected based on two empirical reviews of psychology meta-analyses. In a review of 705 meta-analyses published in *Psychological Bulletin* from 1990 to 2013, Van Erp and colleagues found that the between-study heterogeneity of standardized mean differences reported in random-effects models ranged from 0 to 0.98 with a mean of 0.16 and a standard deviation of 0.15 (Van Erp et al., 2017). In another review of meta-analyses in cognitive, organizational, and social psychology, Linden and Hönekopp found that the average residual heterogeneity after incorporating moderators in 22 meta-analyses ranged from 0.03 to 1.11 with a mean of 0.36 and a standard deviation of 0.22 (Linden & Hönekopp, 2021).

The intercept was fixed at $\beta _0 = 0.10$, consistent with the value used in a prior simulation study (Lee & Beretvas, 2023). The primary simulation conditions focused on cases in which the covariates were associated with the true effects with $\{\beta _1, \beta _2\} = \{0.1, 0.2, 0.5\}$. The slope parameter values of the binary covariate ($\beta _1$) align with those used by Tipton (2015) to represent small, medium, and large effects of the binary moderators. The slope parameter values of the continuous covariate ($\beta _2$) were chosen to be the same as the binary covariate, making it possible to evaluate the consequences of ignoring covariates with varying moderator effects. To examine the consequences of model misspecification due to including covariates not related to true effects, I conducted supplementary simulations by setting one or both slope parameters to zero. Detailed results of two tests under these supplementary conditions are provided in Supplementary Section S4.

Given that the covariates were generated to be positively related to the true effects, the consequences of omitting them in the tests for selective publication would be affected by the direction of the associations between covariates and standard errors. Thus, the correlations between the covariates and the primary study sample size were varied as $\{\rho _1, \rho _2\} = \{0.4, 0.2, 0, -0.2, -0.4, -0.6\}$. Because standard errors are inversely related to sample sizes, negative correlations between covariates and sample sizes correspond to positive associations between covariates and standard errors. In contrast, positive correlations between covariates and sample sizes represent negative associations between covariates and standard errors. Together, these values allowed me to evaluate confounding effects across a wide range of associations between covariates and study precision.

Pustejovsky and Rodgers (2019) conducted a simulation study to examine the performance of various tests for selective publication in meta-analysis without confounding covariates. They sampled the primary study sample size from the ego depletion effects meta-analysis with total sample sizes ranging from 20 to 251 (Carter et al., 2015). They found that Egger’s regression test and 3PSM likelihood-ratio test using the modified standard errors maintained Type I error rates close to the nominal when sampling within-study sample sizes from the original ego depletion effects dataset. Although the study sample size distribution was right-skewed, with a median of 48 and an interquartile range of 31 to 68, it is generally consistent with the empirical distribution of sample sizes in psychology (Carter et al., 2019). Thus, I chose to randomly draw primary study sample sizes from the same meta-analytic dataset and assumed equal sample sizes between the experimental group and control group when generating individual participant data.

The degree of censoring ranged from 0.05 (extreme suppression) to 1 (no censoring). These values match those used in recent simulation studies (Chen & Pustejovsky, 2025; Mathur & VanderWeele, 2021; Rodgers & Pustejovsky, 2021). This broad range of selective publication enables us to examine the performance of different methods in the absence of selective publication and across varying degrees of censoring.

### Performance criteria

For each estimation method, I calculated the rejection rates of the one-sided hypothesis test for selective publication. The Type I error rates were rejection rates under conditions free of selective publication (λ=1) and were compared with the nominal level α=0.05. For the estimation methods that have close-to-nominal Type I error rates, I calculated the statistical power as the rejection rate under selective publication (λ<1). The Monte Carlo standard errors of the rejection rates with 5000 replications were less than 0.008 across all conditions, which indicates that the performance measures were calculated with high precision.

## Results

### Type I error rates

I present the Type I error rates of different methods based on one-sided tests for publication bias or small-study effects at the conventional level of α=0.05. Type I error rates were calculated for simulation conditions in the absence of selective publication (i.e., λ=1). All graphical results presented in the main text are from simulated meta-analyses with k=50 and $\{\rho _1, \rho _2\} = \{0.4, 0, -0.2, -0.4 \}$. The performance pattern of various methods in meta-analyses with different numbers of studies or levels of correlations aligns with those described in the main text. The online supplementary materials include complete simulation results and are available at https://osf.io/cjx75/files/2amb4.

**Fig. 1:**
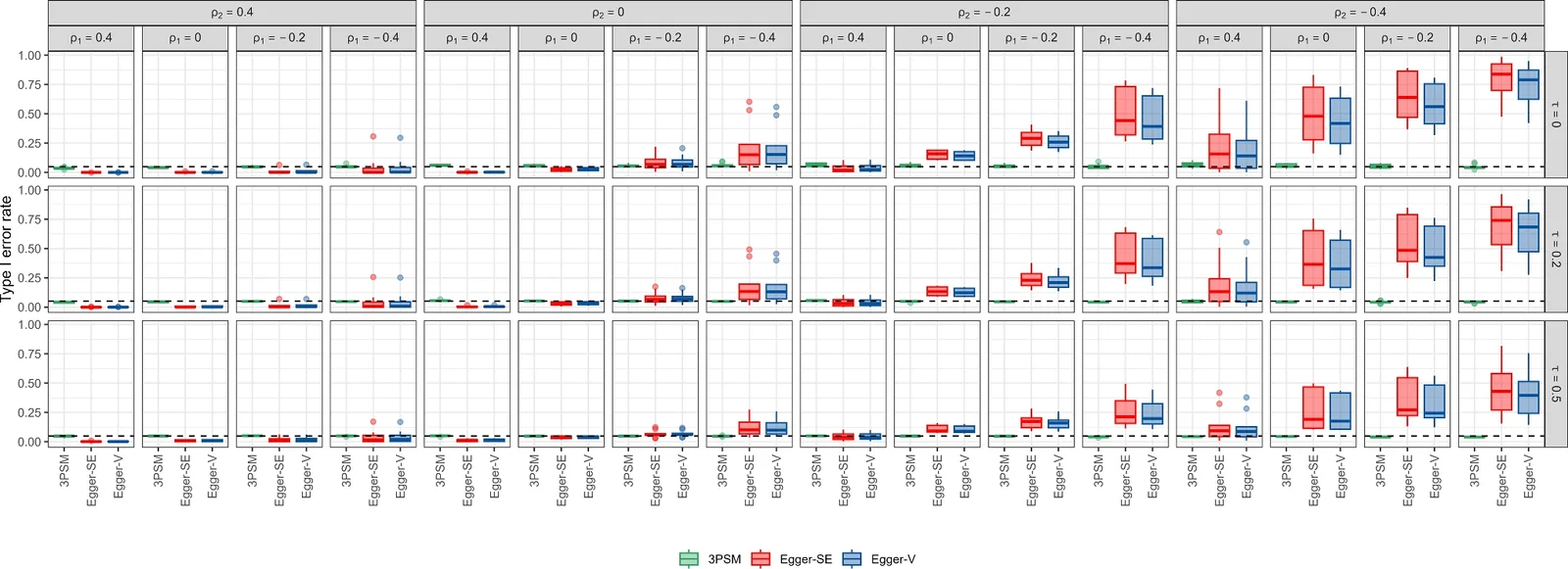
Type I error rates of different methods (*x*-axes) for testing selective publication when both covariates were omitted in the samples of k=50, $\beta _1 = \{0.1, 0.2, 0.5\}$, and $\beta _2 = \{0.1, 0.2, 0.5\}$. 3PSM = three-parameter selection model. Egger-SE = Egger’s regression with SE as the predictor. Egger-V = Egger’s regression with sampling variance as the predictor. $\rho _1$ = the correlation between the binary variable and primary study sample size (*N*). $\rho _2$ = the correlation between the continuous variable and sample size (*N*). τ = the between-study residual heterogeneity

#### Ignoring both covariates

Figure 1 displays the Type I error rates of the 3PSM likelihood-ratio test and Egger-type regression tests when both covariates were ignored for k=50. Rows correspond to different levels of residual heterogeneity and columns represent varying levels of confounding (i.e., correlation between covariates and the primary study sample size). Each boxplot represents the distribution of Type I error rates across $\{\beta _1, \beta _2\} = \{0.1, 0.2, 0.5\}$. Overall, the 3PSM likelihood-ratio test maintained near-nominal Type I error rates across conditions, whereas Egger-SE and Egger-V tended to have inflated rejection rates in the presence of confounding (i.e., the covariates were negatively correlated with the primary study sample size). The Type I error inflation for Egger-SE and Egger-V became more pronounced as the correlation between the covariates and sample size became increasingly negative; that is, the correlation between covariates and standard error became more positive. When the covariates became increasingly positively correlated with the sample size, and therefore negatively associated with the standard error, Egger-SE and Egger-V tended to have Type I error rates below the nominal level, whereas 3PSM showed only slightly deflated rejection rates. This pattern is expected for the one-sided test, especially in Egger-type regression tests that examine the association between effect size estimates and their standard errors. When covariates are positively associated with the true effect sizes but negatively associated with standard errors, omitting them may create a negative association between the observed effect size estimates and standard errors, making rejection less likely and regression tests more conservative. The 3PSM likelihood-ratio test was less affected because it directly evaluates evidence for selective publication of significant results.

In the absence of confounding ($\rho _1 = \rho _2 = 0$), the likelihood-ratio test from a 3PSM had Type I error rates at or mildly above the nominal level (below 0.10). The Egger-SE and Egger-V had rejection rates near or below the nominal level. The error rates of all methods were closer to the nominal level when the residual heterogeneity was large (τ=0.5) than when the residual heterogeneity was zero (τ=0). Across varying levels of residual heterogeneity, as the strength of confounding increased, the 3PSM likelihood-ratio test tended to maintain the rejection rates well across most conditions, while Egger-SE and Egger-V exhibited substantially inflated Type I error rates. When the confounding was substantial (e.g., $\rho _1 = \rho _2 = -0.6$), the regression tests yielded error rates ranging from 0.50 to 1.00 for small or null residual heterogeneity (see Supplementary Section S1.1.1 for more detail). At a given level of confounding, the extent of Type I error inflation in regression tests was smaller for larger residual heterogeneity.

**Fig. 2:**
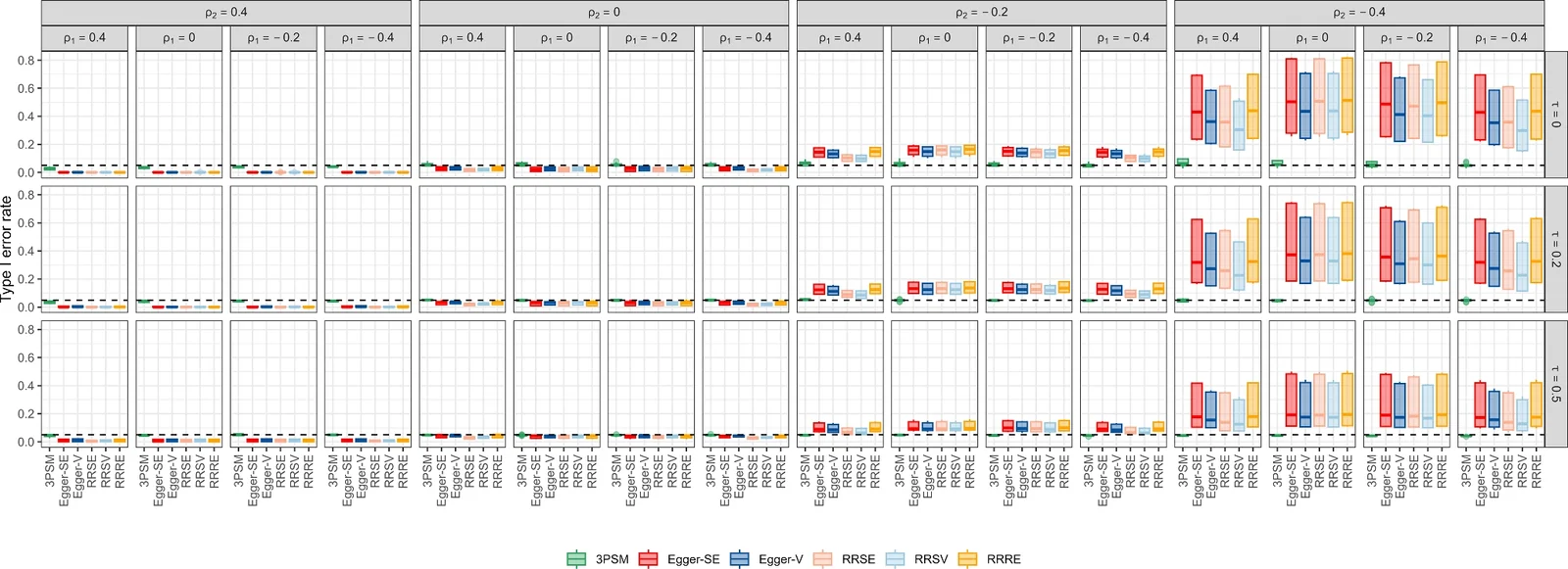
Type I error rates of different methods (*x*-axes) for testing selective publication when only the binary covariate was incorporated in the samples of k=50. 3PSM = three-parameter selection model. Egger-SE = Egger’s regression with SE as the predictor. Egger-V = Egger’s regression with sampling variance as the predictor. RRSE = residual regression with modified standard error. RRSV = residual regression with modified sampling variance. RRRE = residual regression with residualized standard error. $\rho _1$ = the correlation between the binary variable and primary study sample size (*N*). $\rho _2$ = the correlation between the continuous variable and sample size (*N*). τ = the between-study residual heterogeneity

The supplementary materials include graphical results for varying numbers of studies, slope parameters, and correlations. The Egger-SE and Egger-V methods exhibited increasing Type I error inflation as confounding increased for varying values of k. The inflation was more severe in meta-analyses with small residual heterogeneity (e.g., τ=0 or 0.2) and a large number of studies (e.g., k=80). In contrast, the 3PSM likelihood-ratio test controlled Type I error across the number of studies. Note that the error rates of 3PSM were slightly above nominal when residual heterogeneity was small in meta-analyses with k=20 and k=50, but were at or below nominal for k=80. In the absence of selective publication, the mild inflation of 3PSM likelihood-ratio test was observed when the residual heterogeneity, the number of studies, and covariate slopes were all small. The Type I error rates of 3PSM test tended to be smaller and even below nominal as the residual heterogeneity, number of studies, and covariate slopes increased. In the absence of selective publication and confounding, the Egger-SE and Egger-V had Type I error rates at or below the nominal level. However, in the presence of confounding, the regression tests generally exhibited substantially inflated Type I error rates, and the inflation was worse under a large number of studies, large covariate slopes, and small residual heterogeneity.

#### Ignoring the continuous covariate

Figure 2 displays the Type I error rates of different tests when the binary covariate was incorporated, but the continuous variable was unmodeled in meta-analyses with k=50 studies for varying levels of residual heterogeneity (rows) and degrees of confounding (columns). The boxplots summarize Type I error rates across the positive slope parameters (i.e., $\{\beta _1, \beta _2\} = \{0.1, 0.2, 0.5\}$). Generally speaking, the 3PSM likelihood-ratio test was more robust to selective publication than the regression-based methods when the continuous confounding covariate was unmodeled. The Type I error rates of the 3PSM likelihood-ratio test were close to, or mildly above, the nominal level across conditions. However, for the regression-based methods, modeling the binary covariate removed the impact of the binary covariate correlation ($\rho _1$) on error rates, while ignoring the continuous confounding covariate resulted in inflated Type I error rates when $\rho _2 < 0$ and deflated Type I error rates when $\rho _2 > 0$.

**Fig. 3:**
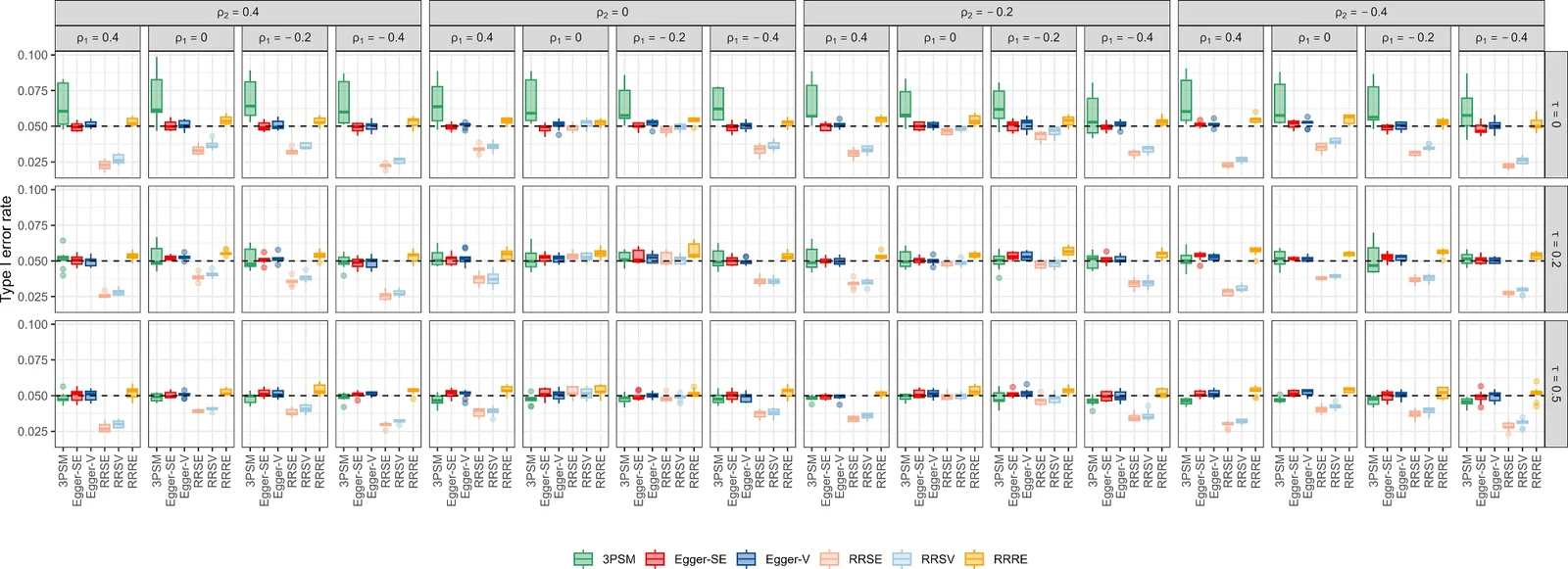
Type I error rates of different methods (*x*-axes) for testing selective publication when both confounding covariates were accounted for in the samples of k=50. 3PSM = three-parameter selection model. Egger-SE = Egger’s regression with SE as the predictor. Egger-V = Egger’s regression with sampling variance as the predictor. RRSE = residual regression with modified standard error. RRSV = residual regression with modified sampling variance. RRRE = residual regression with residualized standard error. $\rho _1$ = the correlation between the binary variable and primary study sample size. $\rho _2$ = the correlation between the continuous variable and sample size. τ = the residual heterogeneity

In the absence of continuous covariate confounding (i.e., $\rho _2 = 0$), the 3PSM likelihood-ratio test had Type I error rates near the nominal level (below 0.08). A slight inflation existed particularly under null (τ=0) or small residual heterogeneity (τ=0.2). When $\rho _2 = 0$, the Egger-type regression tests and residual analyses had Type I error rates slightly below the nominal level for varying values of $\rho _1$. This deflation was stronger for the residual regression of modified standard error (RRSE) or sampling variance (RRSV) than Egger-SE, Egger-V, or the residual regression of residualized standard error (RRRE), especially when $\rho _1 = -0.4$ or -0.6. When the negative association between the continuous covariate and sample size became stronger, the Type I error rates of Egger-type regression tests and residual analyses grew fast above nominal, while the 3PSM likelihood-ratio test calibrated Type I error well. The Type I error inflation in RRSE and RRSV was less pronounced than Egger-SE, Egger-V, or RRRE because they underestimated the relation between the effect size estimate and standard error. When the continuous covariate was positively associated with sample size ($\rho _2 = 0.4$ or 0.2), Egger-type regression tests and residual analyses consistently showed deflated Type I error rates, especially under conditions with less confounding from the binary covariate. These regression-based tests became increasingly conservative as residual heterogeneity decreased, with rejection rates approaching zero. In contrast, the 3PSM likelihood-ratio test was less sensitive to the positive correlation between the continuous covariate and sample size.

The supplementary materials include graphs of simulation results for different numbers of studies (k) and covariate slopes ($\beta^{\prime }s$). In the absence of selective publication, the 3PSM likelihood-ratio test that modeled only the binary covariate had mildly inflated error rates when the residual heterogeneity, covariate slopes, and the number of studies were small, a pattern similar to the scenarios where both covariates were ignored. However, compared to ignoring both covariates, controlling for the binary covariate reduced the mildly inflated Type I error rates under these conditions only when the confounding of the binary covariate existed (i.e., $$\rho _1 < 0$$). When the correlation between the binary covariate and N was zero ($$\rho _1 = 0$$), incorporating the binary covariate resulted in slightly worse Type I error rates for the 3PSM test. The pattern of results of Egger-type regression and residual analyses for meta-analyses with k=20 or 80 studies was similar to those of k=50. It is worth noting that Egger-type regression and residual analyses incorporating the binary covariate exhibited consistent improvement in controlling Type I error rates when the correlation between the binary covariate and N was negative ($$\rho _1 < 0$$). When $$\rho _1 \ge 0$$, accounting for the binary covariate in Egger-SE, Egger-V, or residual analyses led to worse Type I error inflation than ignoring the covariate, especially when the binary variable slope was large ($$\beta _1 = 0.5$$).

**Fig. 4:**
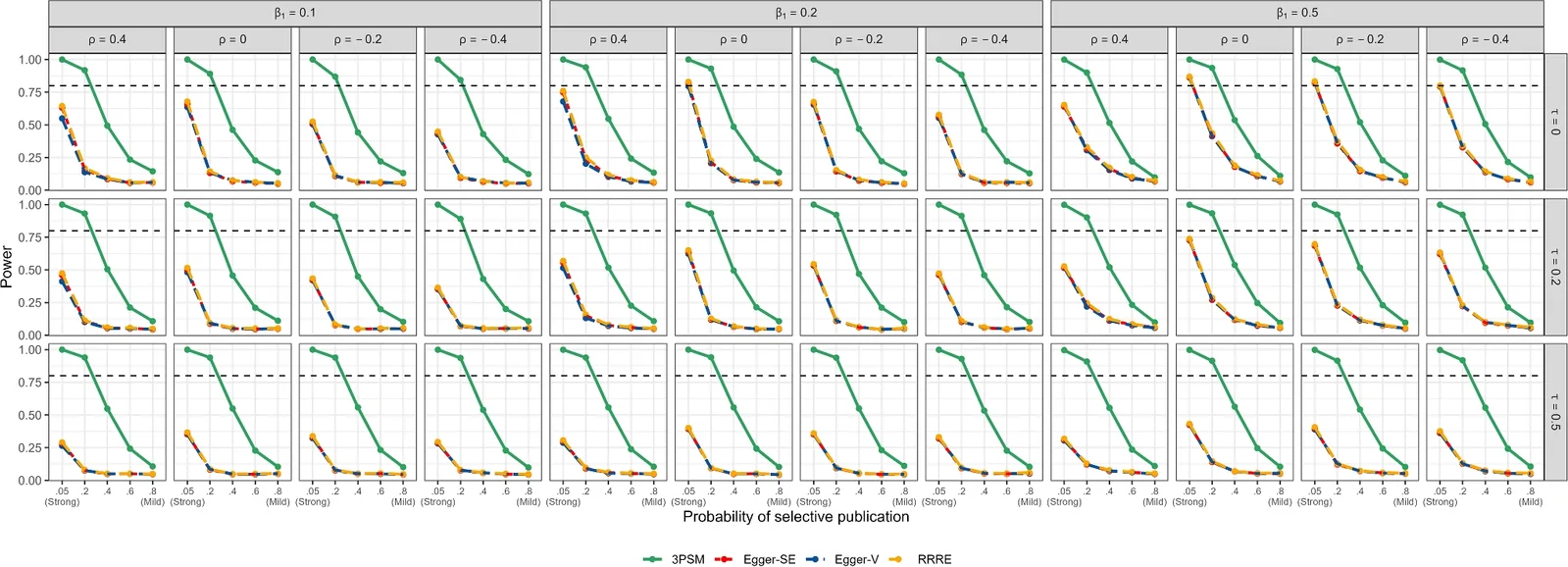
Power of different methods for testing selective publication when both covariates were accounted for in the samples of k=50 and $\beta _2 = 0.1$. 3PSM = three-parameter selection model. Egger-SE = Egger’s regression with SE as the predictor. Egger-V = Egger’s regression with sampling variance as the predictor. RRRE = residual regression with residualized error. ρ = the correlation between primary study sample size and both covariates. $\beta _1$ = slope coefficient of the binary covariate. τ = the residual heterogeneity

#### Modeling both covariates

Figure 3 shows the Type I error rates of the 3PSM likelihood-ratio test, Egger-SE, Egger-V, and residual analyses when both covariates were incorporated in the analyses for meta-analyses with k=50 studies. The boxplots summarize Type I error rates over all positive values of $\beta _1$ and $\beta _2$. The likelihood-ratio test of the 3PSM had inflated Type I error rates when the residual heterogeneity was zero but tended to maintain the Type I error rates near or below the nominal level when the residual heterogeneity existed (τ=0.2,0.5). The deviation of rejection rates from nominal was greater for a small number of studies and covariate slopes (see Supplementary Section S1.3 for more details). The Egger-SE and Egger-V maintained Type I error rates generally well across conditions. When the correlations between each covariate and primary study sample size were zero, the residual analyses with modified standard error or sampling variances had close-to-nominal Type I error rates. However, in the presence of confounding, these residual analyses tended to have rejection rates below the nominal level, especially under strong confounding. The residual analysis regressing residualized effect estimates on residualized standard errors (RRRE) performed similarly to Egger-SE, but with slightly inflated Type I error rates due to the differences in the estimated residual heterogeneity.

The Supplement Section S2 provides graphs for simulations with different covariate slopes ($\beta^{\prime }s$) and numbers of studies (k) when both covariates were modeled. Compared with ignoring both covariates or including only the binary covariate, modeling both of them in Egger-SE, Egger-V, and RRRE produced Type I error rates consistently closer to the nominal level across conditions. Incorporating both confounding covariates that are negatively associated with sample size in the 3PSM likelihood-ratio test mitigated the mild Type I error inflation only when the residual heterogeneity, number of studies, and covariate slopes were small (e.g., τ=0, k=20, $$\beta _1 = 0.1$$).

### Power

I now discuss the relative power of different tests for detecting selective publication when both covariates were modeled for k=50 and $\beta _2 = 0.1$. I only examine the statistical power when both confounding covariates were included because most regression methods had well-calibrated Type I error rates. I do not consider the residual regression with modified standard error or sampling variance because they were more conservative compared to Egger-type regression tests. When the residual heterogeneity, the number of studies, and covariate slope parameters were all small, the power of the 3PSM likelihood-ratio test should be interpreted with caution due to inflated Type I error rates. The Supplementary Section S3 includes results comparing the relative power of different tests for varying numbers of studies. The pattern of power rates across different methods was very similar regardless of the number of studies included in meta-analyses, although larger number of studies generally resulted in higher power for all tests.

Figure 4 depicts the power rates of different tests when controlling for both covariates, across different levels of selective publication (horizontal axis) for meta-analytic samples with k=50 studies. Rows represent the residual heterogeneity, and columns represent the level of confounding and the binary variable slope. For illustrative purposes, I only include the results under conditions where the correlations between both covariates and the primary study sample size were the same. Across degrees of selective publication, the likelihood-ratio test of 3PSM consistently had higher power than Egger-SE, Egger-V, or the residual analysis with residualized standard error (RRRE). This advantage was more pronounced under conditions where 3PSM controlled Type I error rates well (e.g., τ=0.5) compared to conditions where 3PSM exhibited mild inflation (e.g., τ=0). However, the power rates of 3PSM were only adequate (above the conventional level of 80%) for strong to extreme selection (i.e., λ=0.05 or 0.2). Across varying probabilities of selective publication, the Egger-SE, Egger-V, and RRRE had adequate power only under extreme censoring when the residual heterogeneity was zero, and the binary covariate slope was large. They had limited power (below 80%) in other scenarios, and the power was lower for weaker selective publication, stronger confounding, and higher residual heterogeneity.

## Discussion

The present study was designed to examine the performance of available methods for detecting selective publication in meta-analyses with covariates correlated with the effect size estimate and its precision. Researchers have recommended considering the confounding effects when testing for small-study effects or potential selective publication (Nakagawa et al., 2022; Peters et al., 2010; Sterne & Egger, 2005). However, there has been little methodological evaluation of available approaches for detecting selective publication while accounting for confounding. The current simulation study fills this gap by investigating the performance of two Egger-type regression tests, residual analyses, and the 3PSM likelihood-ratio test to detect selective publication in the presence of both binary and continuous covariates. Overall, this study demonstrates that Egger-type regression tests and the 3PSM likelihood-ratio test offer complementary strengths under the one-step *p* value selection mechanism. Regression-based tests provide slightly better Type I error control when relevant covariates are modeled, whereas the 3PSM achieves greater power and is more robust to omitted confounders.

The results of this study have shown that ignoring covariates that were positively correlated with both effect size estimates and standard errors resulted in inflated Type I error rates in Egger-type regression tests.^3^ This is because the confounding led to spurious associations between effect size estimates and standard errors (Peters et al., 2010; Sterne & Egger, 2005). Partial adjustment, including the binary covariate while ignoring the continuous one, did not fully address this inflation. In contrast, the 3PSM likelihood-ratio test generally maintained close-to-nominal Type I error rates across conditions, even when one or both confounding covariates were ignored. The likelihood-ratio test of 3PSM was more robust to the confounding than regression-based tests, likely because it tests selective publication directly under a selection mechanism aligned with the data-generating process (Pustejovsky & Rodgers, 2019). It should be noted that ignoring confounding covariates in the 3PSM likelihood-ratio test can result in slightly inflated Type I error rates when the residual heterogeneity, the number of studies, and slope coefficients were all small.

When both confounding covariates were included, the Egger-type regression tests generally maintained Type I error at the nominal level, whereas the 3PSM likelihood-ratio test had slight Type I error inflation when residual heterogeneity and number of studies were small. Although Egger-SE and Egger-V were well-calibrated when both relevant covariates were modeled, the 3PSM likelihood-ratio test achieved higher power rates across varying degrees of selective publication. The advantage of 3PSM in detecting selective publication when it truly exists was also noted in prior simulation studies that did not include covariates under the one-step *p* value selection (Carter et al., 2019; Pustejovsky & Rodgers, 2019). Additional analyses presented in Supplementary Section S4 showed a similar pattern in the performance of Egger-SE and 3PSM when covariates unrelated to true effect sizes were included. Egger-SE remained well-calibrated regardless of whether irrelevant covariates were modeled, with only a minimal reduction in power. In contrast, the 3PSM likelihood-ratio test consistently exhibited higher power than Egger-SE but showed slight Type I error inflation when the number of studies and residual heterogeneity were small.

The simulations also compared the performance of Egger-type tests and residual analyses that adjust for the effects of confounding. When both covariates were included as control predictors in meta-regression models, the residual analyses that removed only the correlations between covariates and effect size estimates tended to be conservative with below-nominal Type I error rates. This is expected because the association between the effect size estimate and its standard error was underestimated, making it less likely to reject the null hypothesis (Freckleton, 2002). The residual analyses that removed the confounding effects of covariates on the associations between effect size estimates and standard errors tended to perform similarly to Egger-SE or Egger-V. For meta-analysts who use regression-based tests for addressing small-study effects or selective publication, I recommend using Egger-type regression tests (e.g., Egger-SE and Egger-V) rather than residual analyses because these methods have similar performance and Egger-type tests are more straightforward to implement and interpret.

### Limitations and future directions

This simulation study has several limitations that warrant further investigation. First, the current simulation study evaluated the performance of Egger-type regression tests, residual analyses, and the 3PSM likelihood-ratio test under the one-step *p* value selection mechanism that may be in favor of 3PSM. Therefore, the relatively strong performance of 3PSM should be interpreted in relation to the specific selection mechanism considered in the data-generating process. Although *p* value-based selection mechanisms have been commonly assumed and examined in prior simulation studies (e.g., Carter et al., 2019; McShane et al., 2016) and have been recommended for addressing publication bias (Wu et al., 2026), other selection mechanisms (Copas, 1999) or more general questionable research practices (QRPs, Carter et al., 2019; Nagy et al., 2025) should be considered for a more comprehensive evaluation of these methods. Although both 3PSM and regression-based methods showed reduced performance under QRPs in Carter et al. (2019), with 3PSM generally outperforming PET-PEESE, it remains an open question how these methods perform in the presence of confounding and other types of selection strategies. A related limitation is that the selection mechanism might operate differently depending on study characteristics (Coburn & Vevea, 2015; Vevea & Hedges, 1995). A more general data-generating model could allow both the effect size distribution and the selective publication process to vary as a function of study-level covariates (Peters et al., 2010). Future research could examine the consequences of ignoring such moderators of the selection process when estimating selection parameters and addressing selective publication.

Second, the current study explored the detection of selective publication in meta-analysis where each study contributes a single effect size estimate and these effect sizes are independent across studies. However, it is common that meta-analyses in education, psychology, and other fields include primary studies with multiple, statistically dependent effect sizes (Tipton et al., 2019; Wu et al., 2026). Although it is possible to include covariates in the multivariate adapted PET/PEESE or in the selection models (Pustejovsky et al., 2025), it remains an open question how other regression-based methods (e.g., the endogenous regression model, Bom and Rachinger (2019) can incorporate confounding covariates (Chen & Pustejovsky, 2025). Future research should explore ways to address confounding in meta-analytic methods for dependent effect sizes and examine the performance of various methods in handling selective reporting bias in the presence of both explainable and residual heterogeneity.

Third, the current simulation study focused on detecting selective publication rather than correcting for publication bias. Although evaluating the Type I error rates and statistical power of tests for selective publication is important, it is also important to examine how different bias-adjustment methods perform in recovering the average effects and moderator effects in meta-analyses with confounding covariates. Because publication bias testing and correction are closely related, future research should investigate the performance of regression-based adjustment methods and selection models for estimating average effects and moderator effects when some moderators are correlated with study precision. Based on the current findings, I expect that omitted confounding covariates that induce spurious detection of selective publication and inflate Type I error rate of regression-based tests are also likely to bias the PET-PEESE average effect estimate downward. In contrast, the implications of omitted confounding covariates for average effect estimation in the 3PSM are less direct and warrant further investigation.

Finally, the current study did not consider missingness in the covariates. In practice, meta-analysts often include primary studies that involve missing covariates. A variety of statistical methods are available for handling missing covariates in meta-analysis (Lee & Beretvas, 2023). However, it has not been examined how to combine different methods for selective publication with approaches for handling missing covariates in meta-analyses (Pellegrini et al., 2025). Considering the importance of incorporating covariates in assessing and potentially adjusting for selective reporting and publication bias, it is important for future research to explore methods for addressing publication bias, confounding, and missing covariates simultaneously.

### Implications for practice

The findings from this study have several implications for how meta-analysts should assess selective publication in the presence of between-study heterogeneity. Meta-analysts should include covariates that might confound the association between the effect size estimate and standard error in Egger-type regression tests. Ignoring confounding covariates can result in inflated Type I error rates, meaning that regression-based tests tend to identify selective publication when it does not exist. This finding is important to highlight because it is still rare for researchers to include study characteristics that might confound the relation between the effect estimate and standard error in the assessment of selective publication (Wu et al., 2026). When multiple covariates are available to be incorporated in the evaluation of selective publication, I recommend that meta-analysts model all the covariates simultaneously instead of incorporating them separately.

Although residual analyses provide an alternative way to control for confounding covariates, they did not outperform Egger-SE or Egger-V in maintaining Type I error rates or achieving adequate statistical power. This is true even when the correlations between the confounding covariates and both the effect size estimates and their standard errors were removed. Thus, I do not recommend residual analyses for statistical tests of selective publication. Given that meta-regression is widely used to explore between-study heterogeneity, meta-analysts should include potential confounding covariates as predictors in the regression-based tests for selective publication.

The likelihood-ratio test from 3PSM offers another alternative to test for selective publication while accounting for covariates. Compared with Egger-SE and Egger-V, the 3PSM likelihood-ratio test generally achieved higher statistical power under one-step *p*-value selection, although all methods had limited power under most conditions. However, 3PSM exhibited slight Type I error inflation when residual heterogeneity and covariate slopes were small, whereas Egger-SE and Egger-V controlled Type I error rates well under these conditions.

To sum up, the findings of this study suggest that plausible confounding covariates should be explicitly modeled when assessing selective publication using Egger-type regression tests. Residual analyses offer no clear advantages over directly modeling covariates and therefore are not recommended as a default strategy. Because neither Egger-type regression tests nor 3PSM performed optimally across all conditions, I recommend using both approaches as complementary analyses and interpreting their results in light of their respective strengths and limitations.

## Supplementary Information

Below is the link to the electronic supplementary material.

Supplementary file 1 (pdf 3568 KB)

## Data Availability

The simulation datasets and supplementary materials are available on the Open Science Framework at https://osf.io/cjx75.

## Footnotes

1 Nakagawa and colleagues described the difference between marginal residual and conditional residual in meta-regression with dependent effect sizes (Nakagawa et al., 2022). In the independent meta-analysis context, the distinction between these two types of residuals is less of a concern because the study-level heterogeneity is assumed to be independent of sampling error. Thus, I don’t examine the conditional residual approach in the current study.

2 One may also investigate a nonlinear relationship between effect size residuals and standard error residuals by (1) calculating the residuals of a regression model that regresses effect size estimates on covariates and the residuals of a regression model that regresses sampling variances on the same set of covariates, and (2) regressing effect size residuals on the sampling variance residuals. This is theoretically equivalent to Egger-V. For the sake of simplicity, I only illustrate in the simulations the extended residual analysis assuming a linear association.

3 Ignoring covariates that were positively related to effect size estimates but negatively correlated with standard errors (i.e., positively correlated with primary study sample sizes) resulted in deflated Type I error rates in Egger-type regression tests.

## Code availability

The code for replicating the Monte Carlo simulation study is available on the Open Science Framework at https://osf.io/cjx75.
