# Supplementary material for: Evaluating methods for testing publication bias in meta-analyses with confounding covariates: A simulation study
Source: Behav Res Methods. 2026 Oct 21;58(12):330. doi: 10.3758/s13428-026-03206-7 (PMC13687140; doi:10.3758/s13428-026-03206-7)
Supplement: Supplementary file 1 [file 13428_2026_3206_MOESM1_ESM.pdf]

Supplementary materials for 'Evaluating methods for testing publication bias in meta-analyses with confounding covariates: A simulation study'

Contents

S1 Type I error rates comparing across methods **2**

S1.1 Ignoring both covariates . . . 2

S1.2 Modeling the binary covariate when $\beta_1 \neq 0$ and $\beta_2 \neq 0$ . . . 6

S1.3 Incorporating both covariates when $\beta_1 \neq 0$ and $\beta_2 \neq 0$ . . . 10

S2 Type I error rates of each method **14**

S2.1 3PSM . . . 14

S2.2 Egger-SE . . . 15

S2.3 Egger-V . . . 16

S2.4 RRSE . . . 17

S2.5 RRRE . . . 18

S3 Power when both covariates were modeled **19**

S3.1 $\beta_2 = 0.1$ . . . 19

S3.2 $\beta_2 = 0.2$ . . . 21

S3.3 $\beta_2 = 0.5$ . . . 22

S4 Model misspecification **24**

S4.1 Type I error rates when $\beta_1 = \beta_2 = 0$ . . . 24

S4.2 Power when $\beta_1 = \beta_2 = 0$ . . . 27

S4.3 $\beta_1 = 0$, $\beta_2 \neq 0$ . . . 32

S1 Type I error rates comparing across methods

S1.1 Ignoring both covariates

S1.1.1 Varying k when $\beta_1 \neq 0$ and $\beta_2 \neq 0$

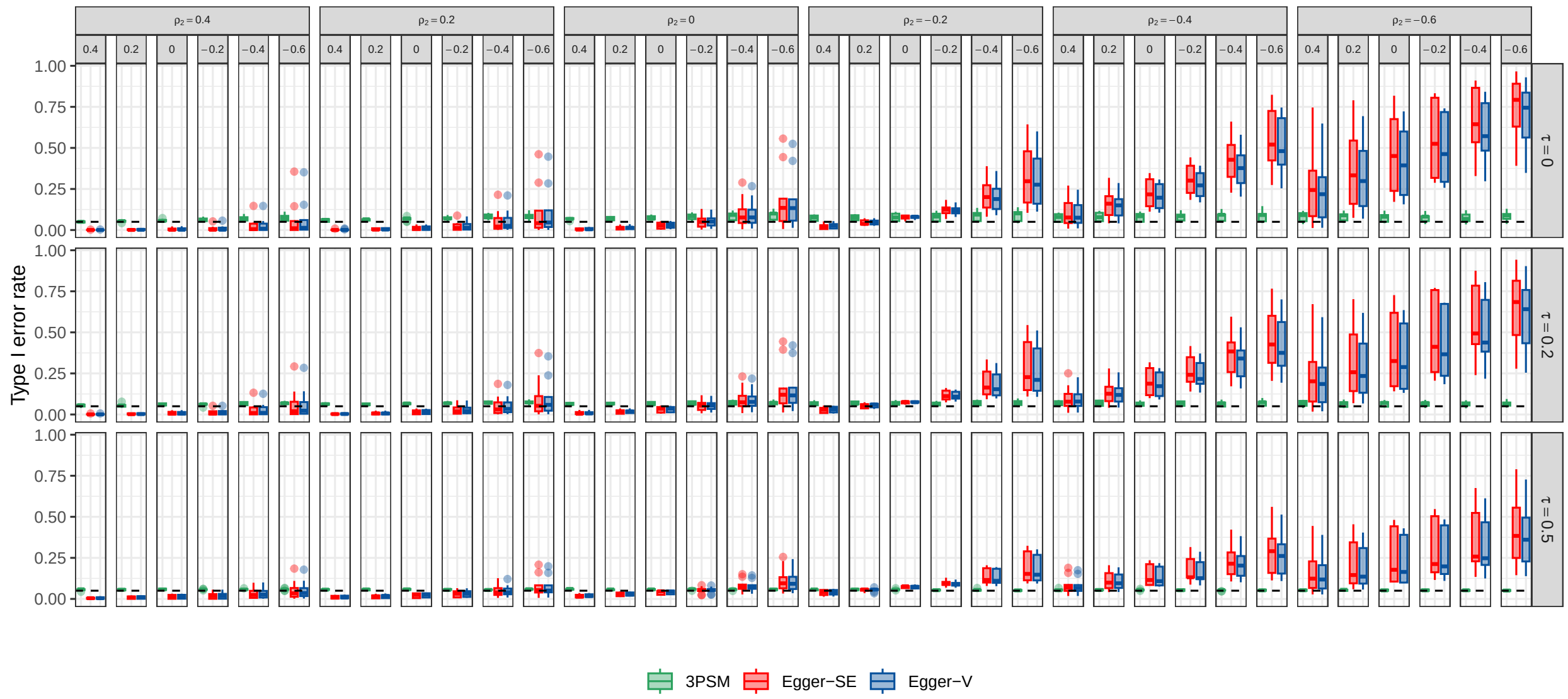

Figure S1: Type I error rates when both covariates were ignored in the sampels of $k = 20$.

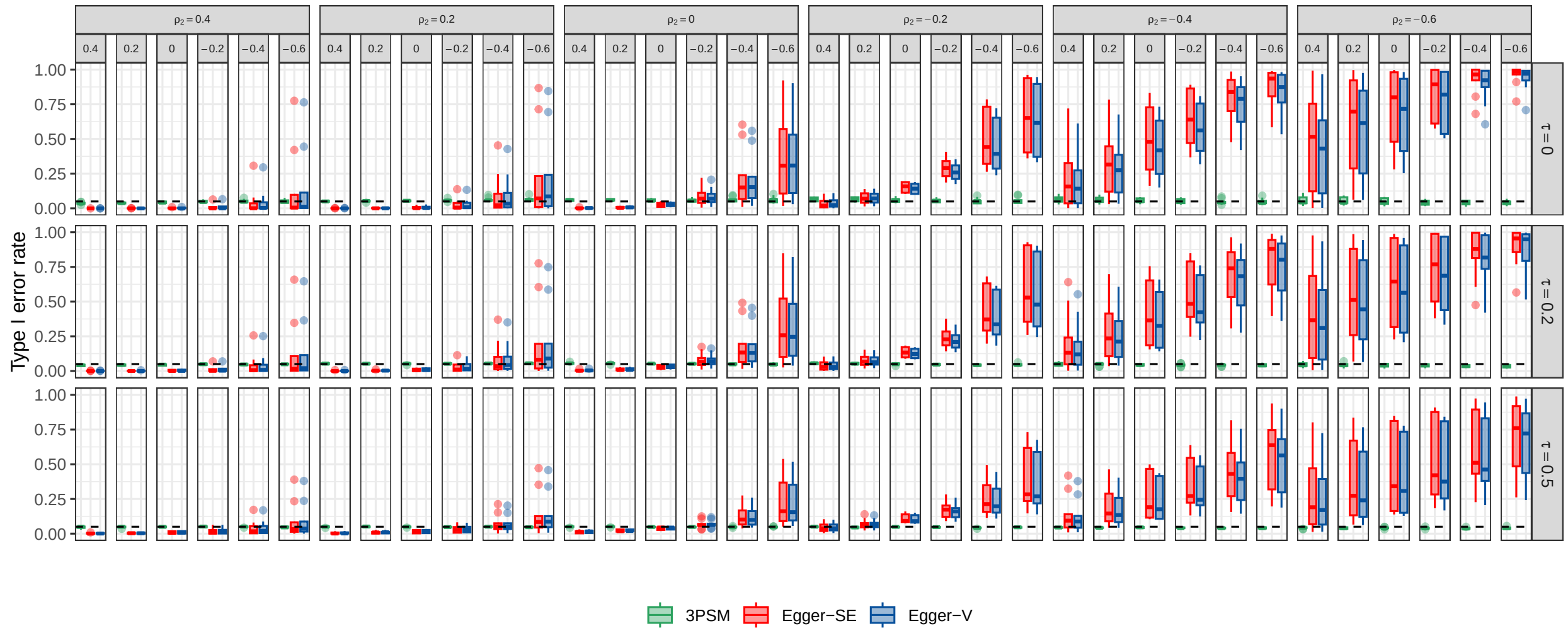

Figure S2: Type I error rates when both covariates were ignored in the sampels of $k = 50$.

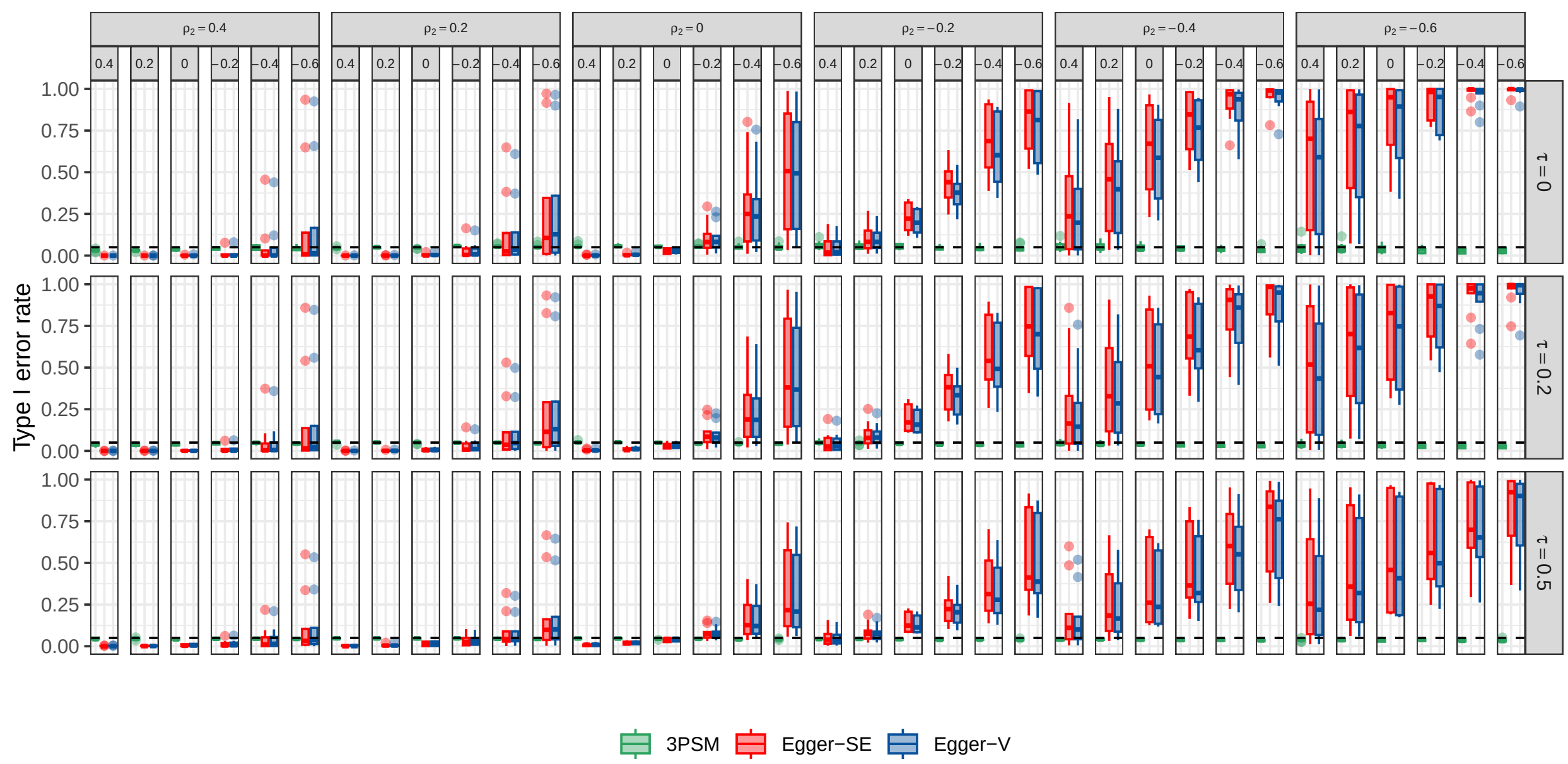

Figure S3: Type I error rates when both covariates were ignored in the sampels of $k = 80$.

S1.1.2 Varying β

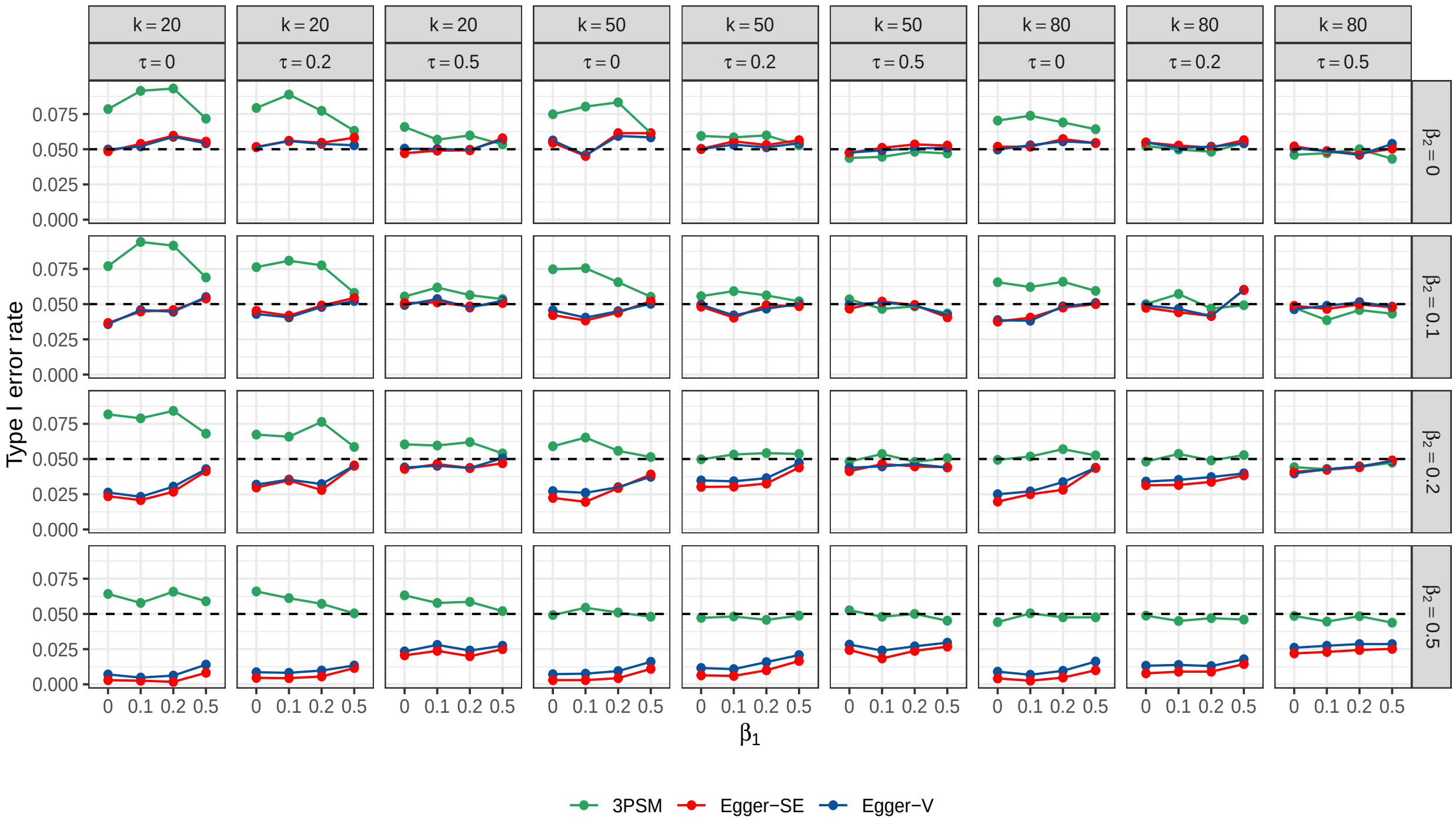

Figure S4: Type I error rates when both covariates were ignored in the absence of confounding ($\rho_1 = \rho_2 = 0$).

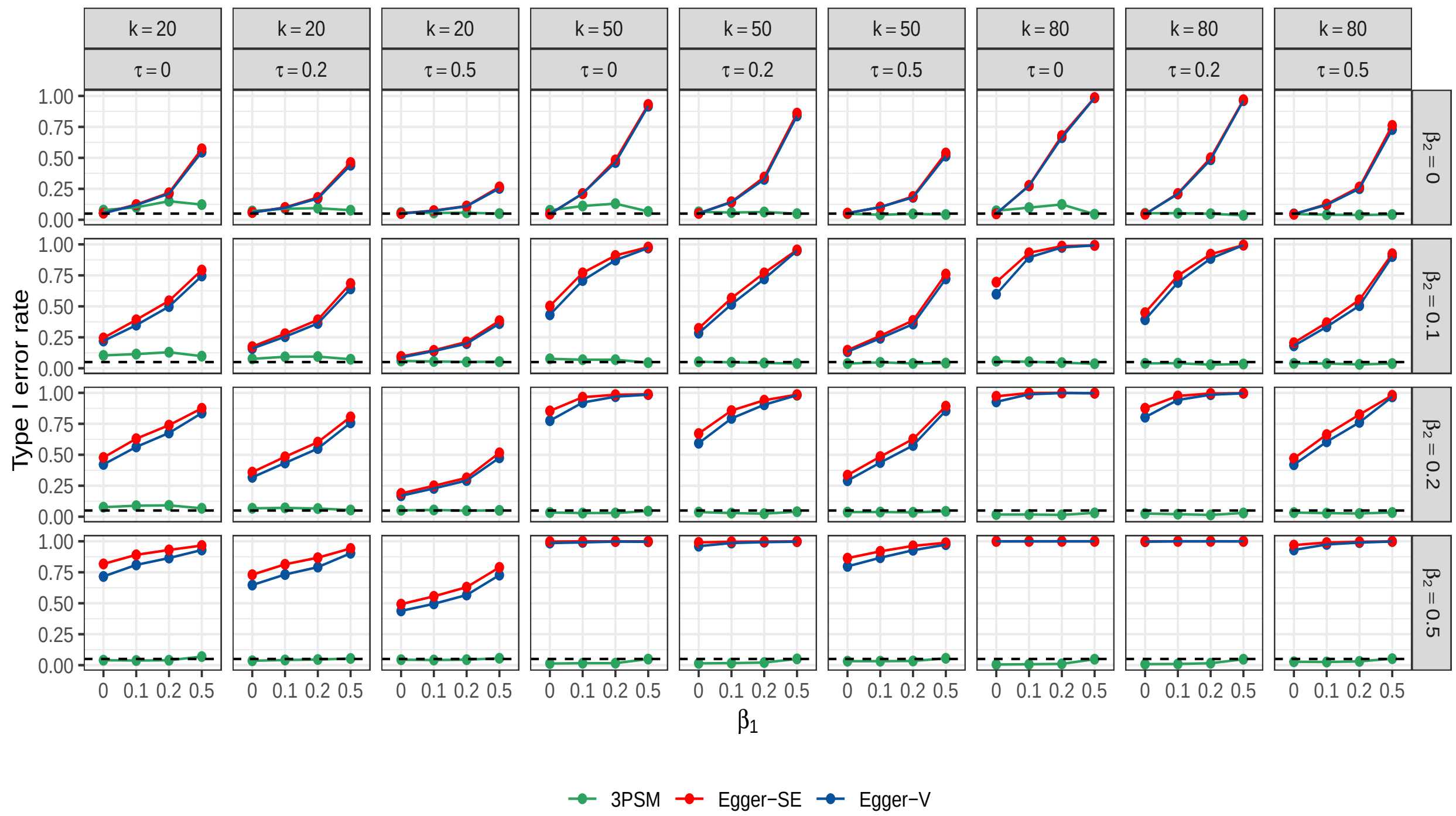

Figure S5: Type I error rates when both covariates were ignored with strong confounding ($\rho_1 = \rho_2 = -0.6$).

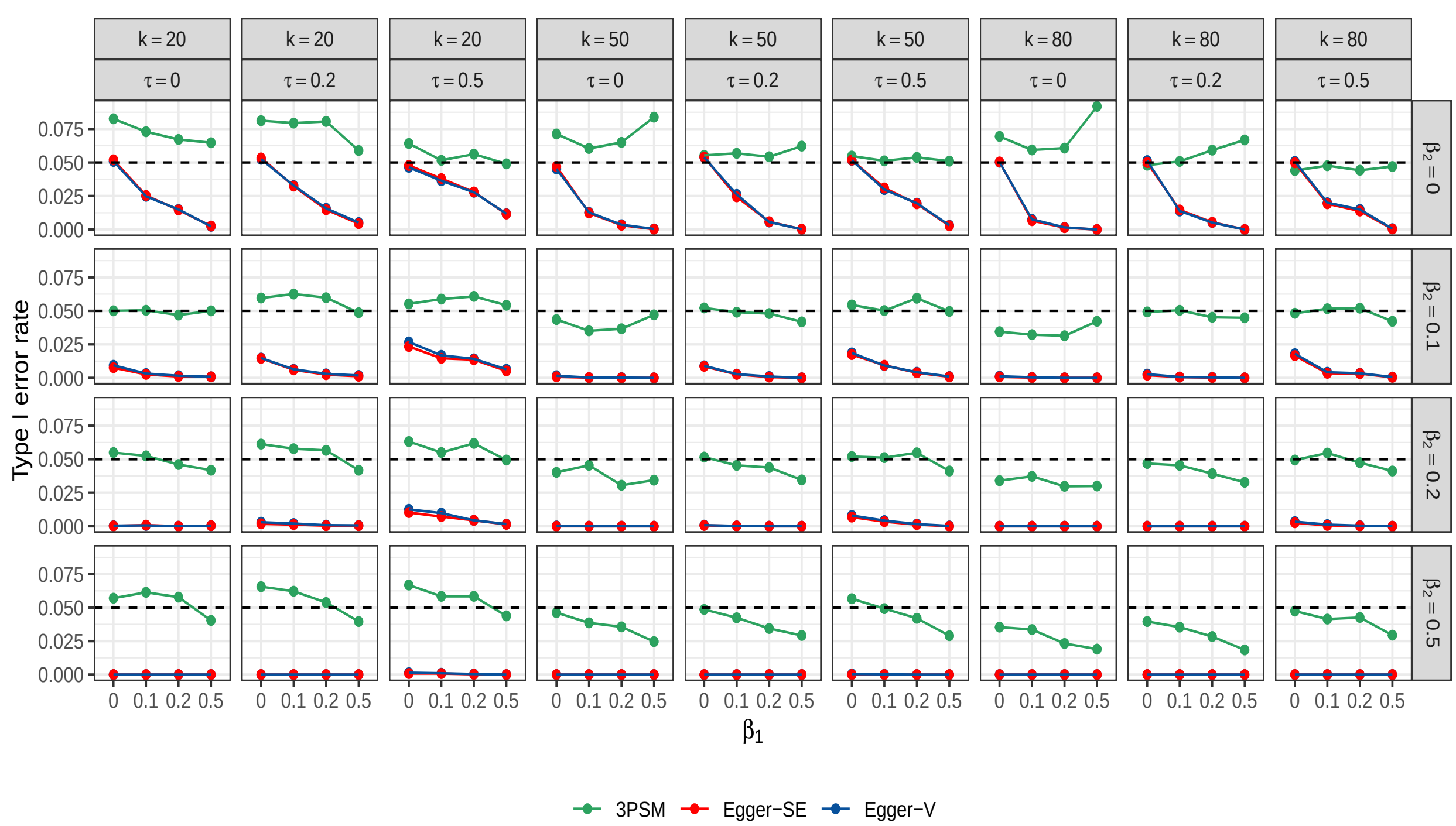

Figure S6: Type I error rates when both covariates were ignored with mixed confounding ($\rho_1 = \rho_2 = 0.4$).

S1.2 Modeling the binary covariate when $\beta_1 \neq 0$ and $\beta_2 \neq 0$

S1.2.1 Varying k

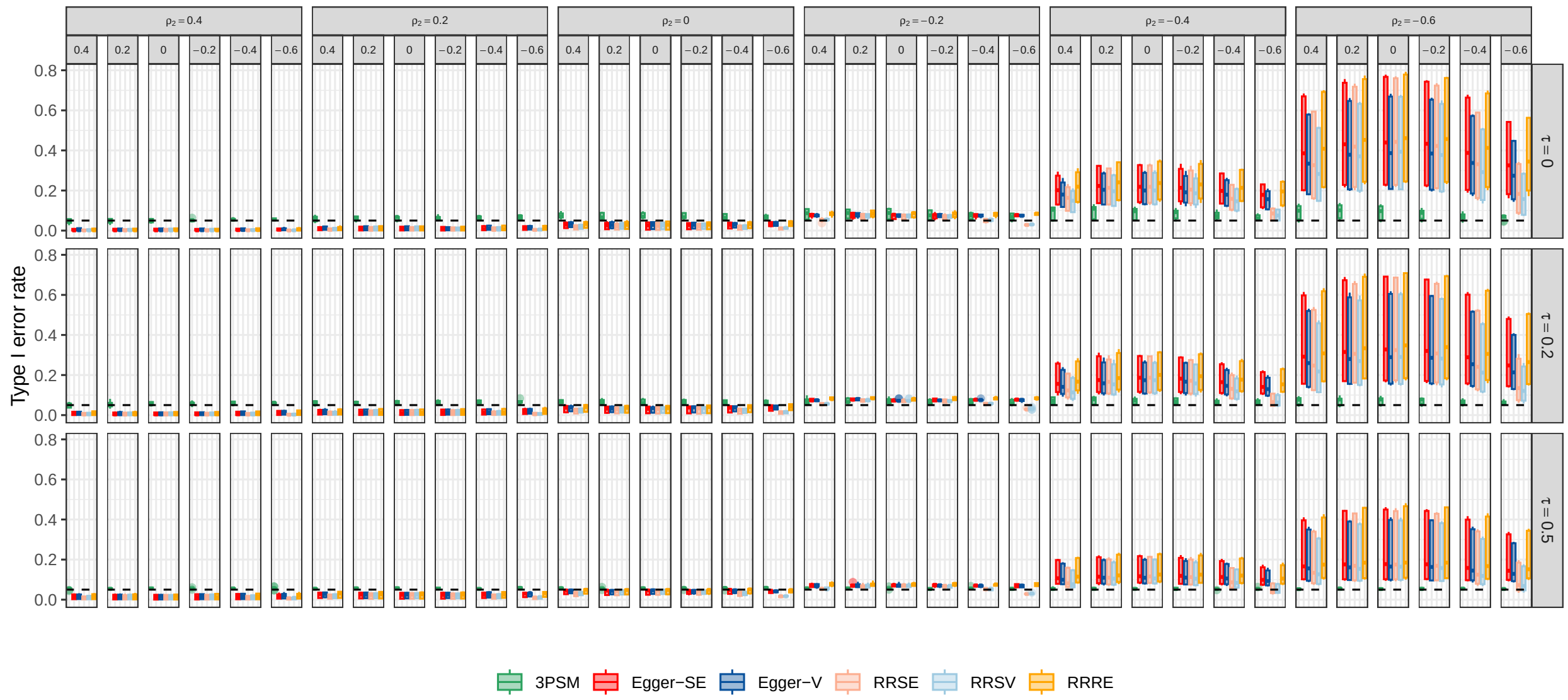

Figure S7: Type I error rates when the binary covariate was modeled in the sampels of $k = 20$.

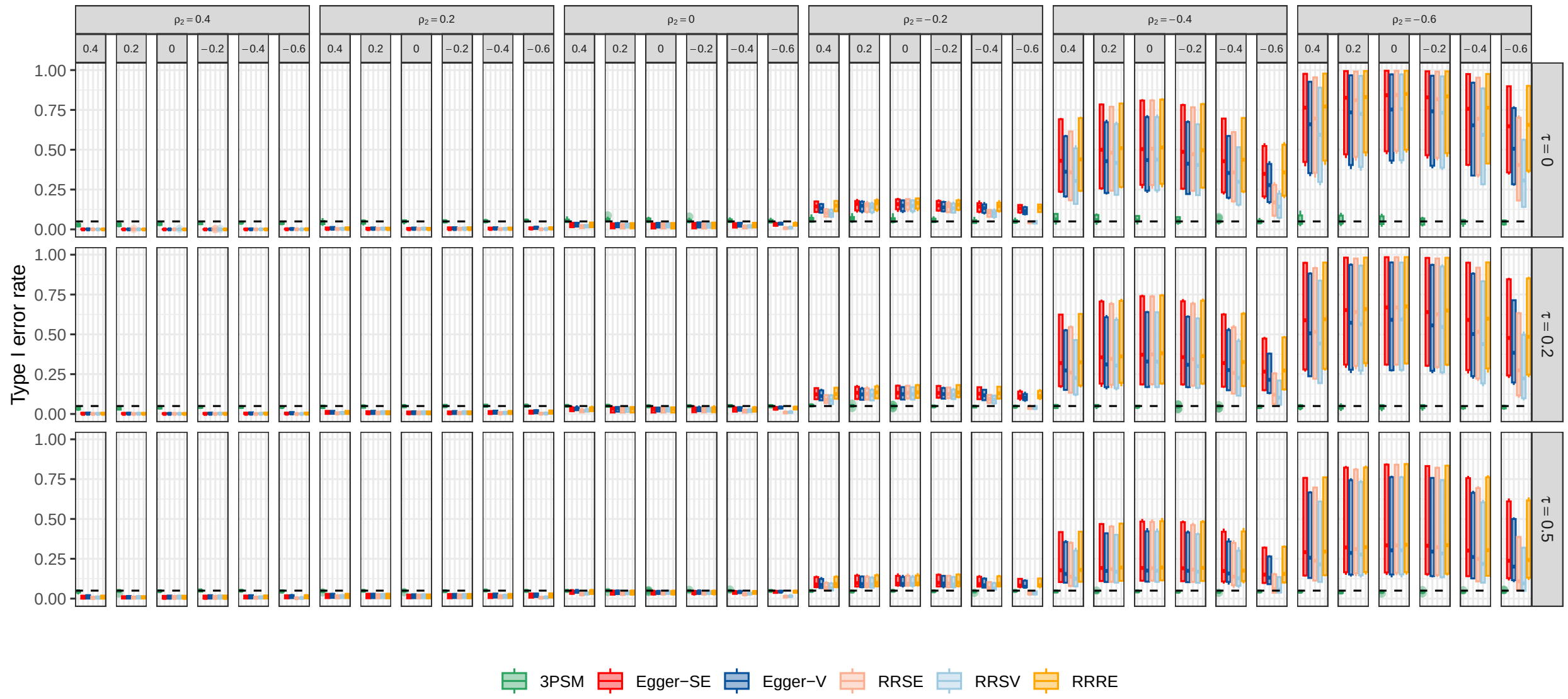

Figure S8: Type I error rates when the binary covariate was modeled in the sampels of $k = 50$.

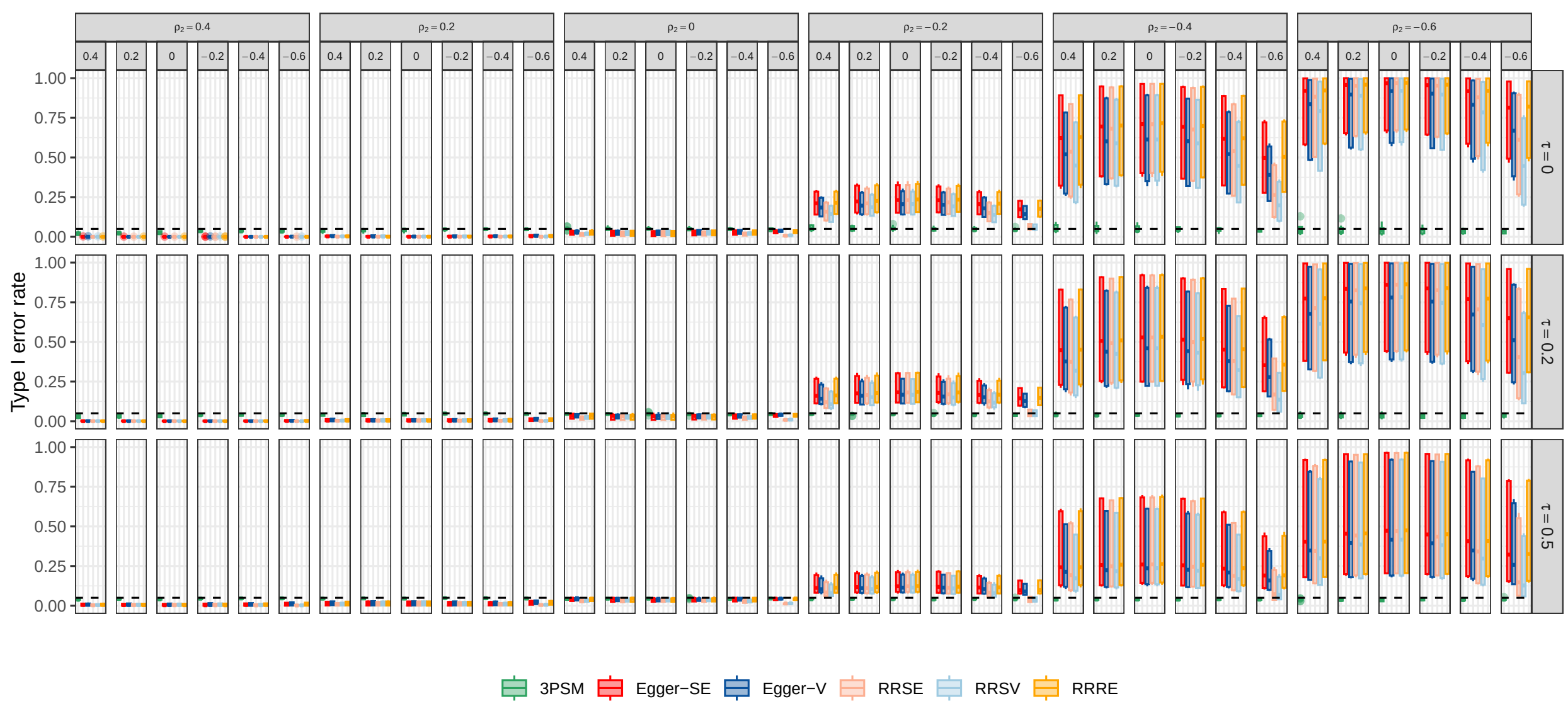

Figure S9: Type I error rates when the binary covariate was modeled in the sampels of $k = 80$.

S1.2.2 Varying β

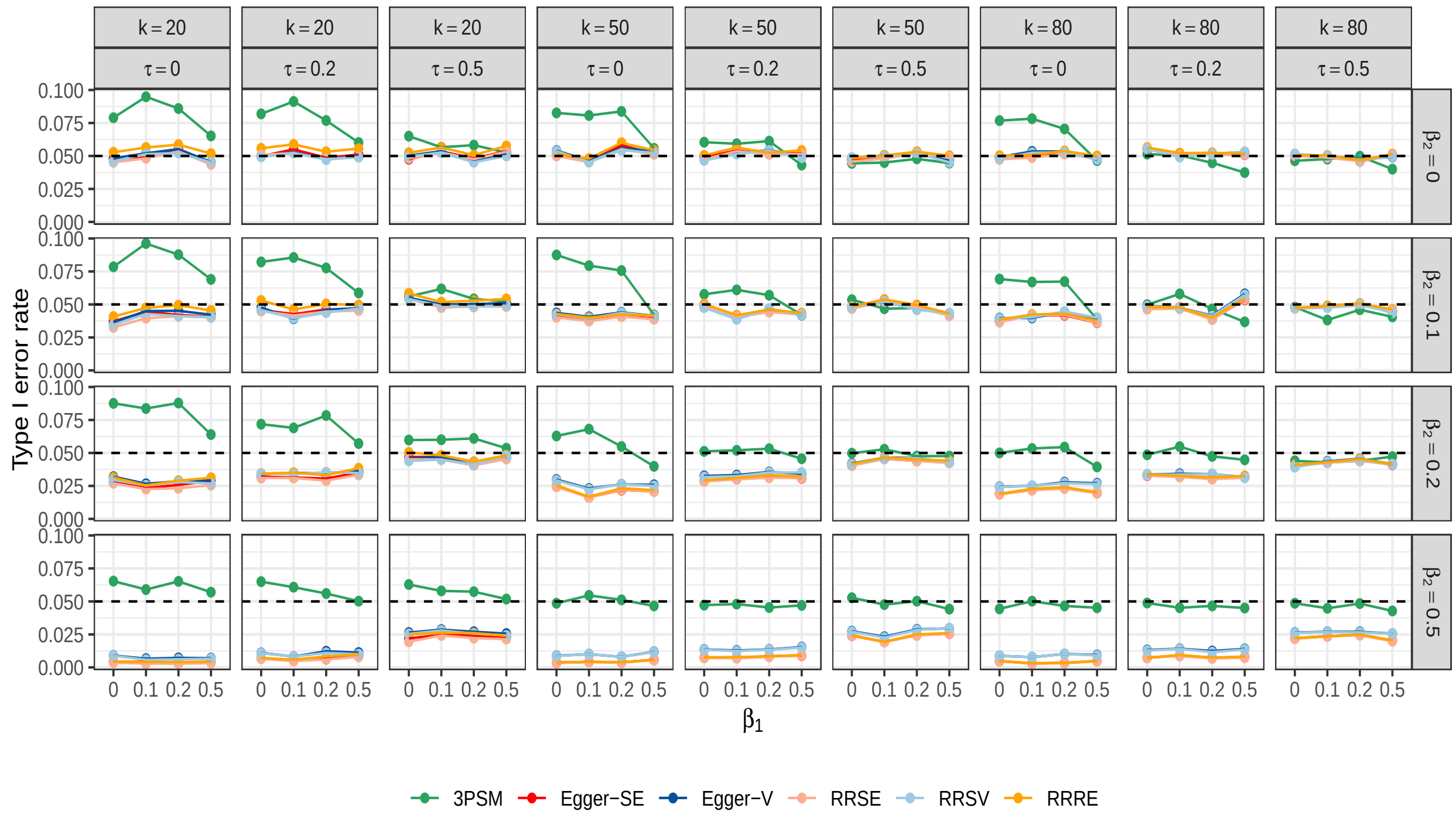

Figure S10: Type I error rates when the binary variable was modeled in the absence of confounding ($\rho_1 = \rho_2 = 0$).

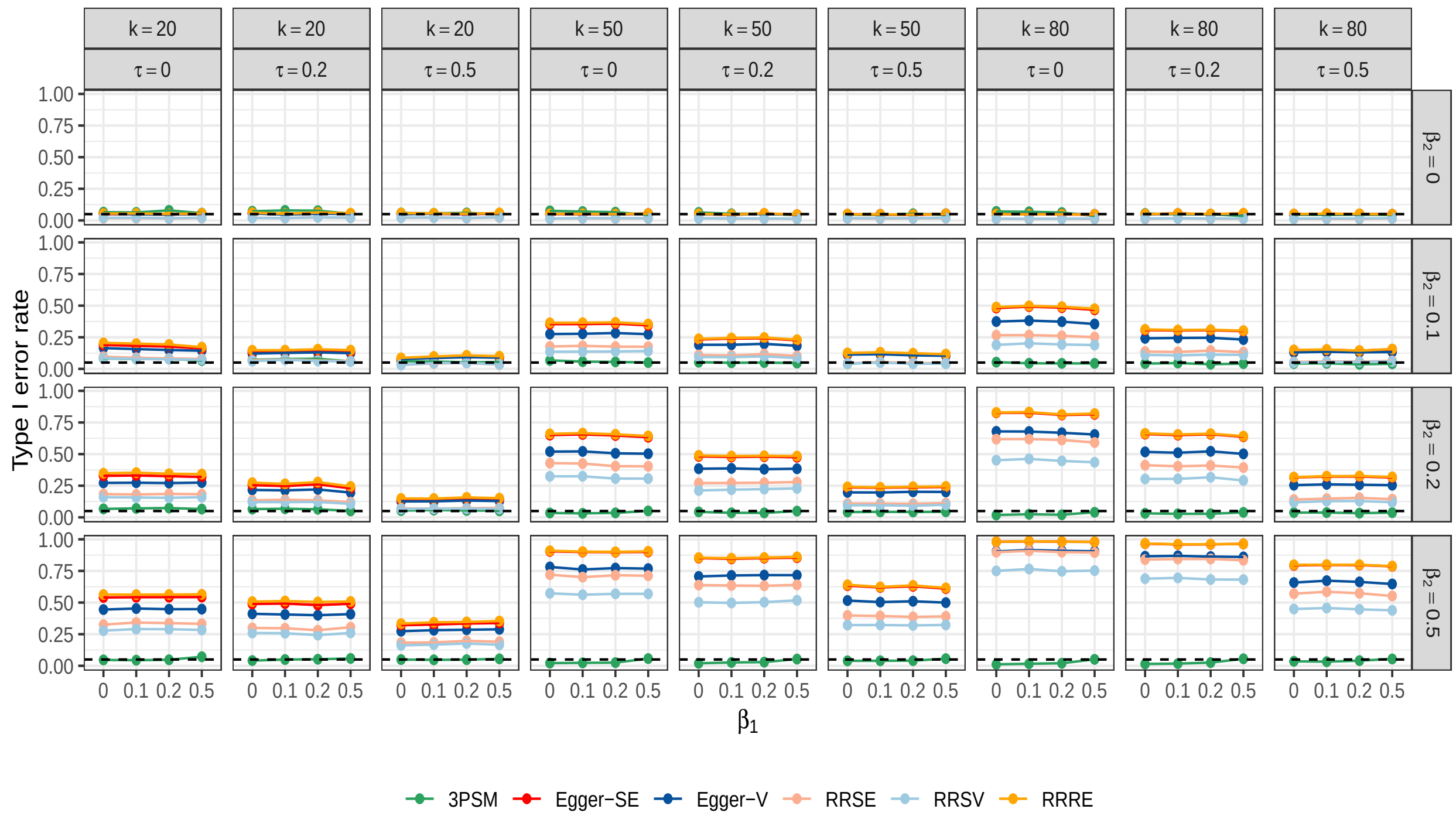

Figure S11: Type I error rates when the binary variable was modeled with strong confounding ($\rho_1 = \rho_2 = -0.6$).

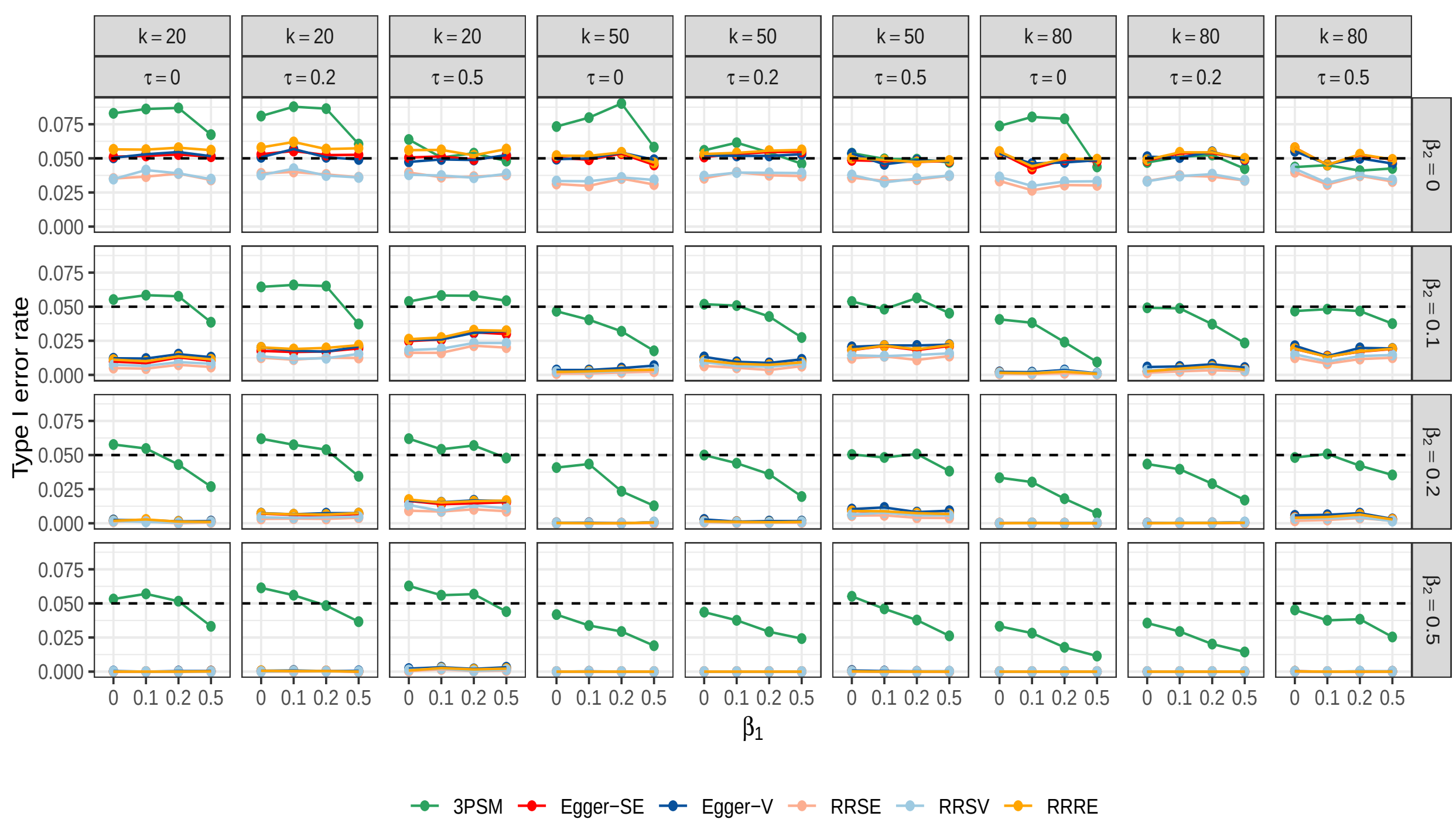

Figure S12: Type I error rates when the binary variable was modeled with mixed confounding ($\rho_1 = \rho_2 = 0.4$).

S1.3 Incorporating both covariates when $\beta_1 \neq 0$ and $\beta_2 \neq 0$

S1.3.1 Varying k

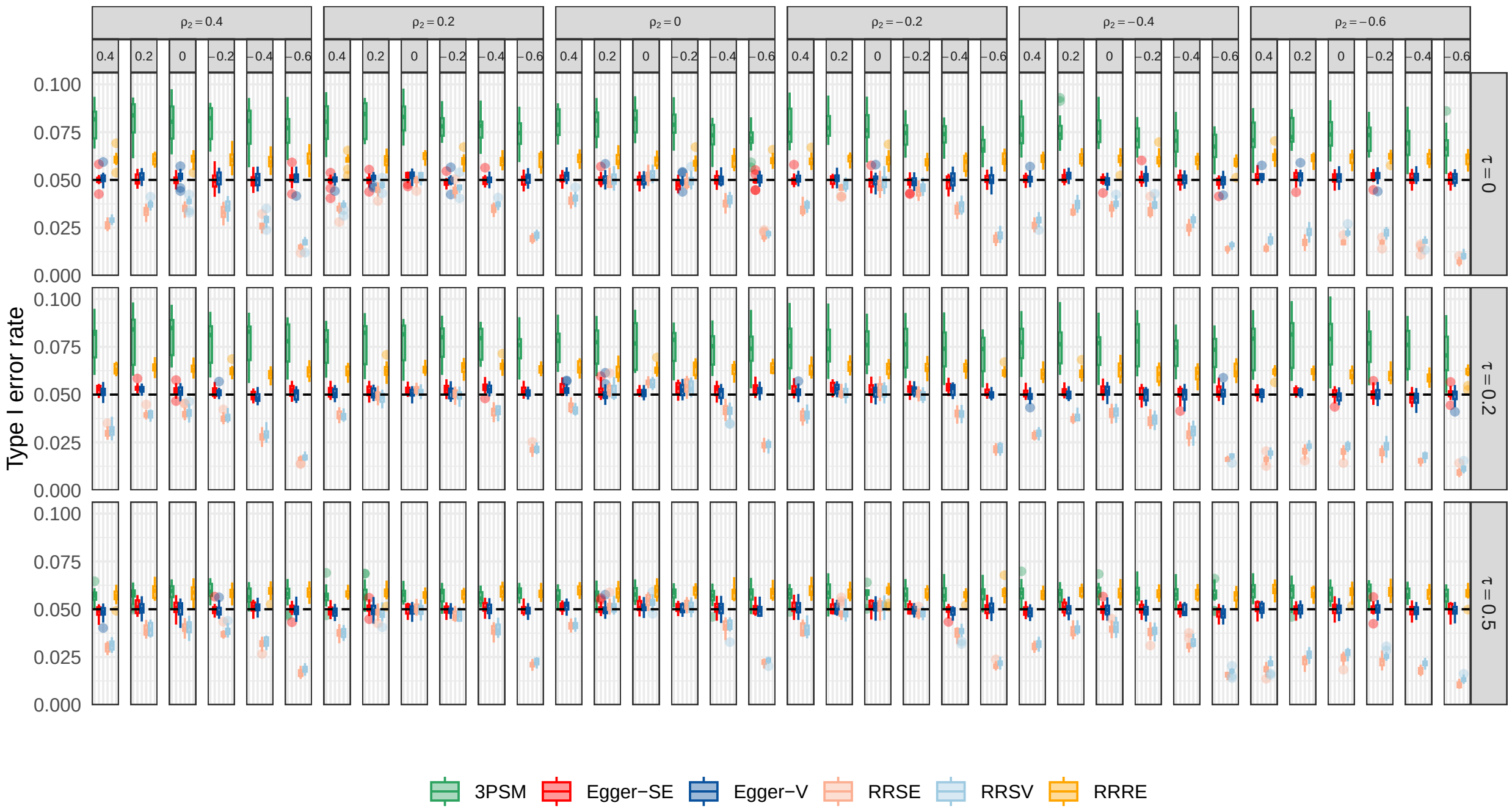

Figure S13: Type I error rates when both covariates were modeled in the sampels of $k = 20$.

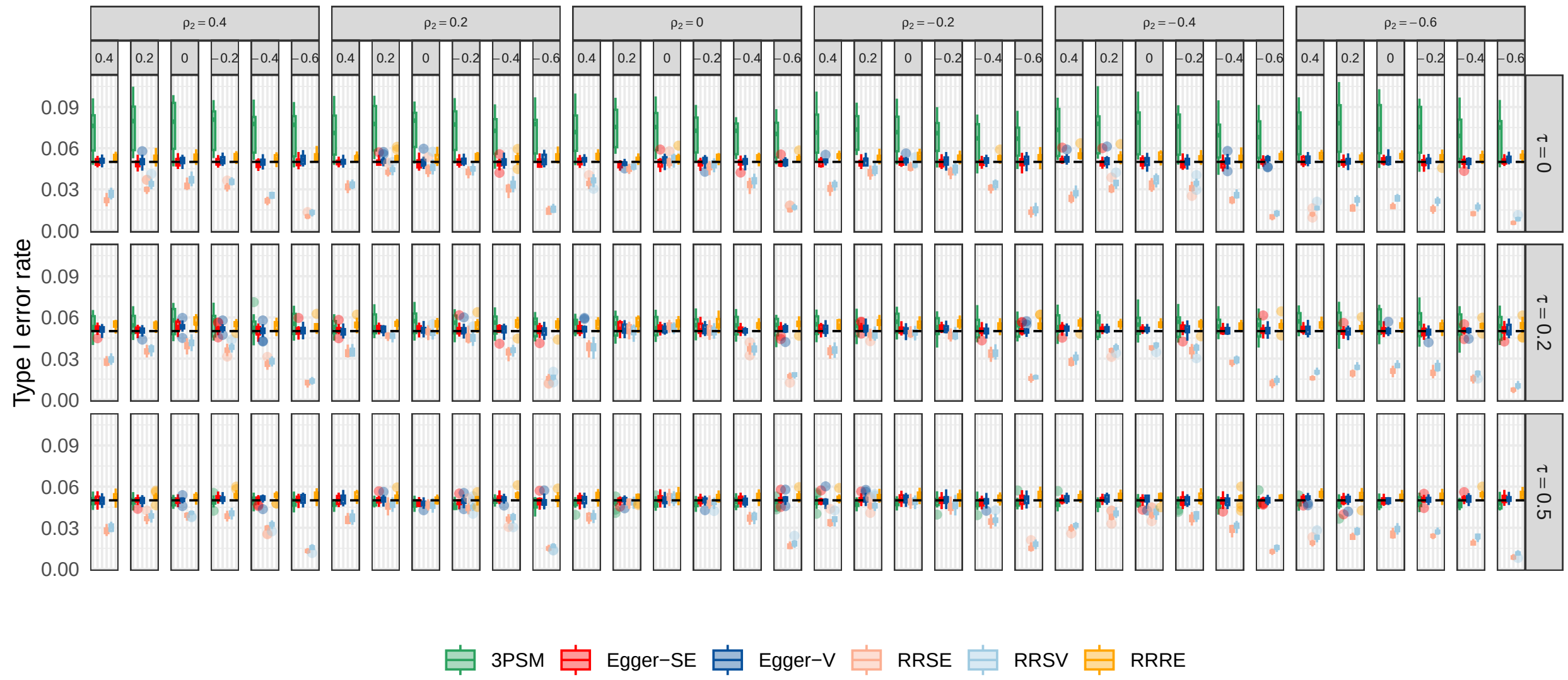

Figure S14: Type I error rates when both covariates were modeled in the sampels of $k = 50$.

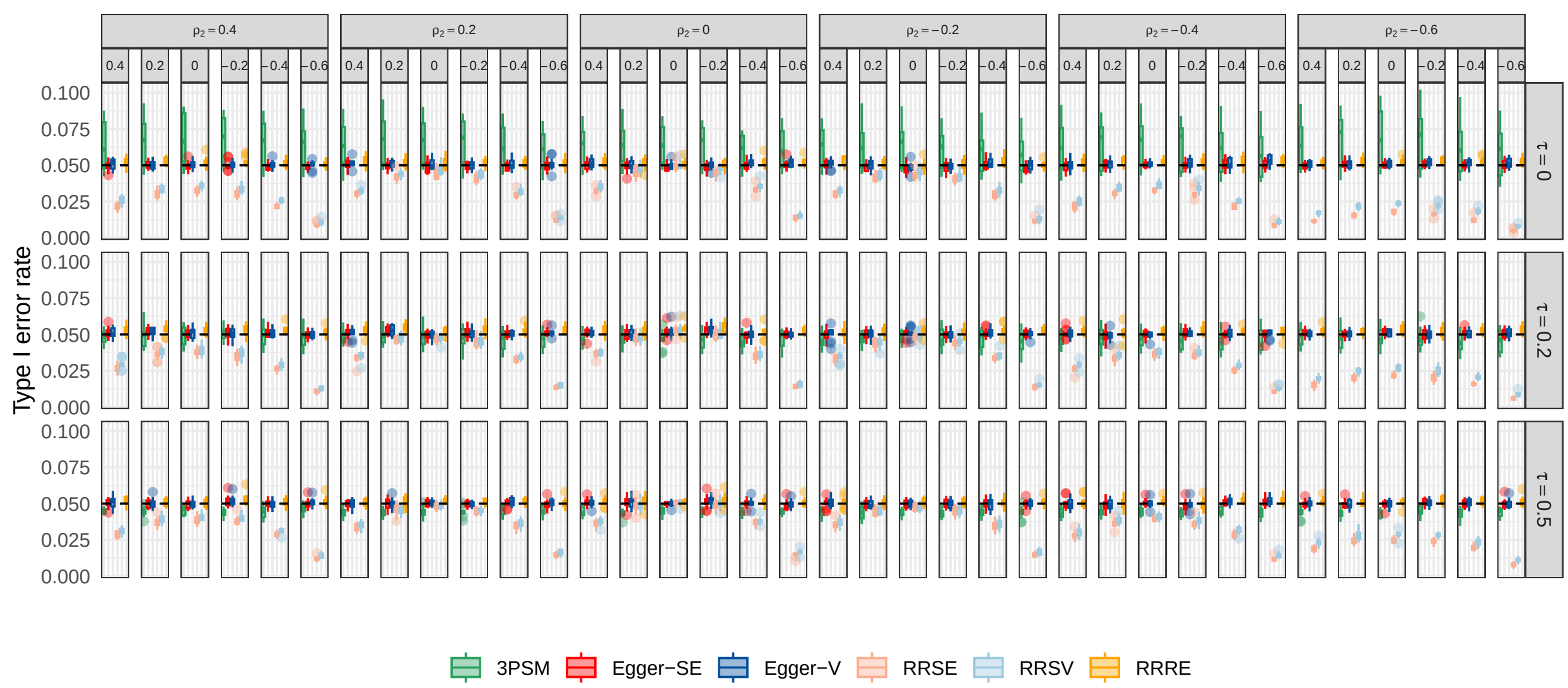

Figure S15: Type I error rates when both covariates were modeled in the sampels of $k = 80$.

S1.3.2 Varying β

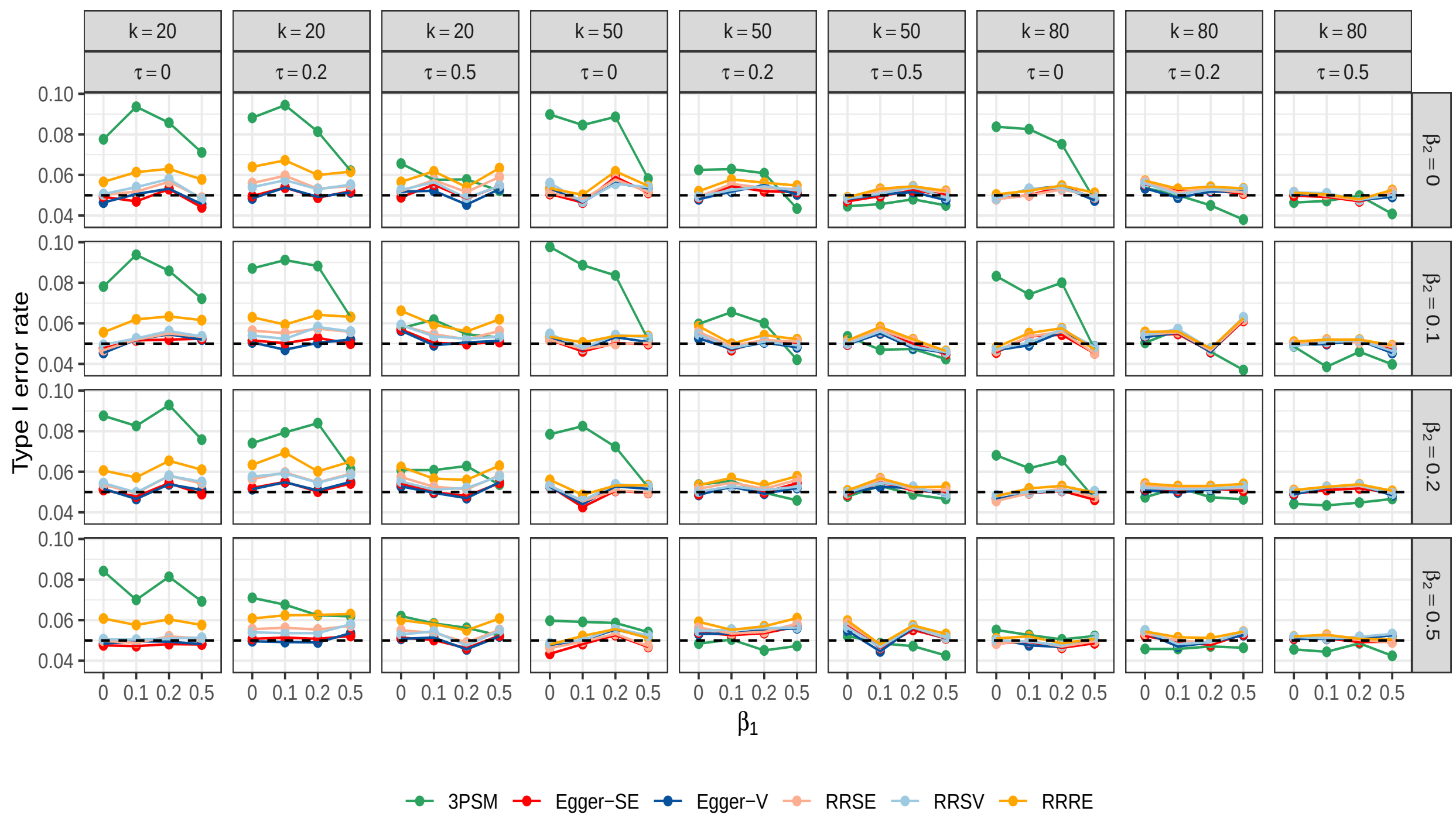

Figure S16: Type I error rates when both covariates were modeled in the absence of confounding ($\rho_1 = \rho_2 = 0$).

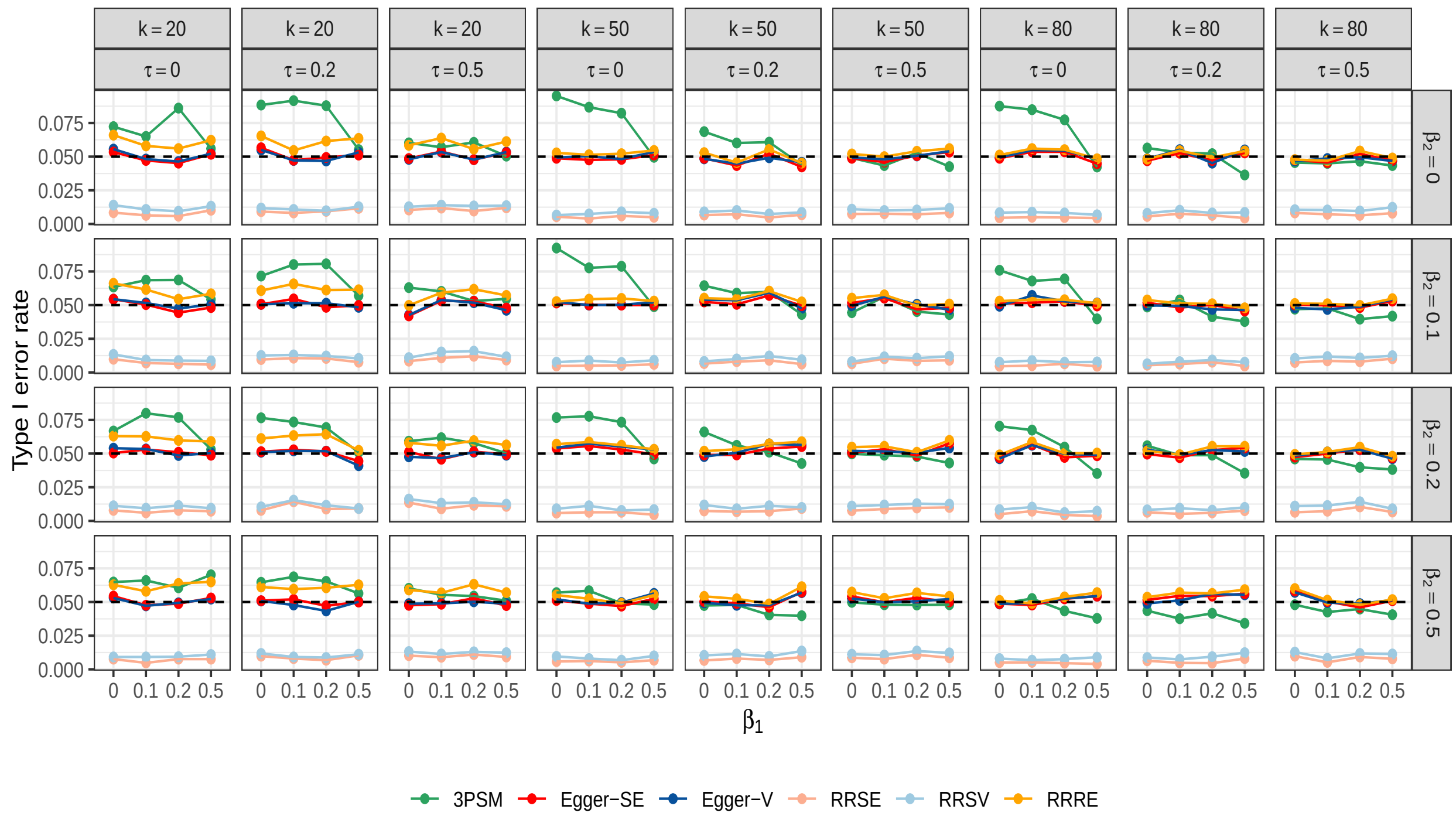

Figure S17: Type I error rates when both covariates were modeled with strong confounding ($\rho_1 = \rho_2 = -0.6$).

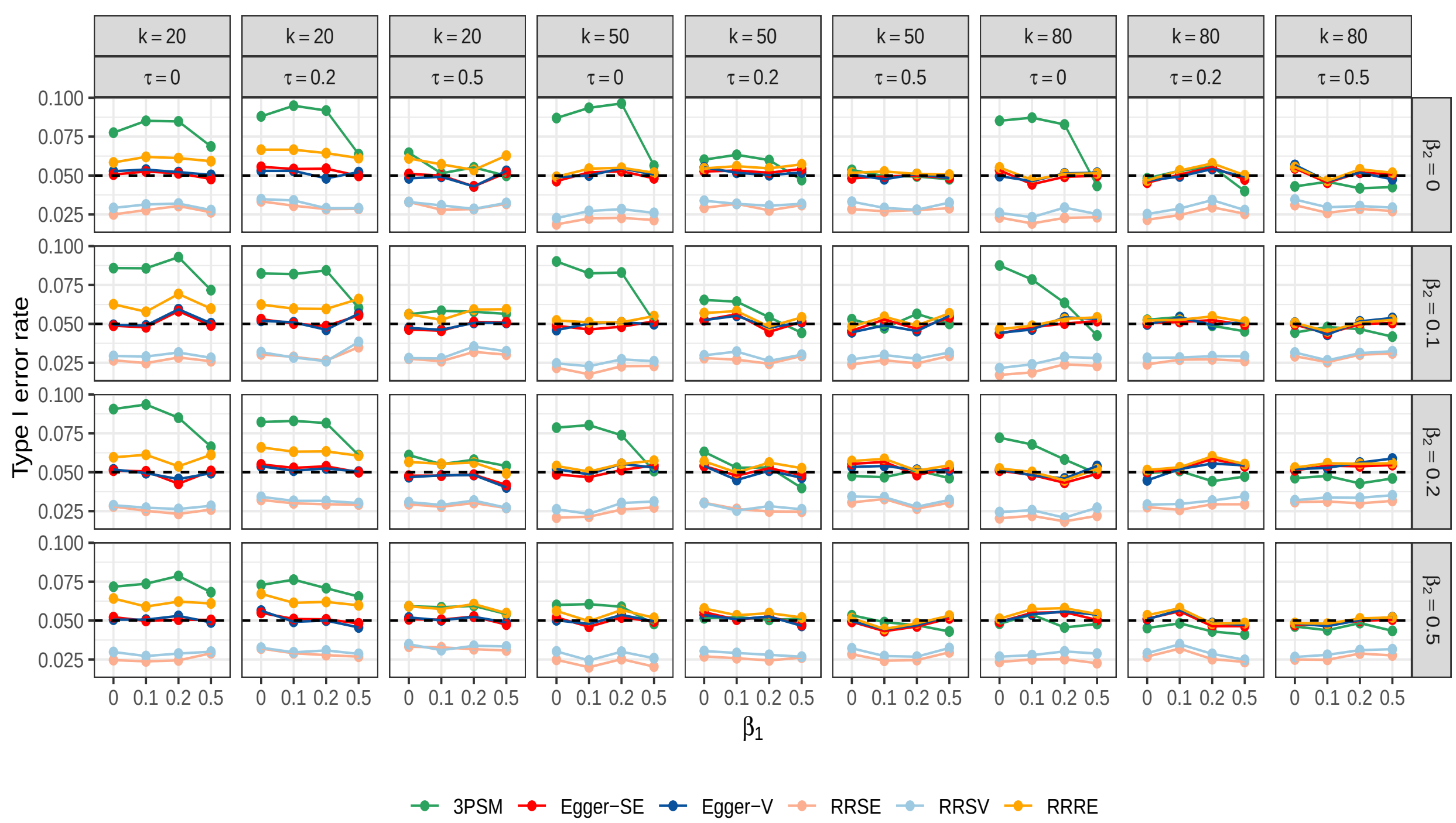

Figure S18: Type I error rates when both covariates were modeled with mixed confounding ($\rho_1 = \rho_2 = 0.4$).

S2 Type I error rates of each method

S2.1 3PSM

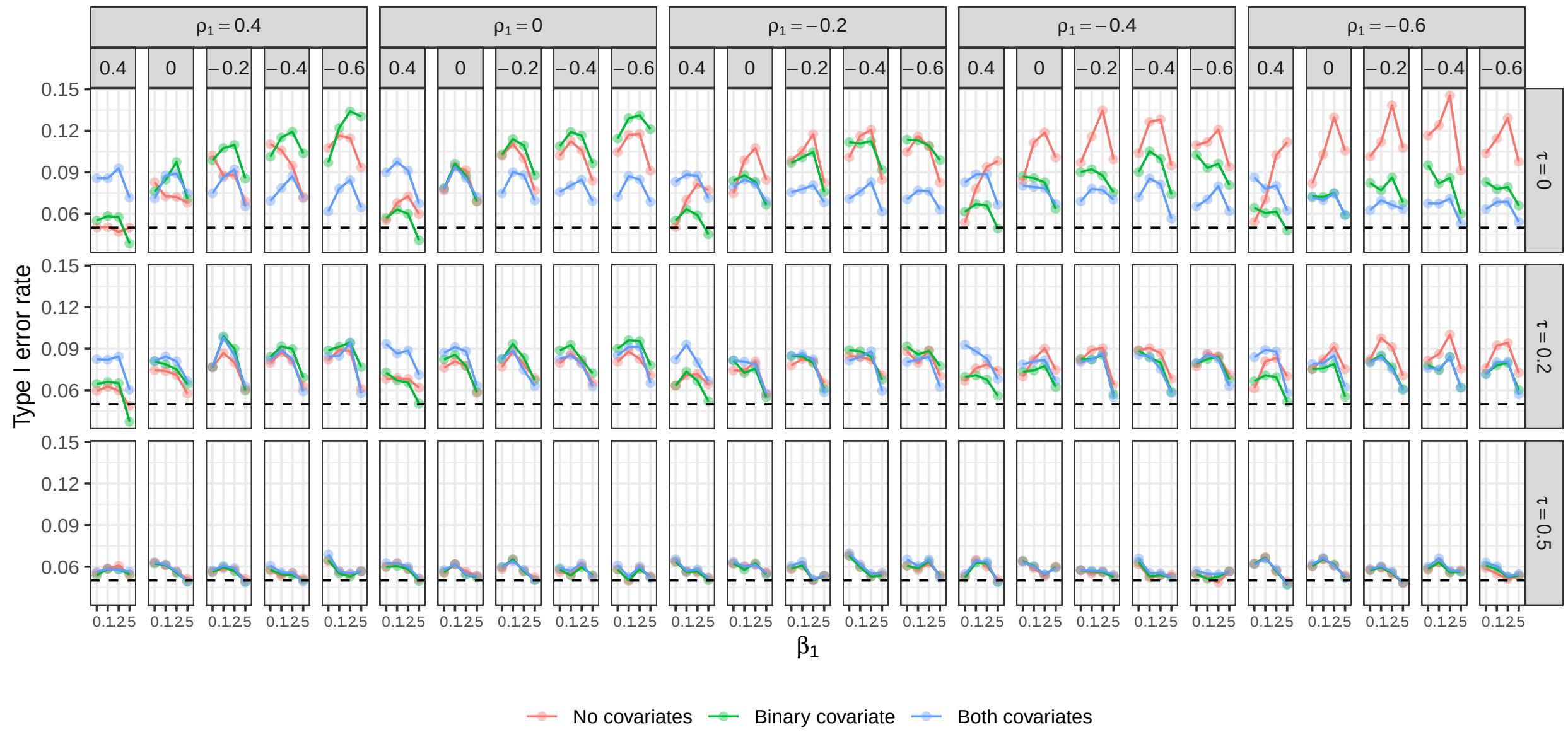

Figure S19: Type I error rates of 3SPM likelihood ratio test for $k = 20$ and $\beta_2 = 0.1$.

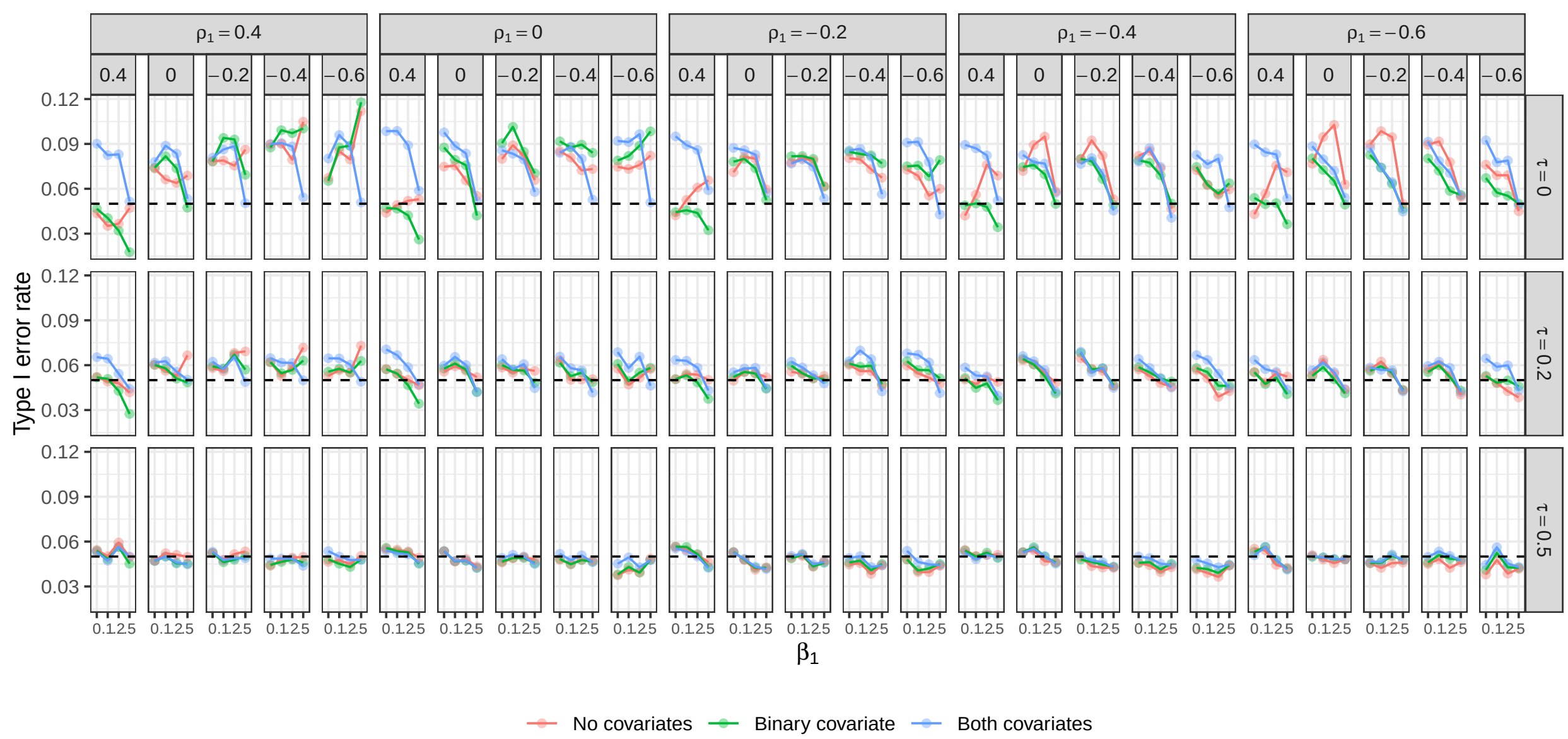

Figure S20: Type I error rates of 3SPM likelihood ratio test for $k = 50$ and $\beta_2 = 0.1$.

S2.2 Egger-SE

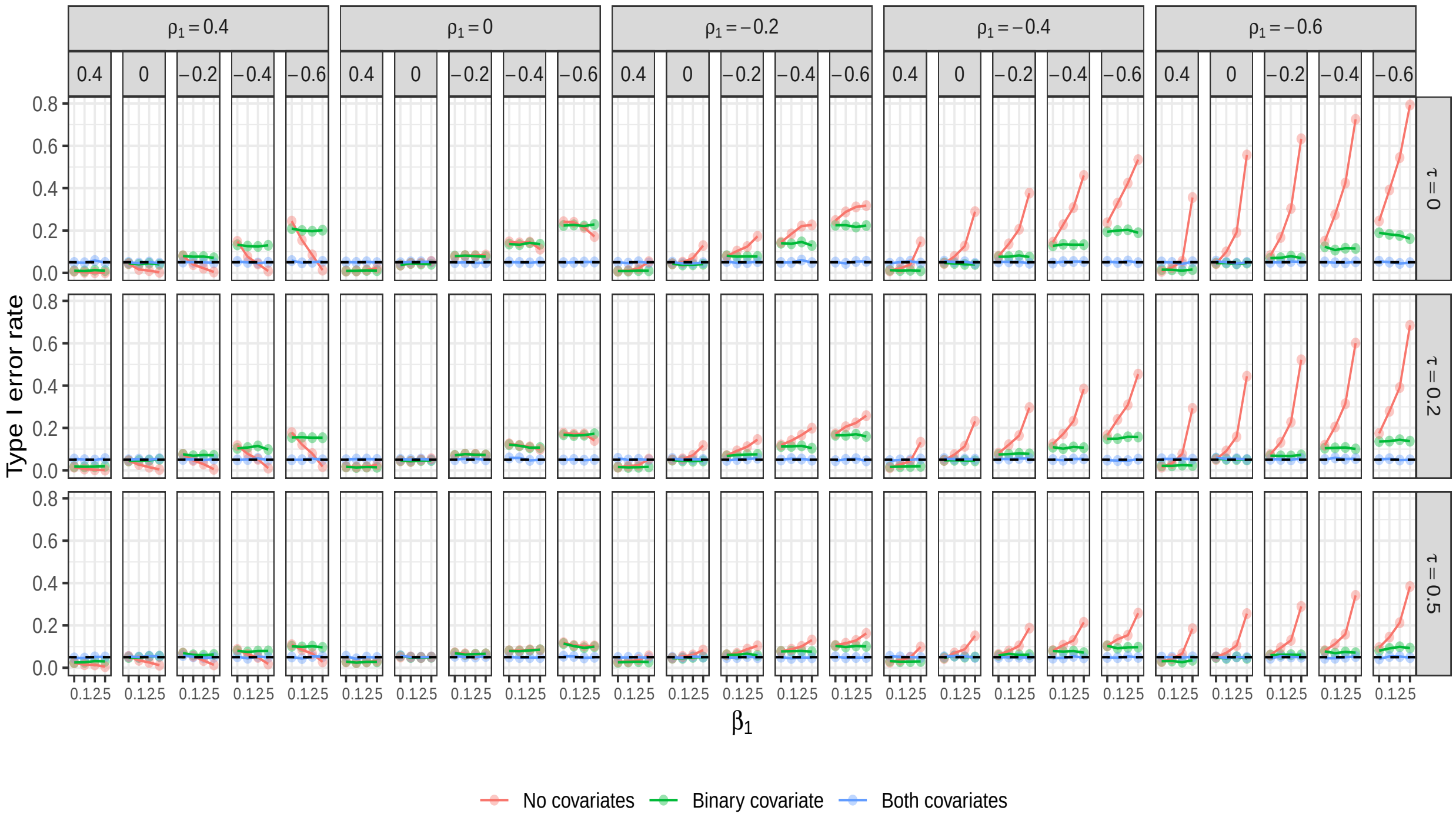

Figure S21: Type I error rates of Egger-SE for $k = 20$ and $\beta_2 = 0.1$.

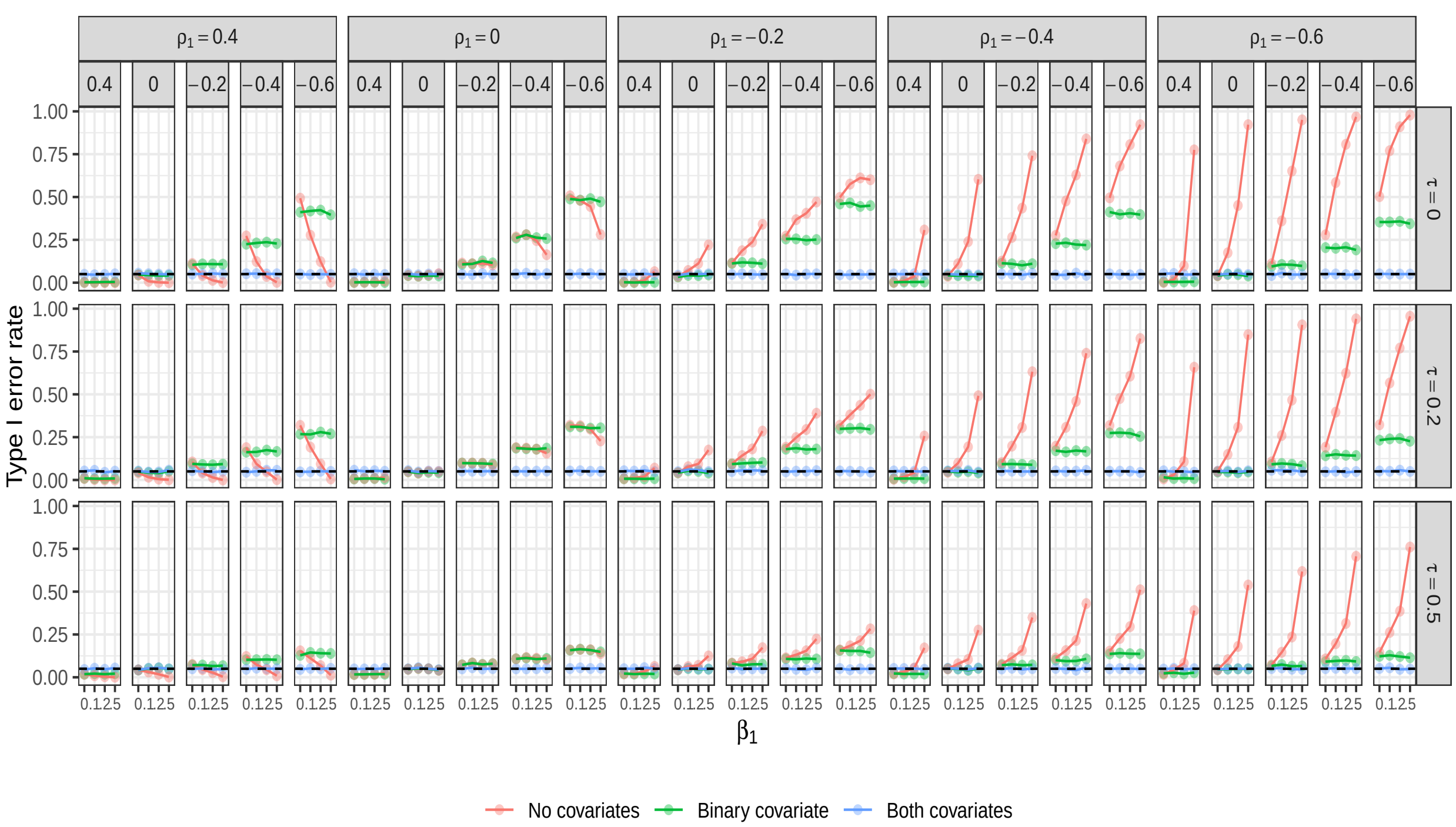

Figure S22: Type I error rates of Egger-SE for $k = 50$ and $\beta_2 = 0.1$.

S2.3 Egger-V

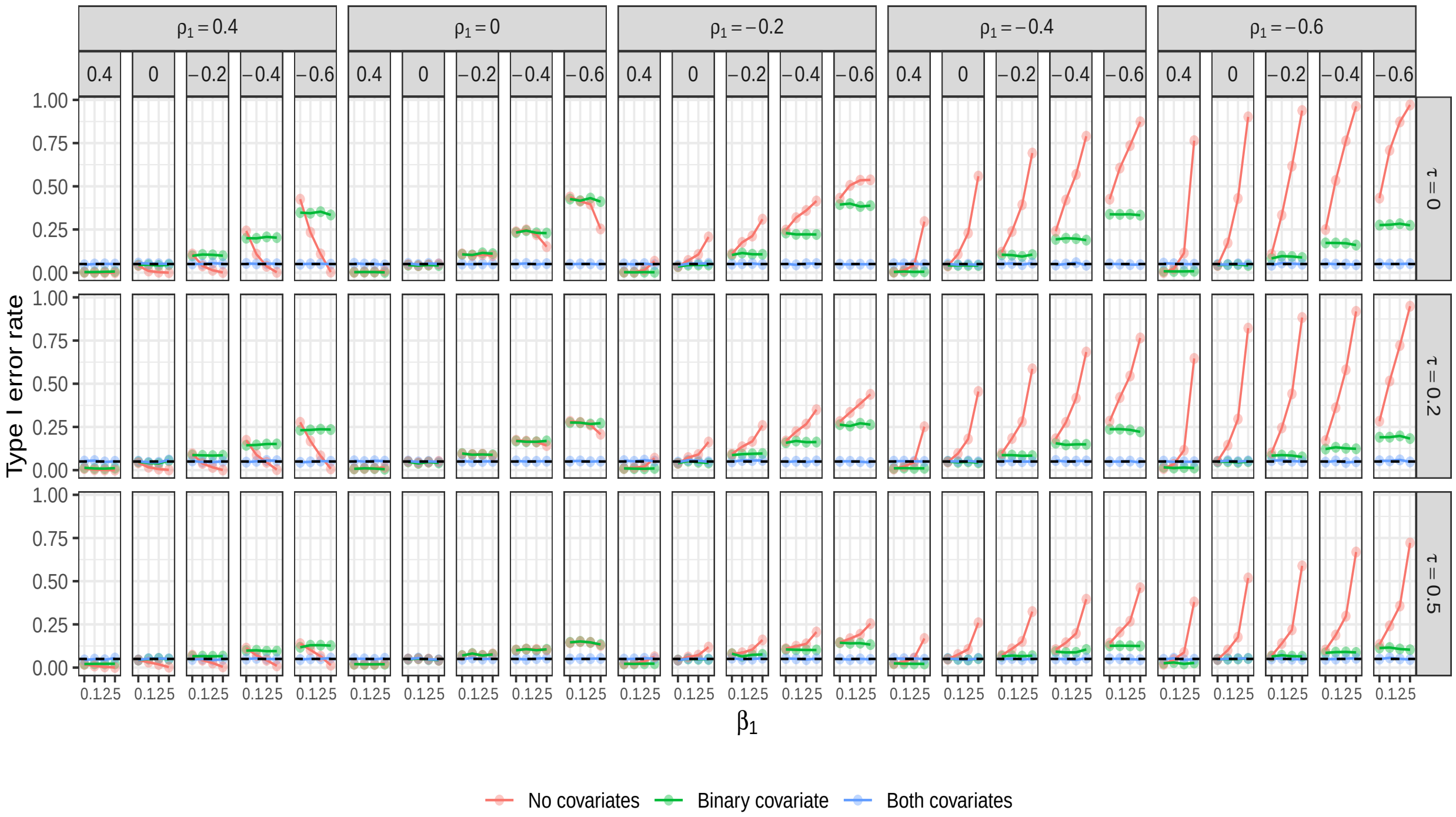

Figure S23: Type I error rates of Egger-V for $k = 20$ and $\beta_2 = 0.1$.

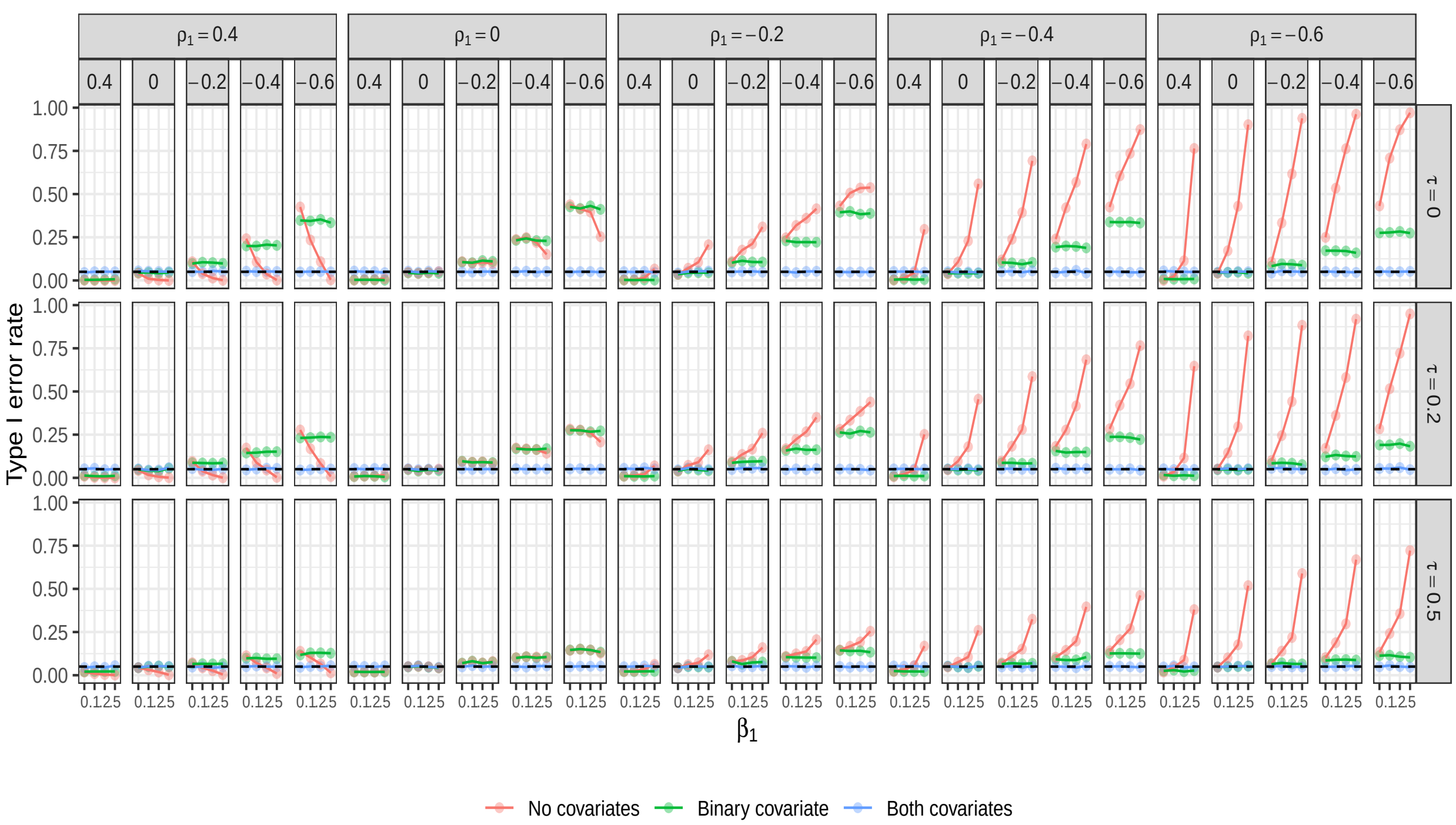

Figure S24: Type I error rates of Egger-V for $k = 50$ and $\beta_2 = 0.1$.

S2.4 RRSE

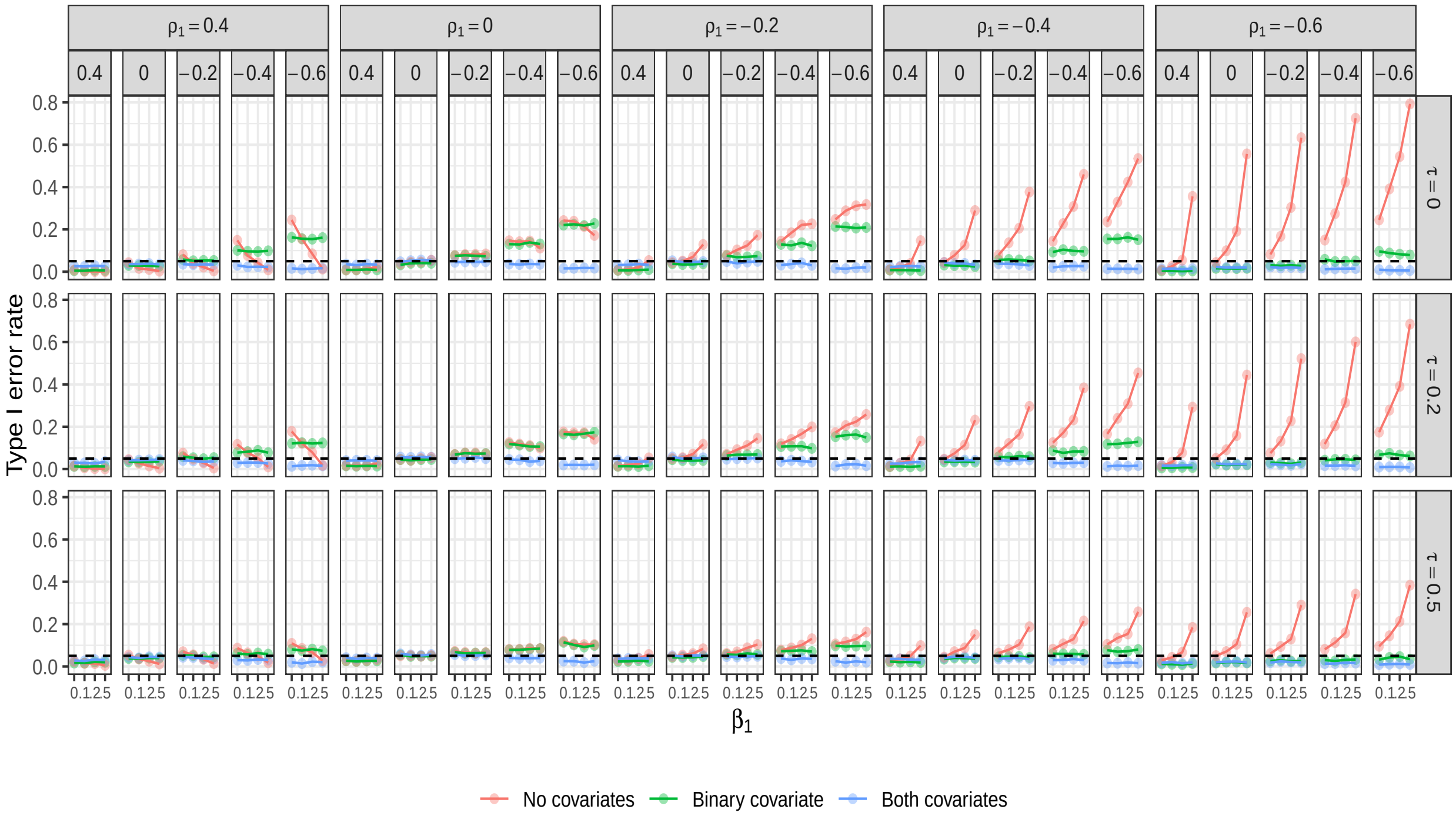

Figure S25: Type I error rates of RRSE for $k = 20$ and $\beta_2 = 0.1$.

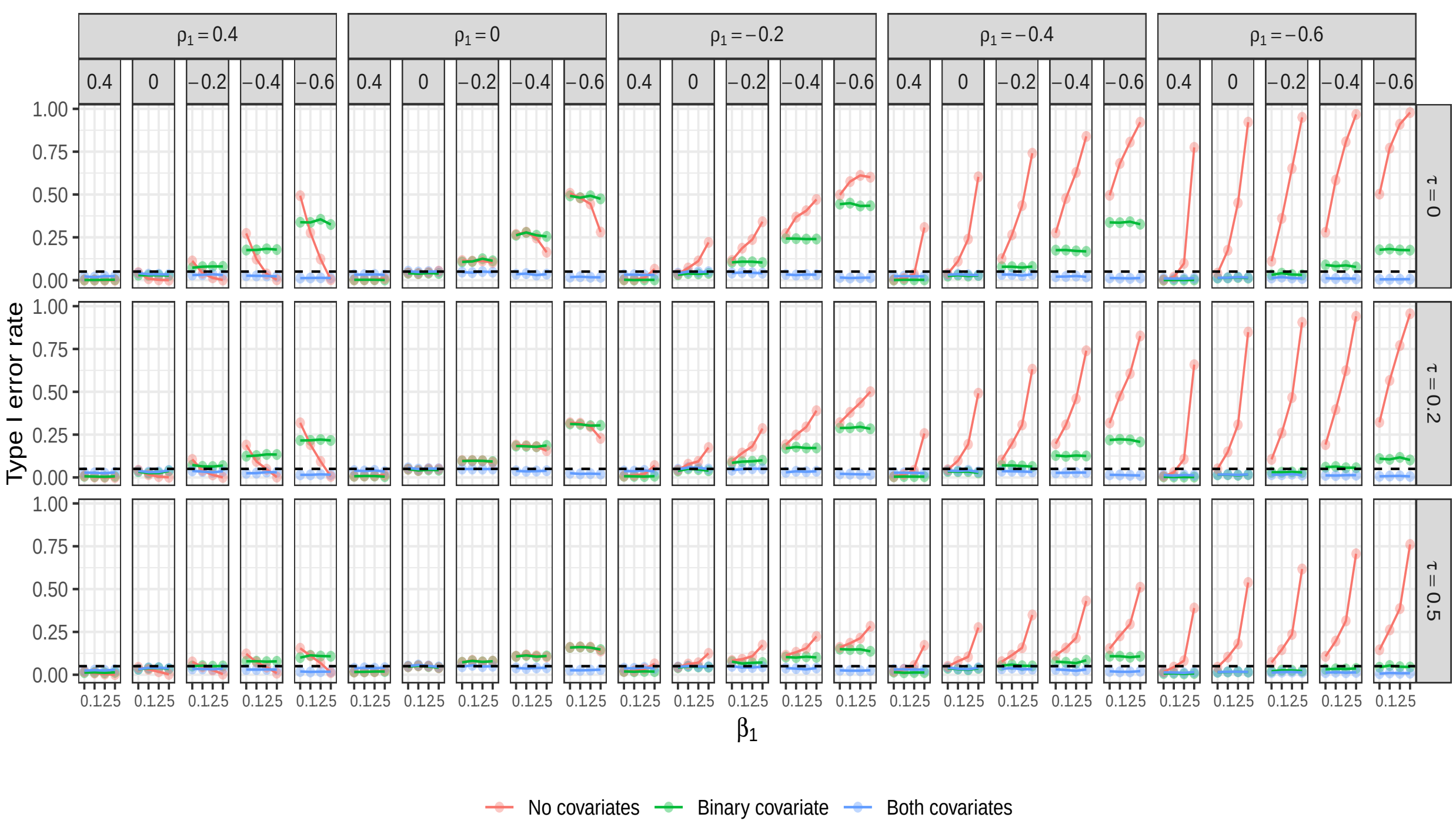

Figure S26: Type I error rates of RRSE for $k = 50$ and $\beta_2 = 0.1$.

S2.5 RRRE

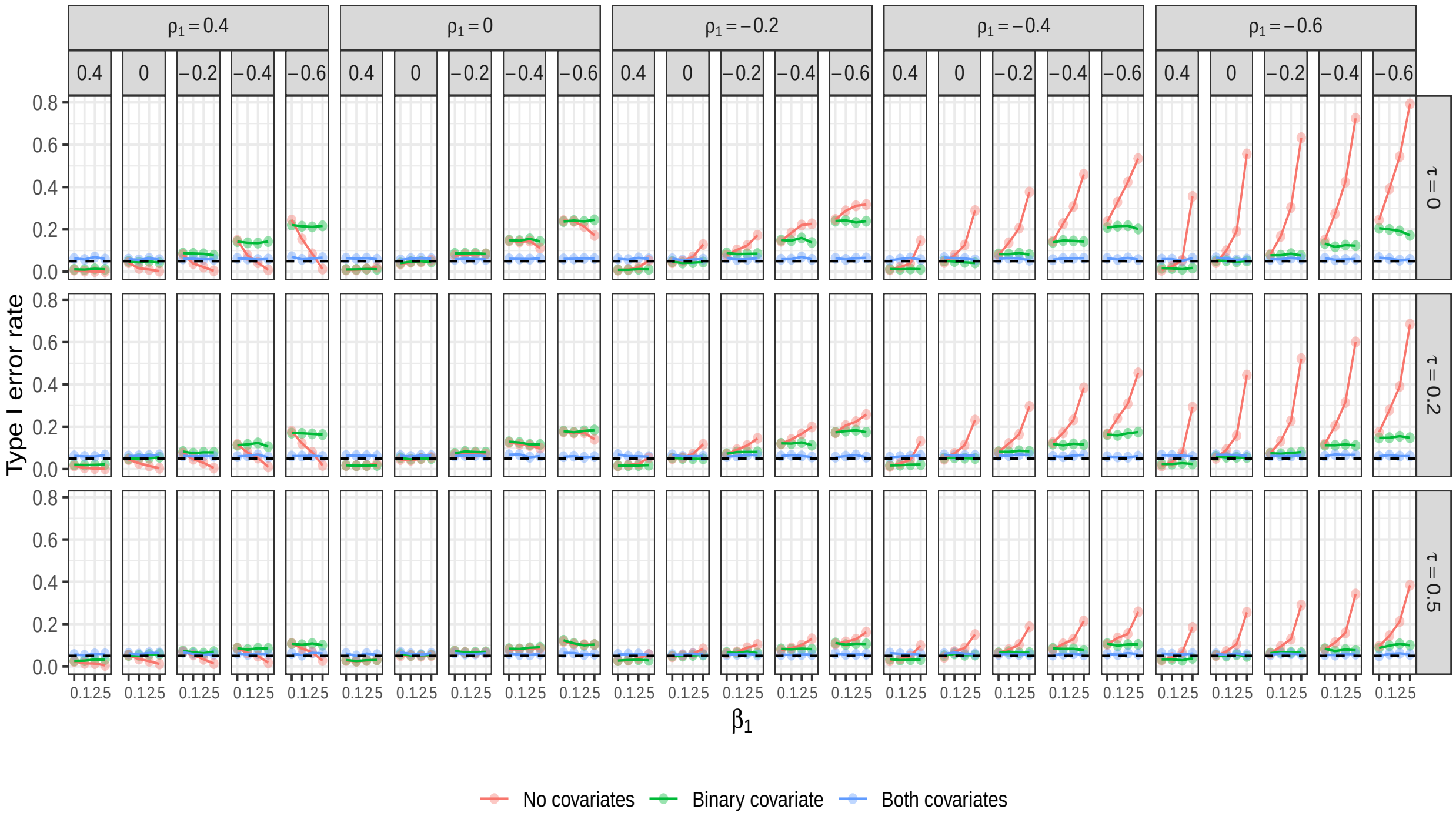

Figure S27: Type I error rates of RRRE for $k = 20$ and $\beta_2 = 0.1$.

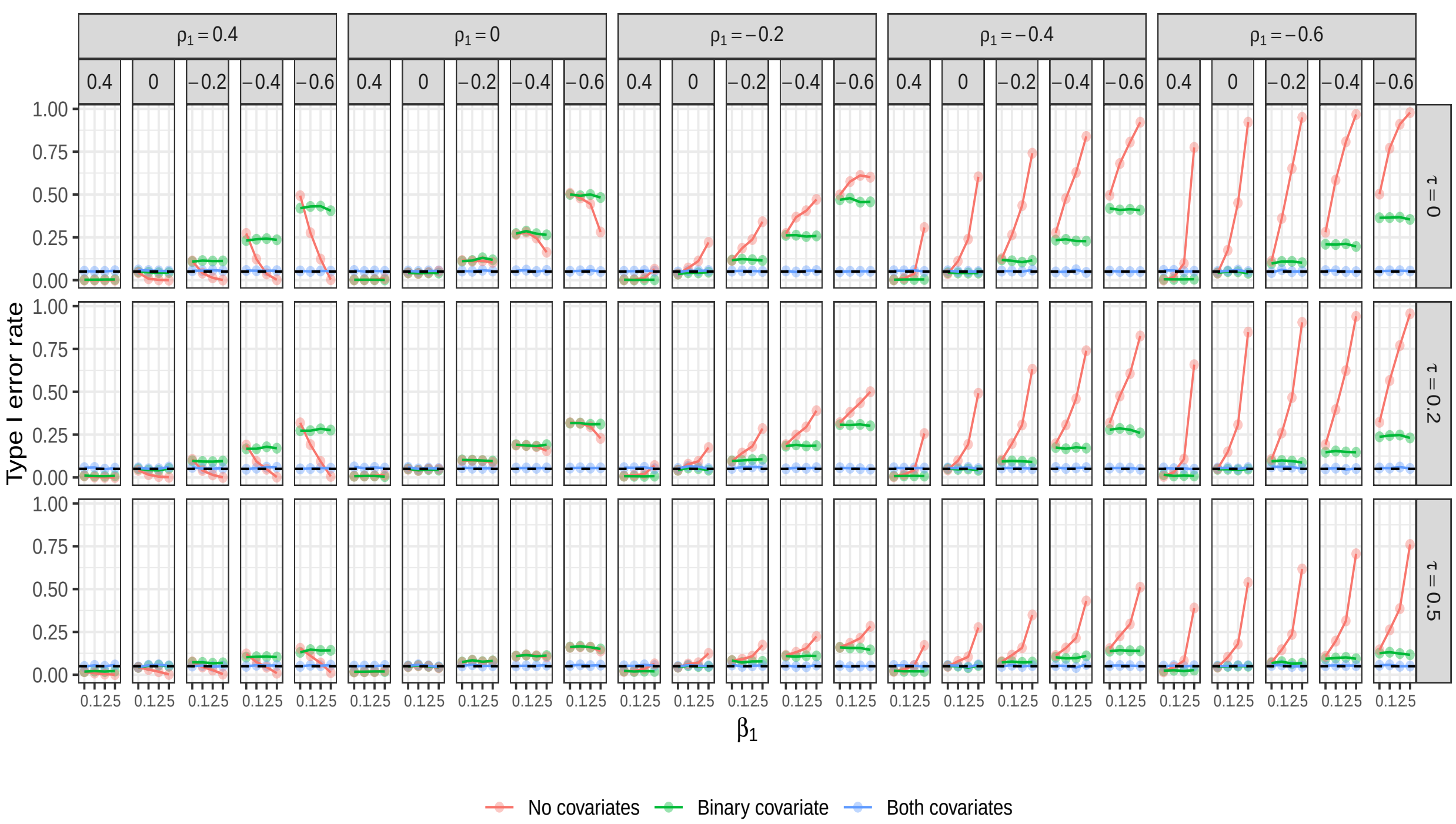

Figure S28: Type I error rates of RRRE for $k = 50$ and $\beta_2 = 0.1$.

S3 Power when both covariates were modeled

S3.1 $\beta_2 = 0.1$

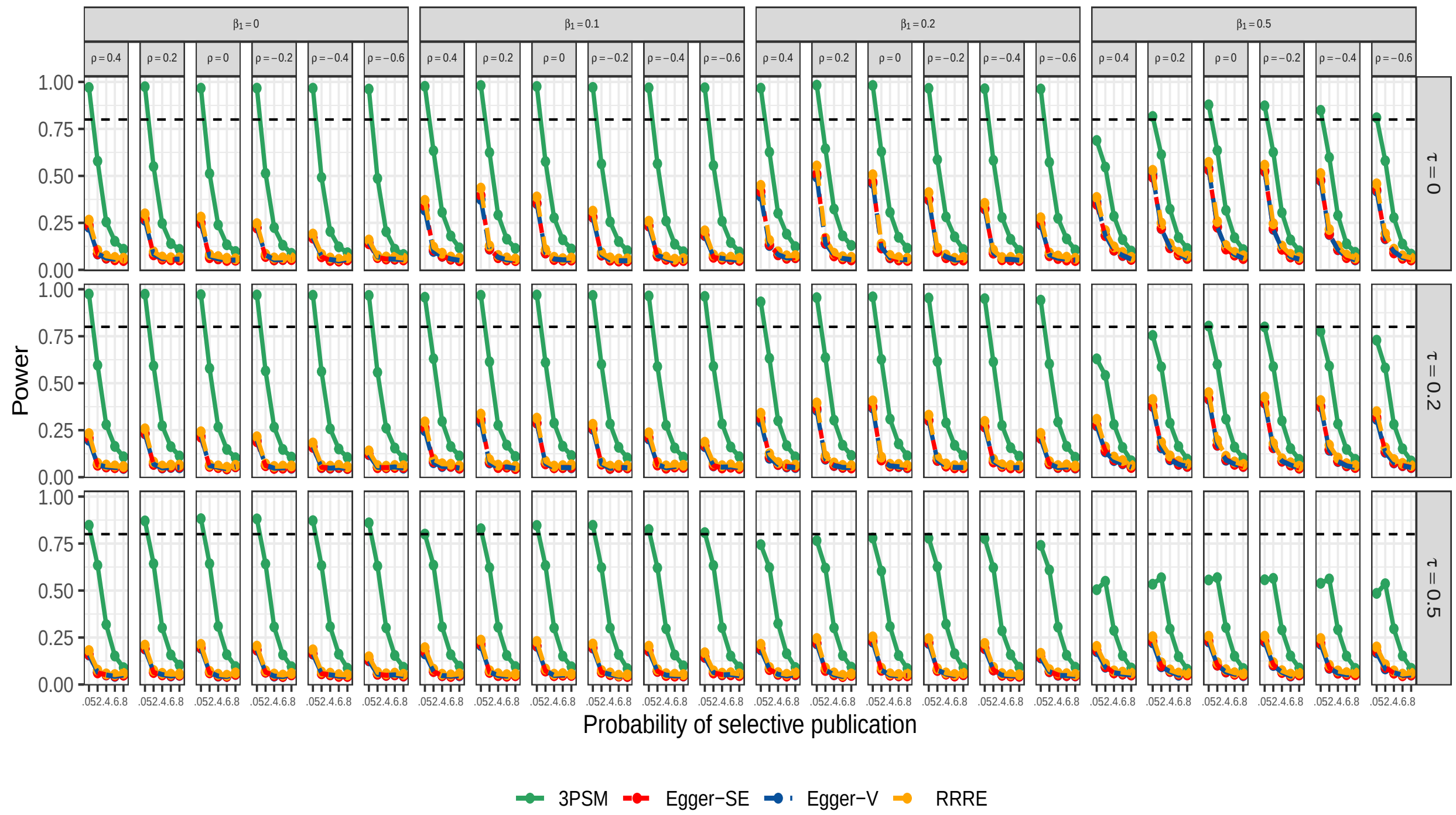

Figure S29: Power for meta-analyses with $k = 20$ studies.

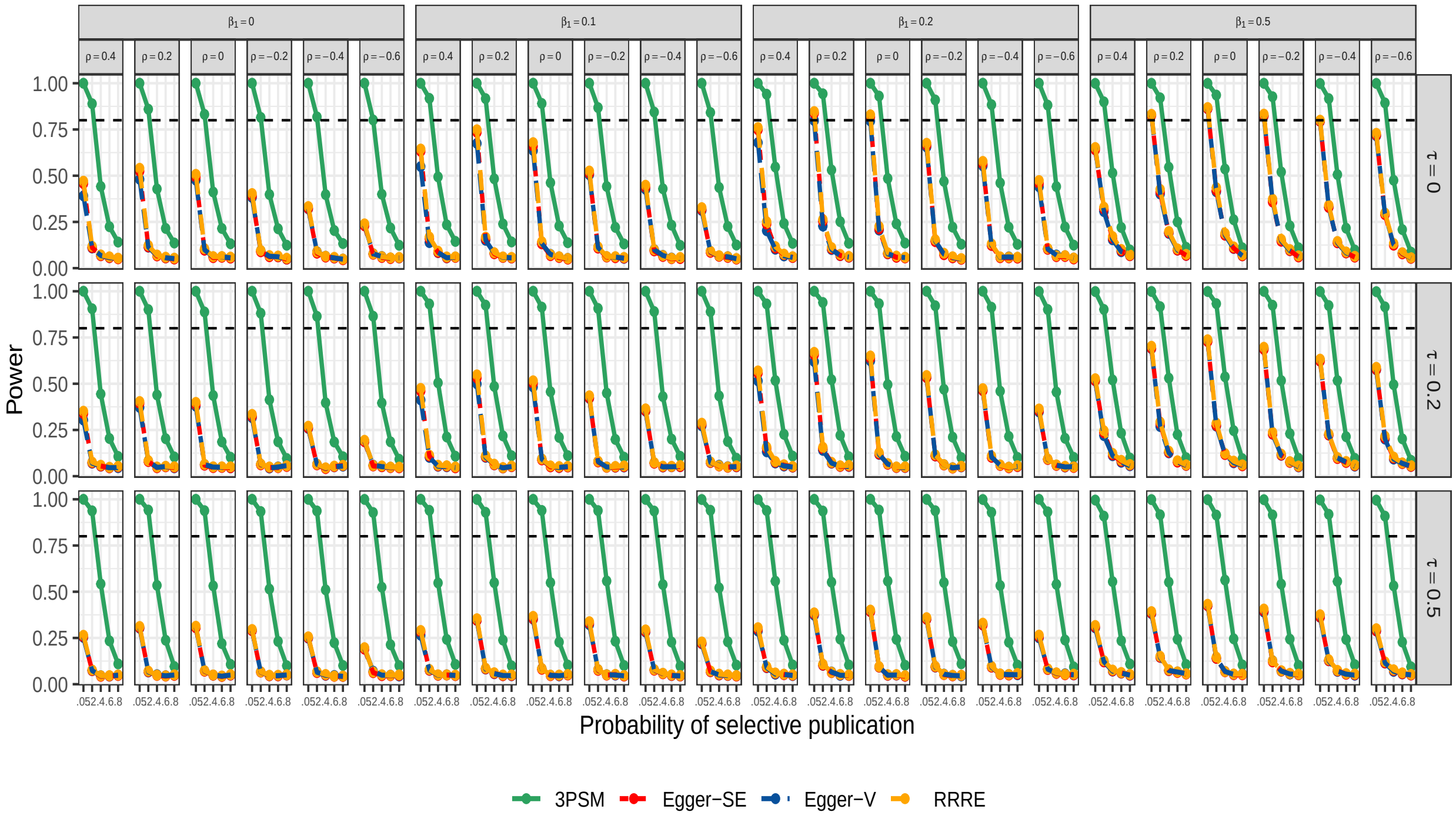

Figure S30: Power for meta-analyses with $k = 50$ studies.

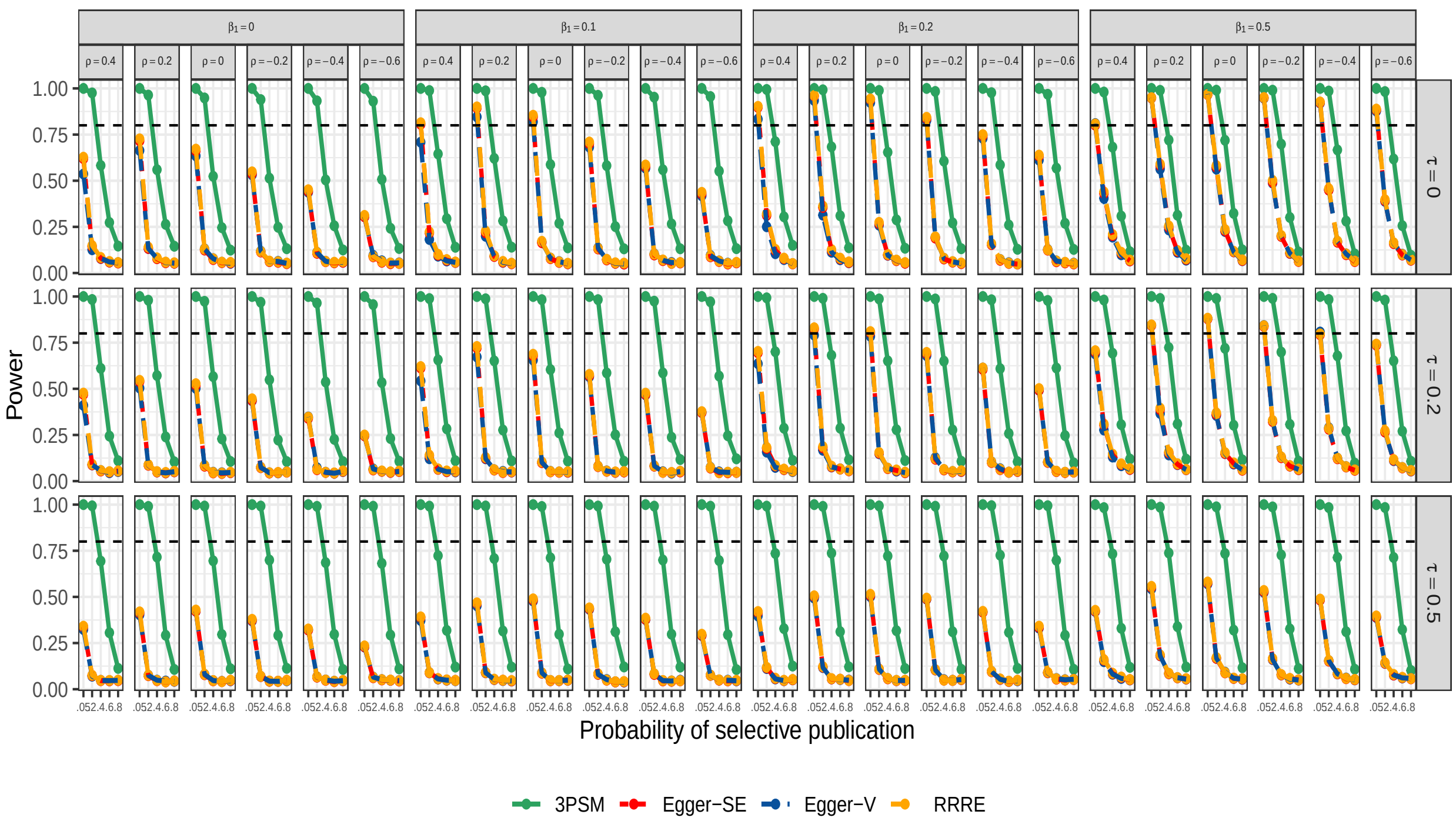

Figure S31: Power for meta-analyses with $k = 80$ studies.

S3.2 $\beta_2 = 0.2$

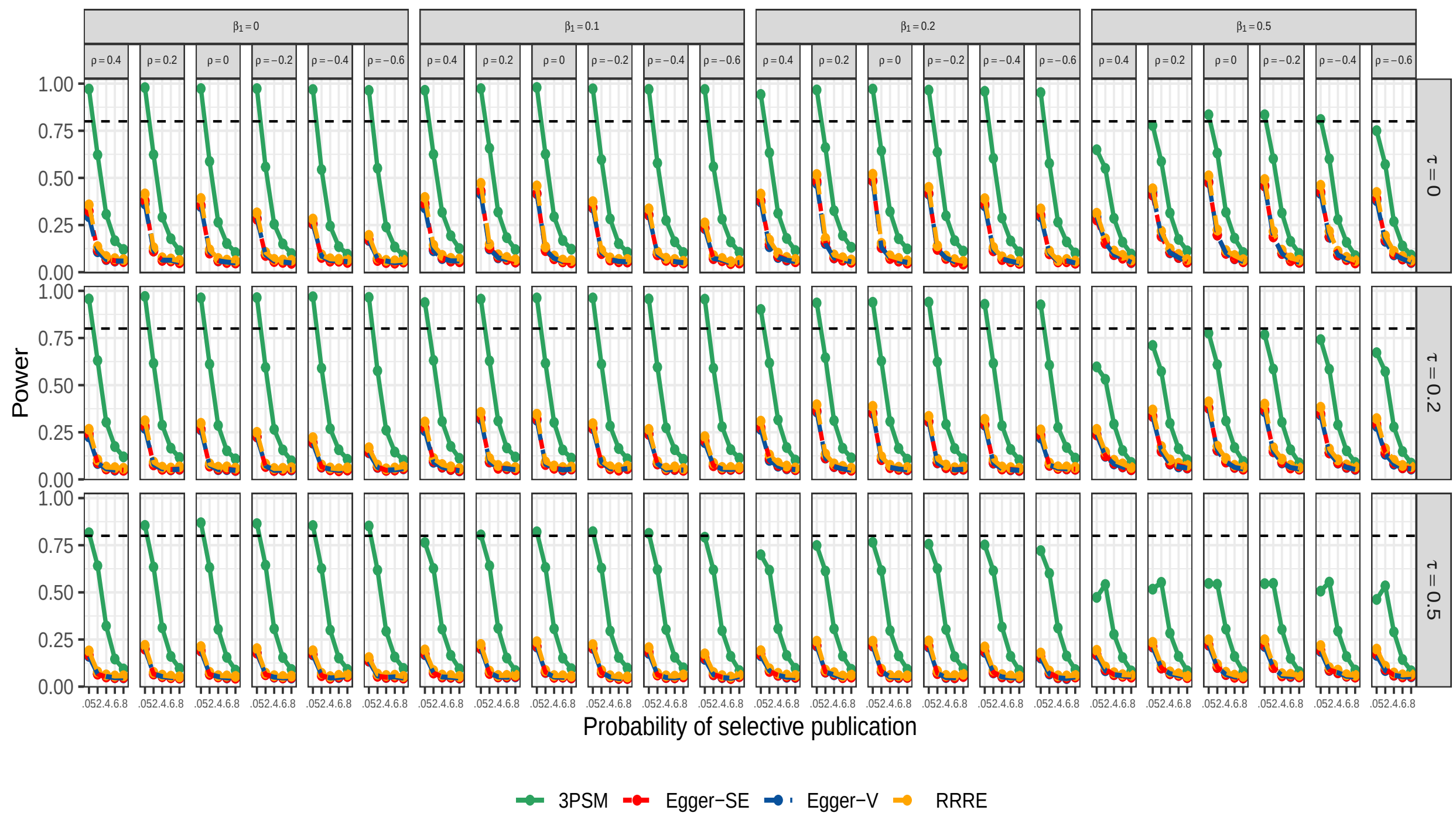

Figure S32: Power for meta-analyses with $k = 20$ studies and $\beta_2 = 0.2$.

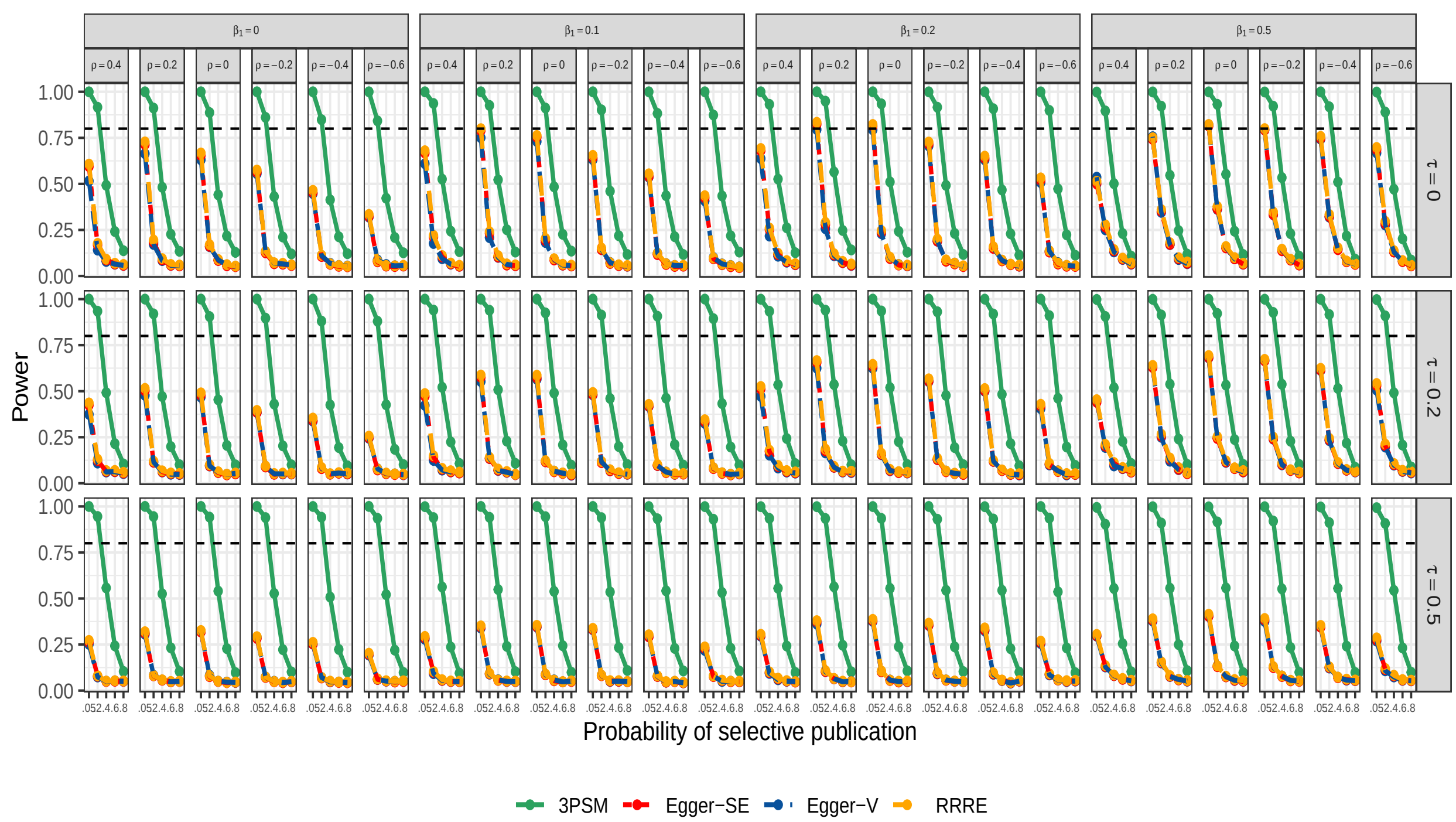

Figure S33: Power for meta-analyses with $k = 50$ studies and $\beta_2 = 0.2$.

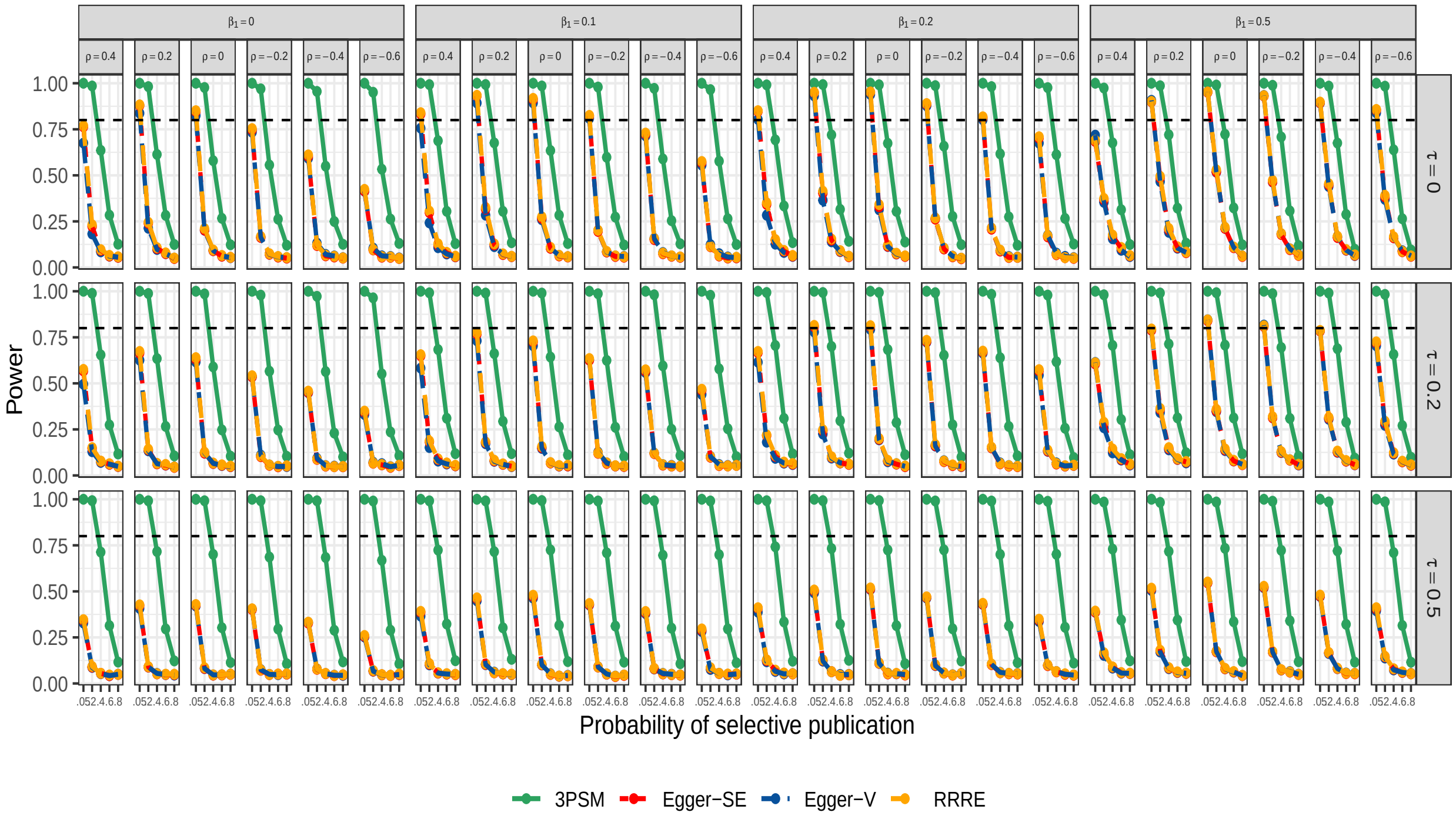

Figure S34: Power for meta-analyses with $k = 80$ studies and $\beta_2 = 0.2$.

S3.3 $\beta_2 = 0.5$

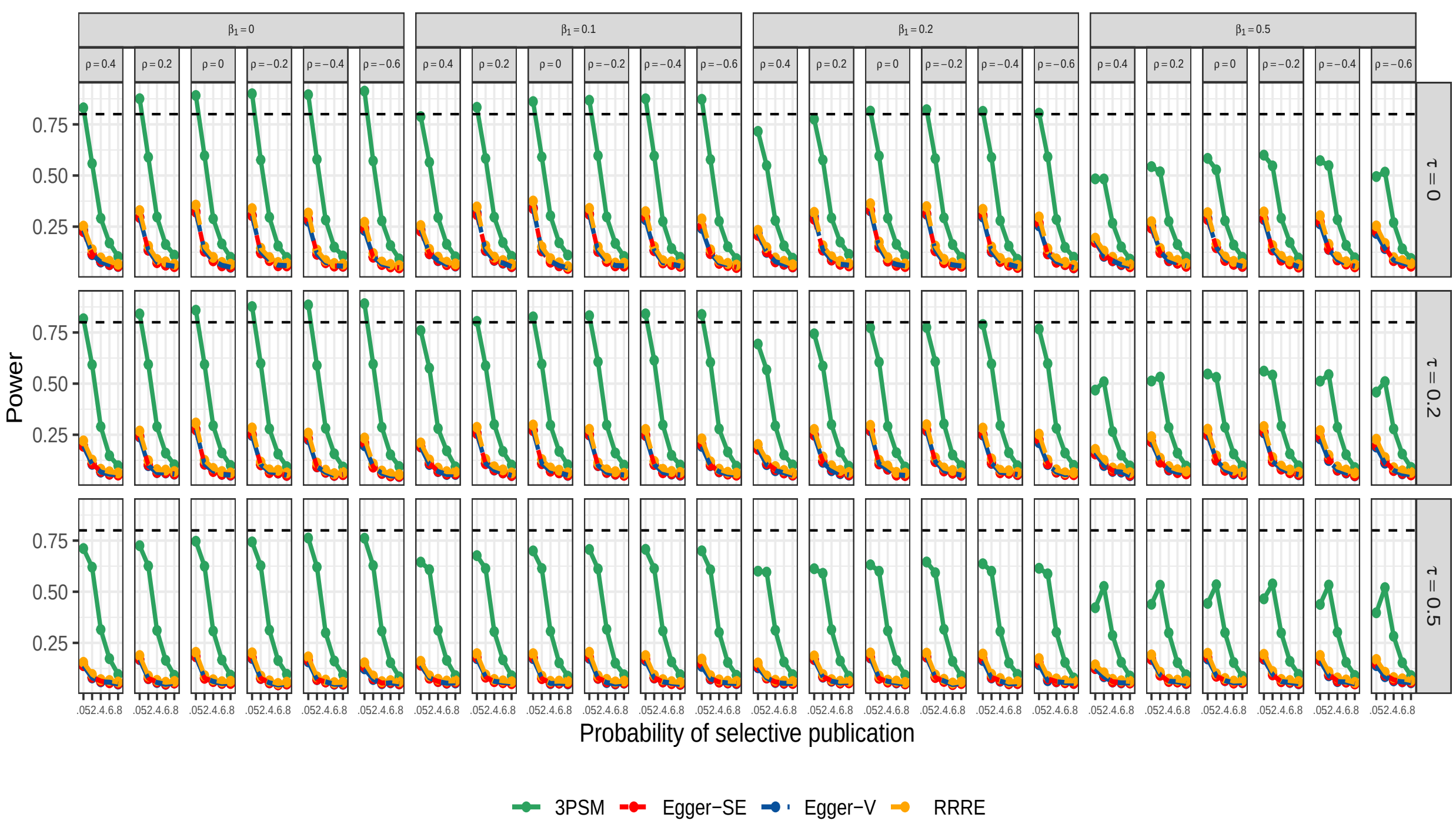

Figure S35: Power for meta-analyses with $k = 20$ studies and $\beta_2 = 0.5$.

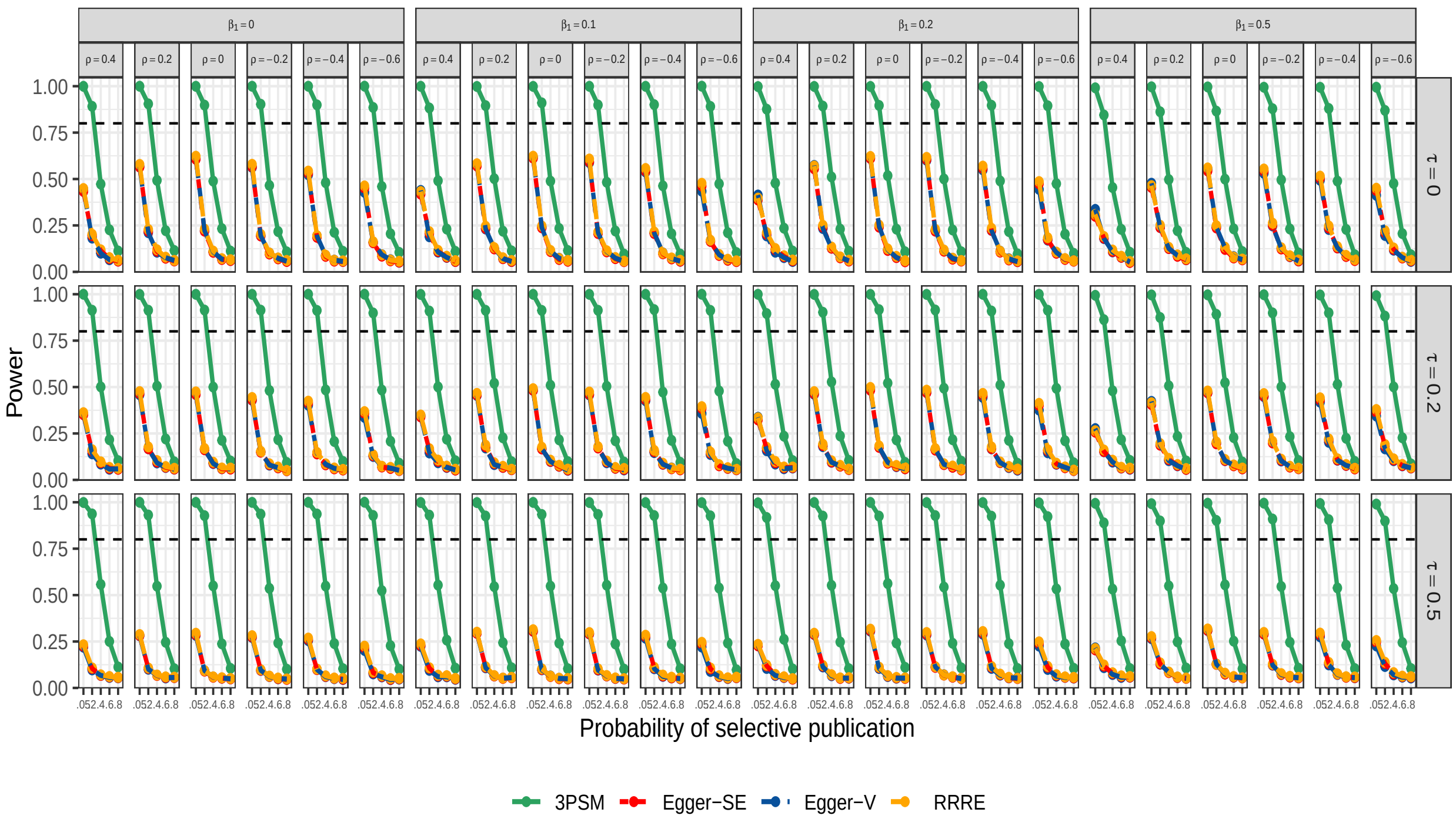

Figure S36: Power for meta-analyses with $k = 50$ studies and $\beta_2 = 0.5$.

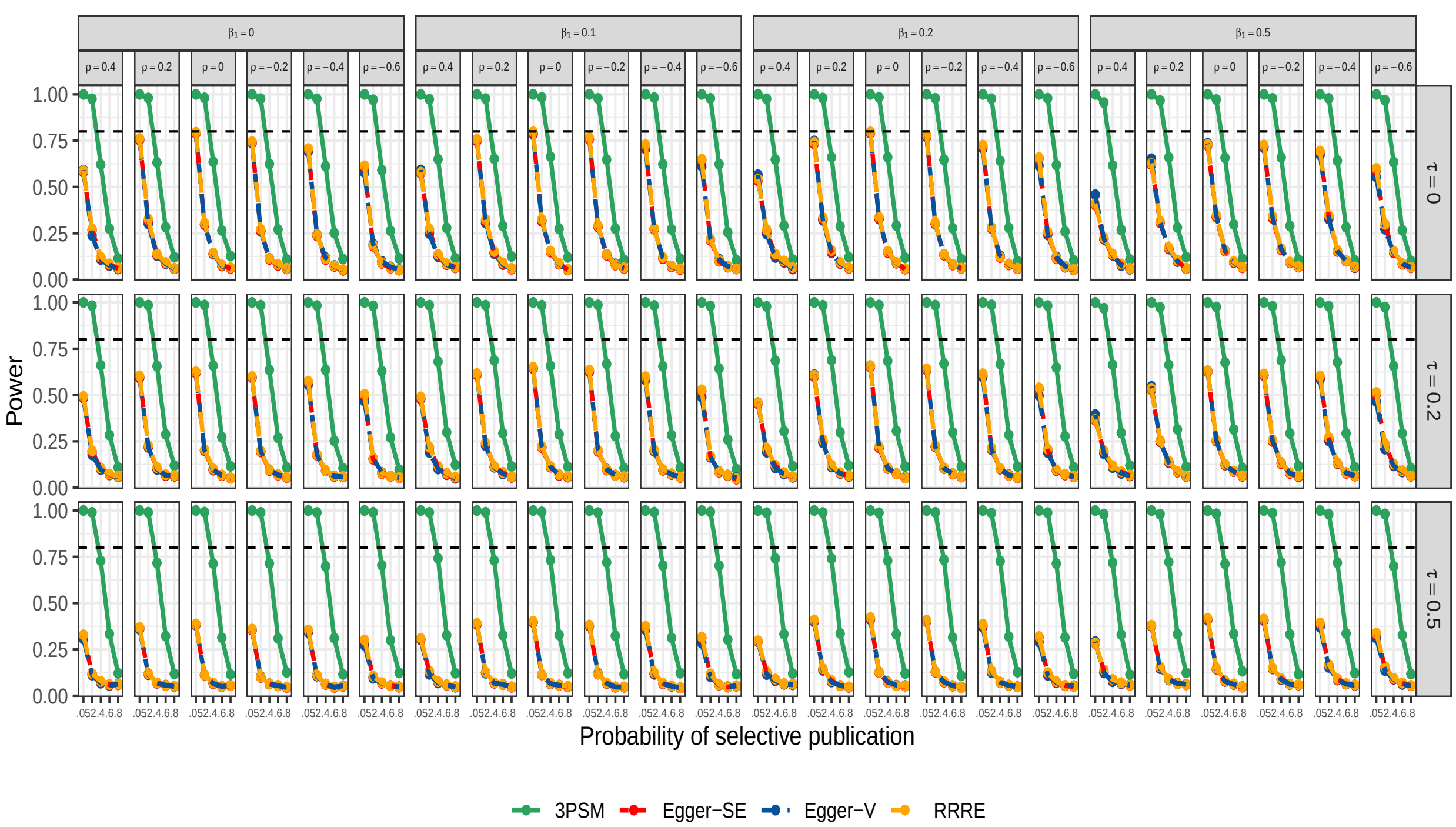

Figure S37: Power for meta-analyses with $k = 80$ studies and $\beta_2 = 0.5$.

S4 Model misspecification

This section presents the Type I error rates and power of 3PSM and Egger-SE when one or both covariates were unrelated to the true effect sizes. When neither covariate was related to the true effects ($\beta_1 = \beta_2 = 0$), Egger-SE had well-calibrated Type I error rates near the nominal level across conditions, while 3PSM likelihood ratio test showed slight inflated Type I error, especially under small residual heterogeneity (e.g., $\tau = 0$). Including irrelevant covariates tended to slightly inflate the Type I error rates of the 3PSM likelihood ratio test when the residual heterogeneity and number of studies were small because of the increased model complexity and estimation uncertainty. However, when the continuous covariate was related to the true effect ($\beta_1 = 0$, $\beta_2 \neq 0$) but unmodeled, Egger-SE had increasingly inflated rejection rates as confounding became more severe, whereas 3PSM tended to have stable, and sometimes slightly inflated, rejection rates. The 3PSM likelihood ratio test consistently outperformed Egger-SE in power across conditions. Including irrelevant covariates slightly reduced the power of Egger-SE but had little effect on the power of 3PSM.

S4.1 Type I error rates when $\beta_1 = \beta_2 = 0$

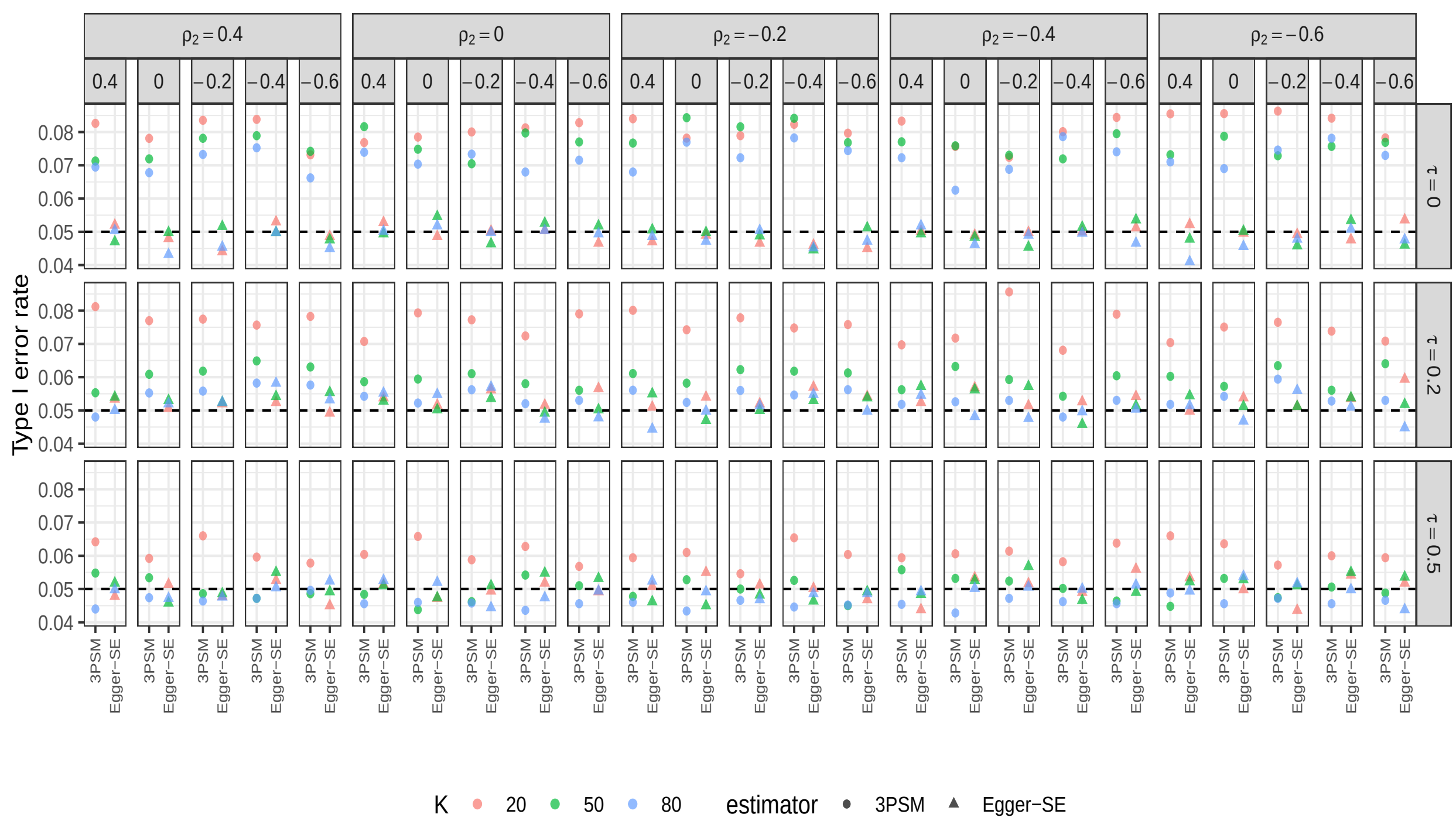

Figure S38: Type I error rates ignoring covariates when $\beta_1 = \beta_2 = 0$.

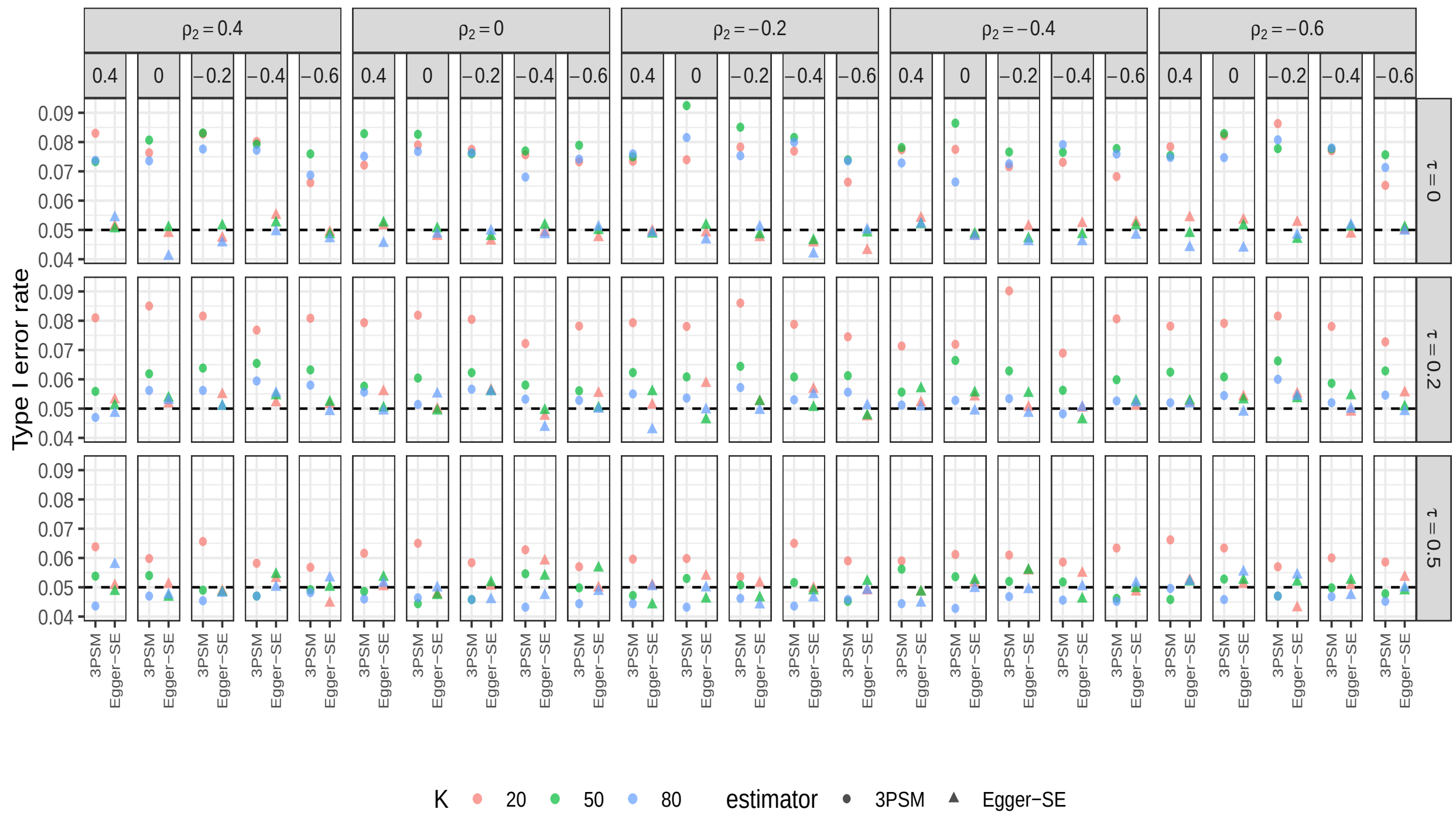

Figure S39: Type I error rates modeling binary covariate when $\beta_1 = \beta_2 = 0$.

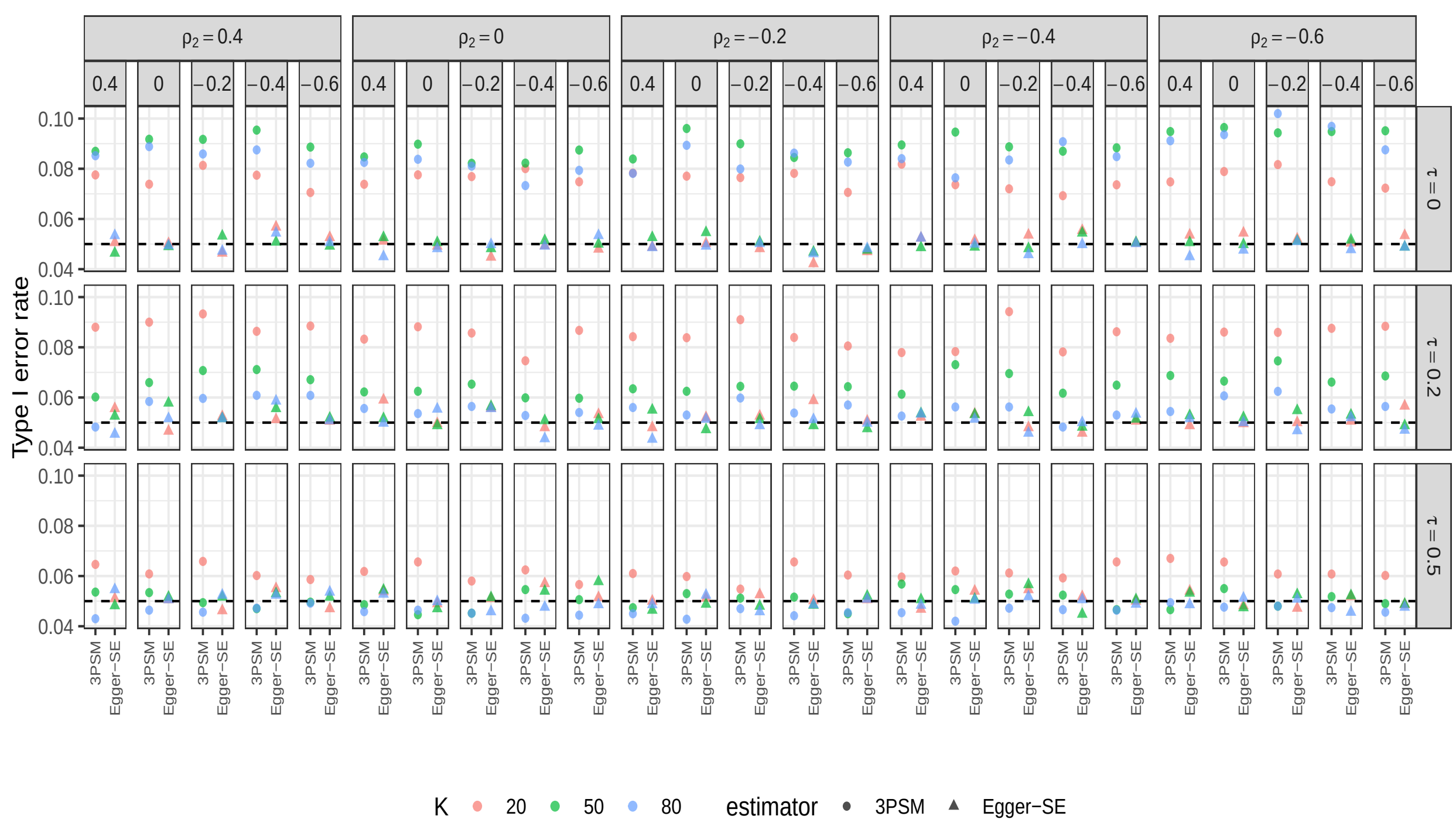

Figure S40: Type I error rates modeling both covariates when $\beta_1 = \beta_2 = 0$.

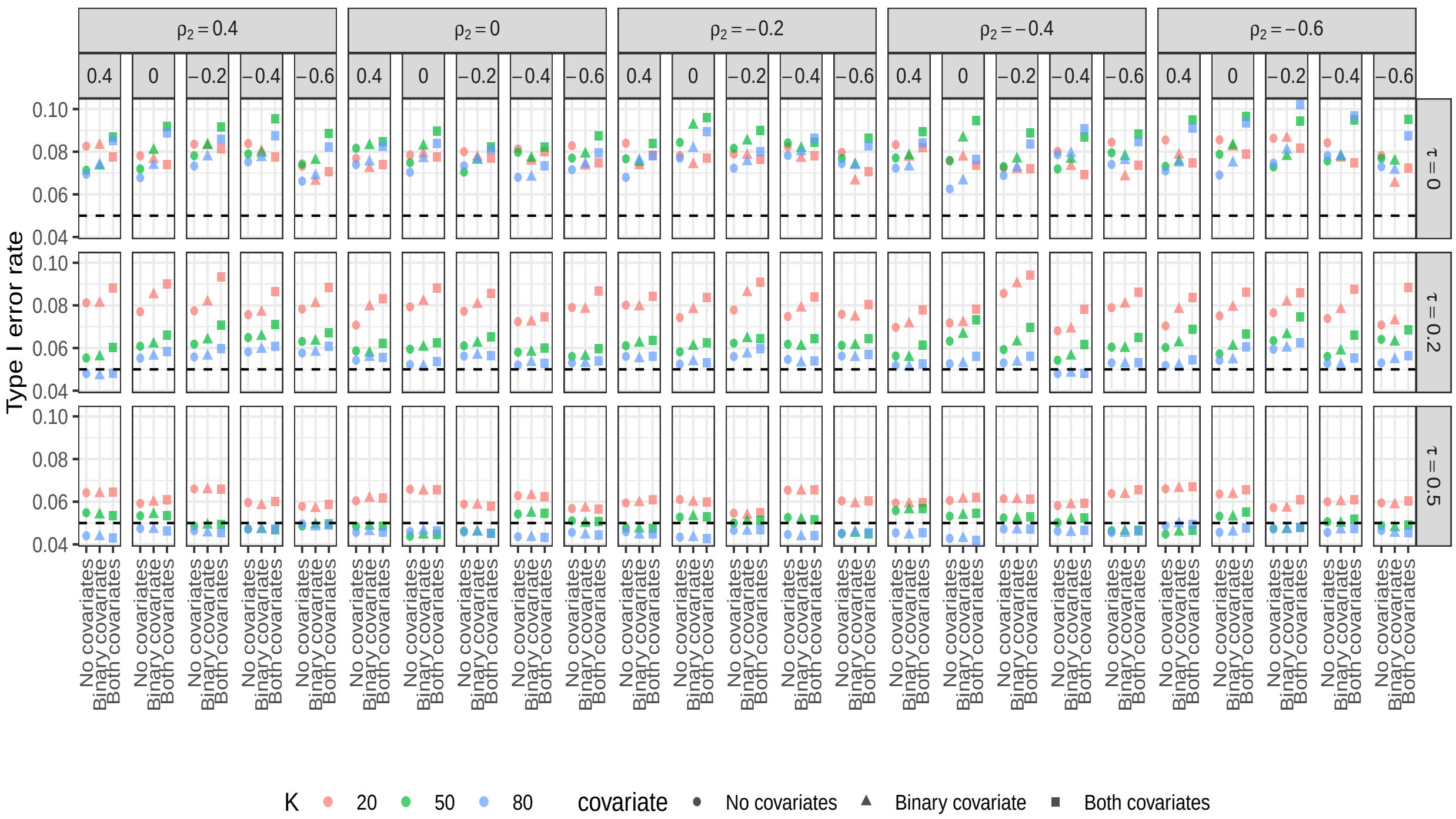

Figure S41: Type I error rates of 3PSM when $\beta_1 = \beta_2 = 0$.

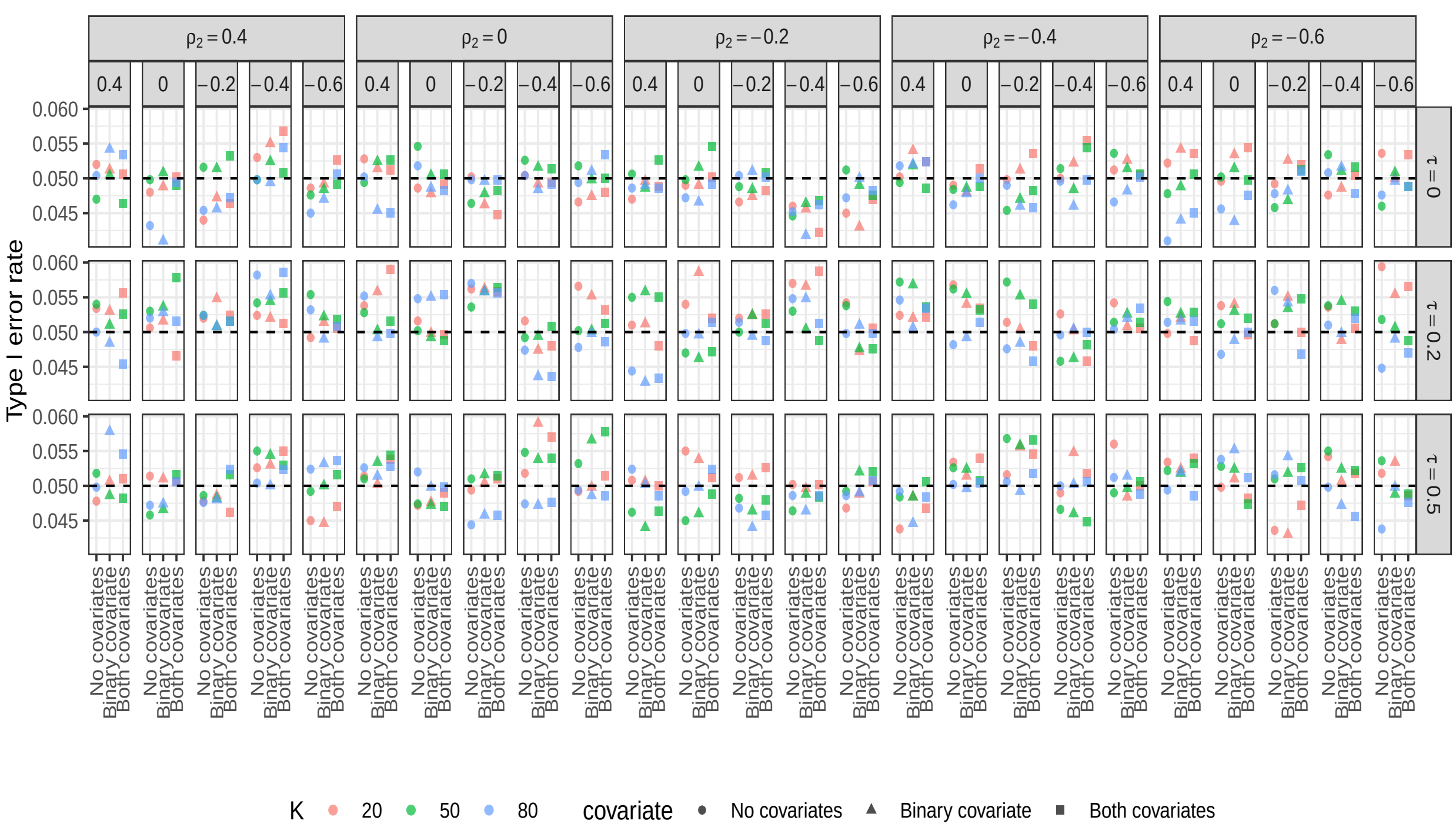

Figure S42: Type I error rates of Egger-SE when $\beta_1 = \beta_2 = 0$.

S4.2 Power when $\beta_1 = \beta_2 = 0$

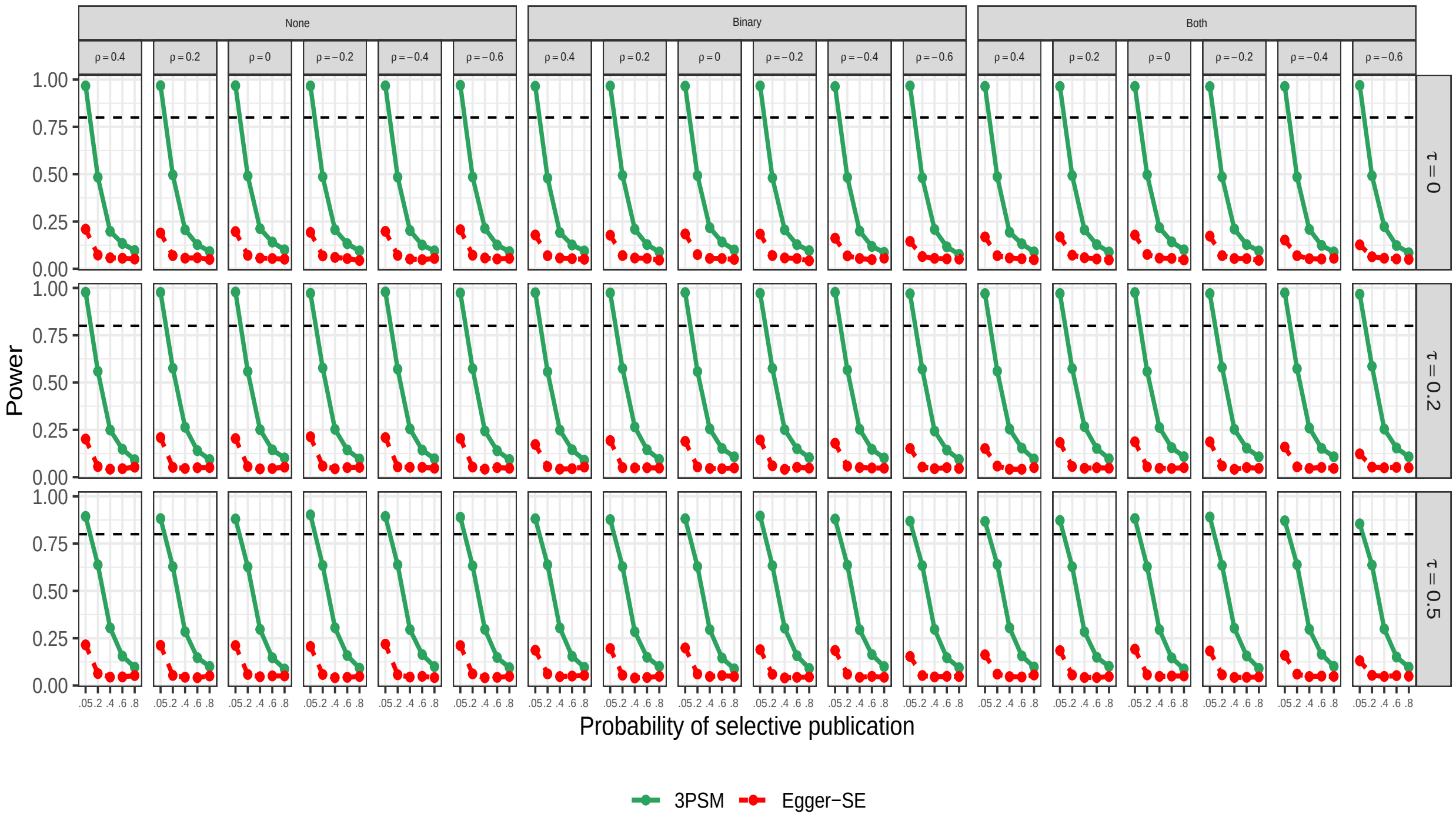

Figure S43: Power when $\beta_1 = \beta_2 = 0$ and $k = 20$.

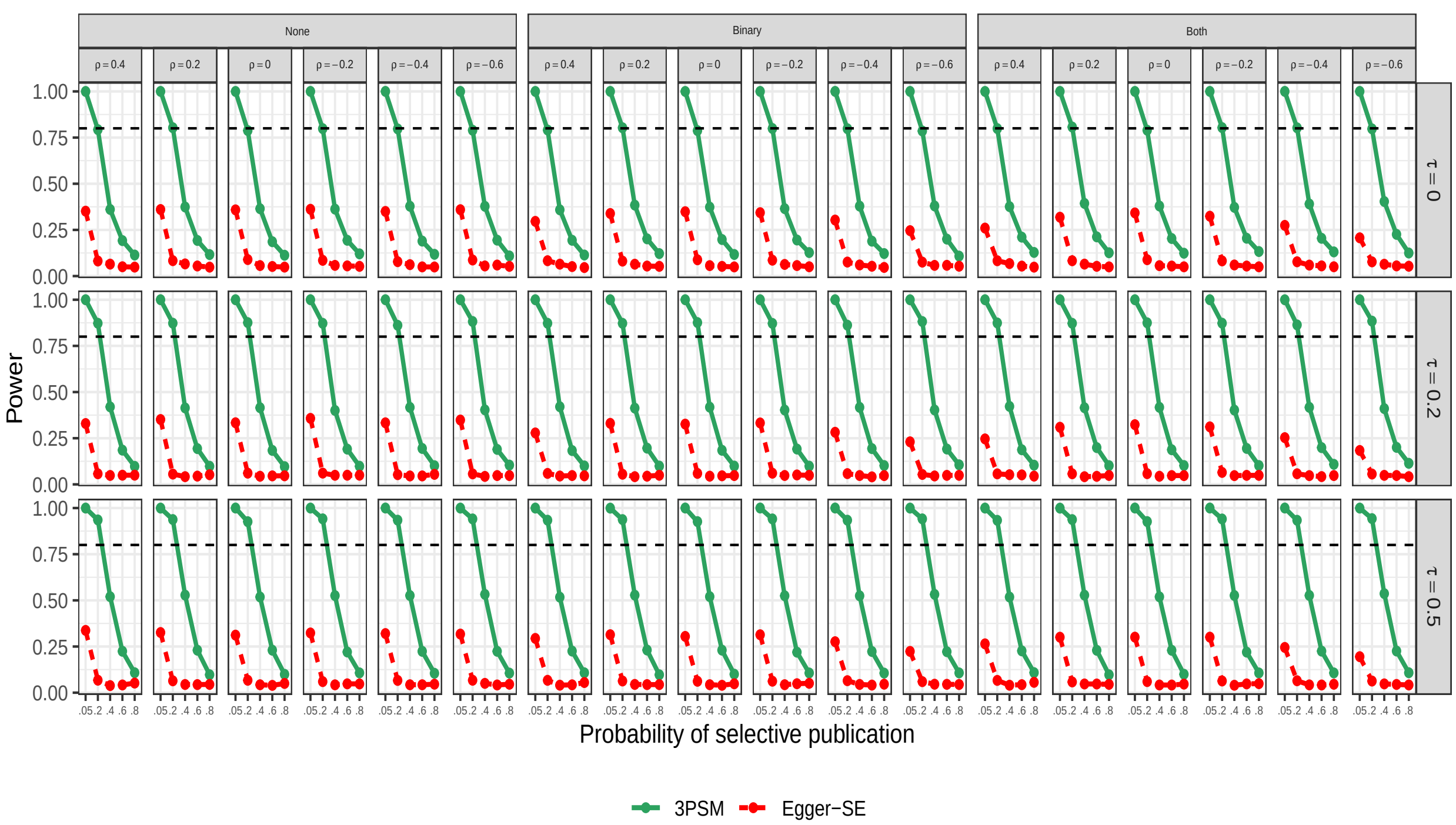

Figure S44: Power when $\beta_1 = \beta_2 = 0$ and $k = 50$.

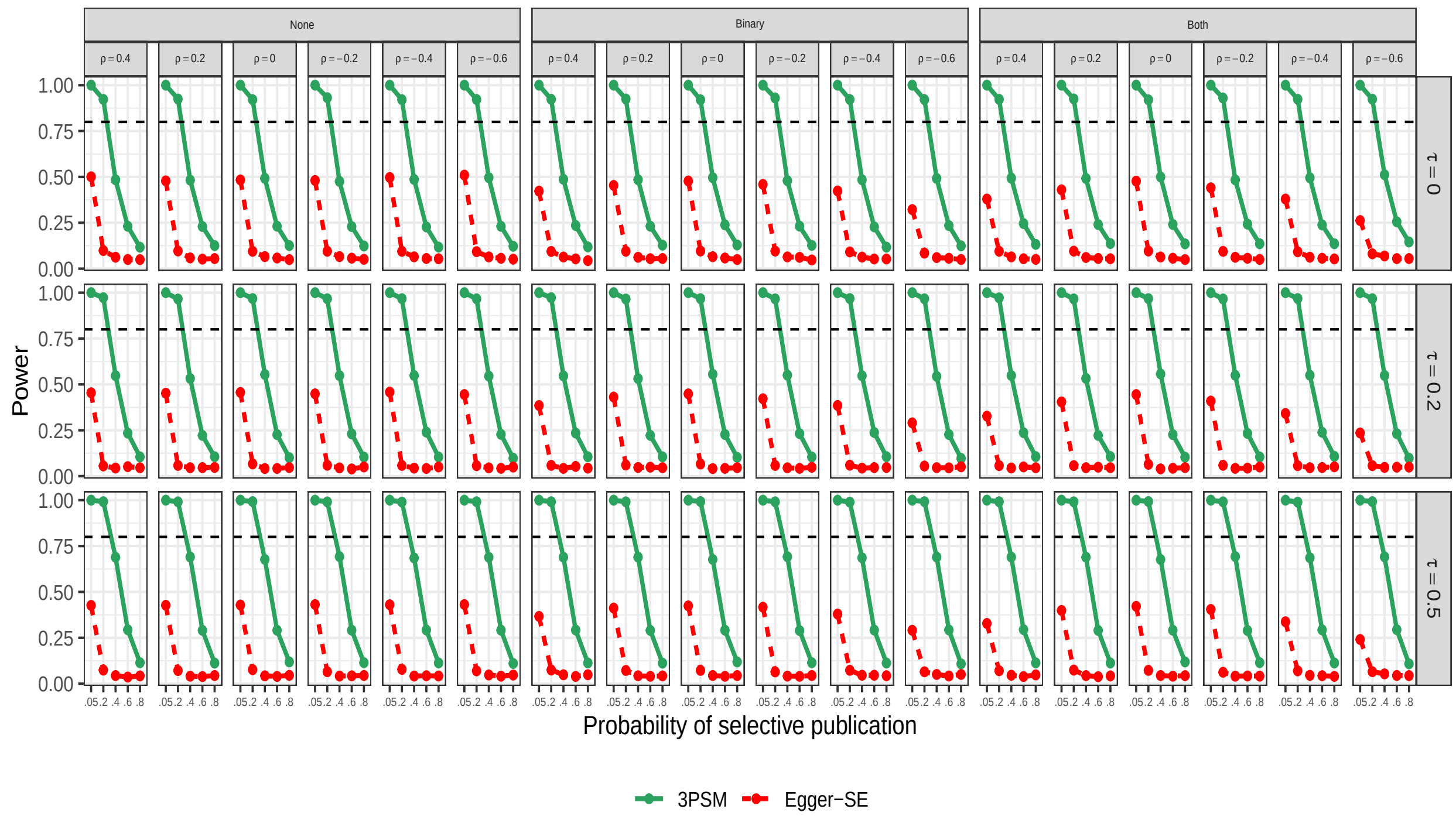

Figure S45: Power when $\beta_1 = \beta_2 = 0$ and $k = 80$.

S4.2.1 Power of Egger-SE when $\beta_1 = \beta_2 = 0$

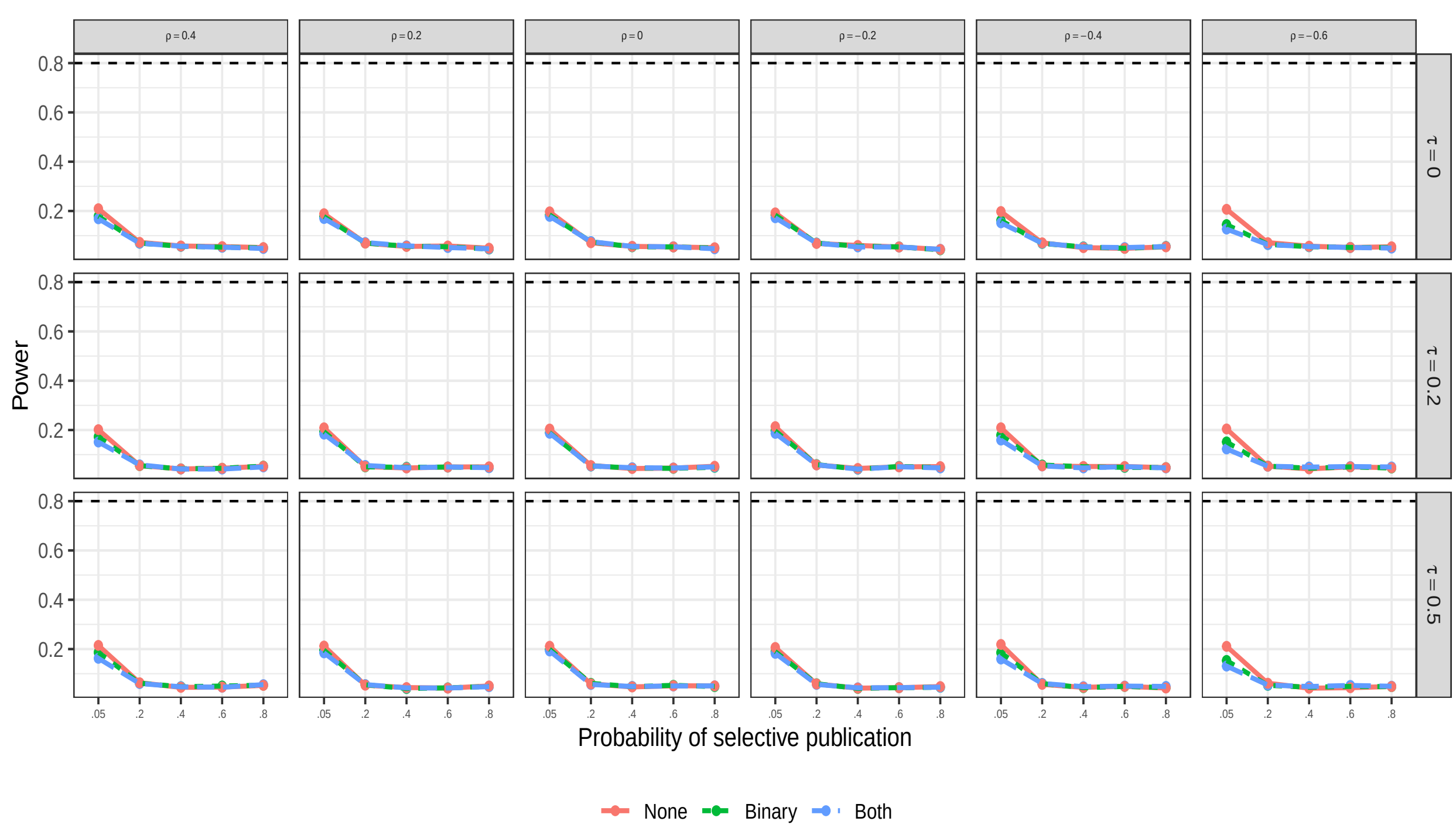

Figure S46: Power of Egger-SE when $\beta_1 = \beta_2 = 0$ and $k = 20$.

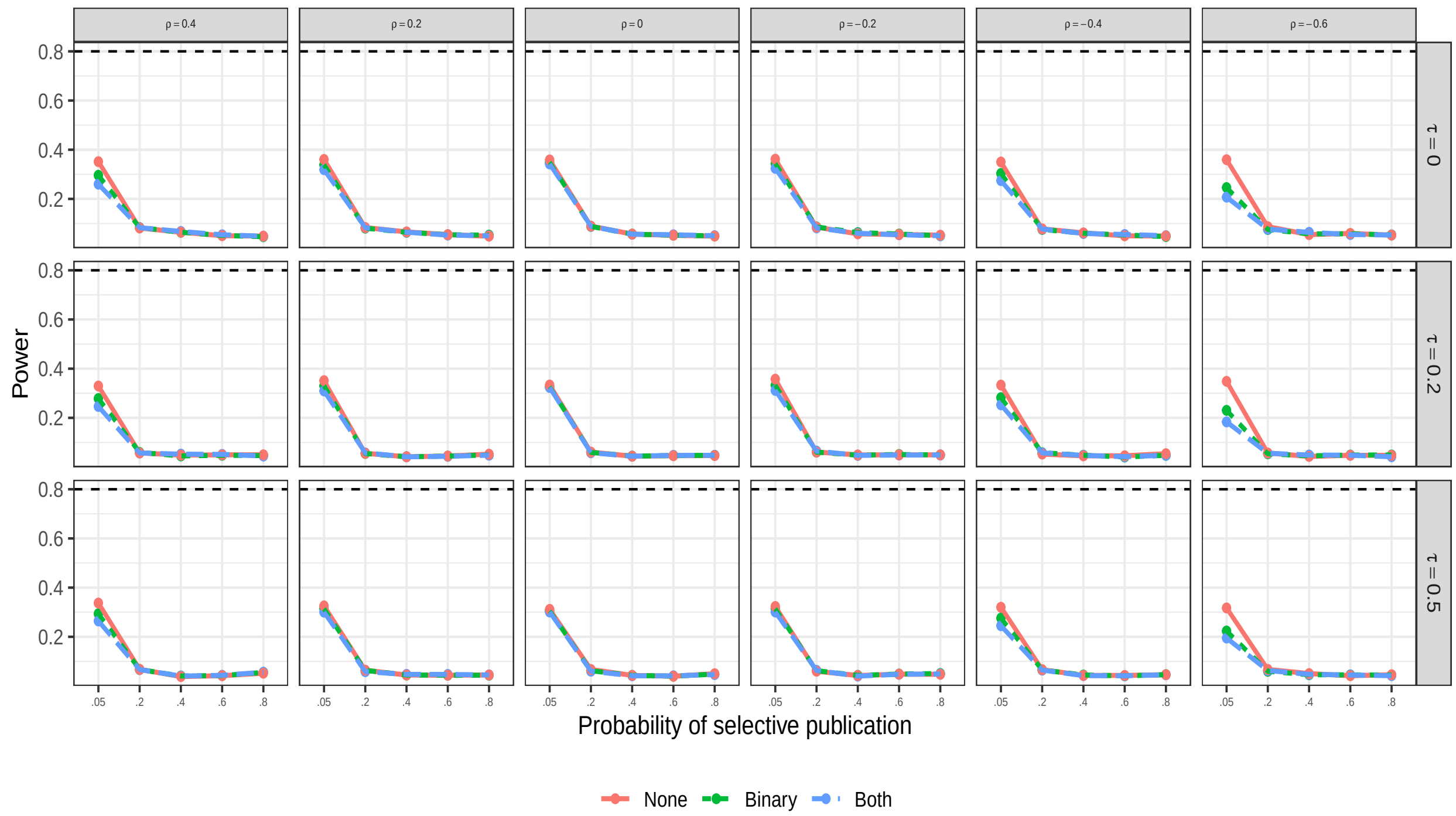

Figure S47: Power of Egger-SE when $\beta_1 = \beta_2 = 0$ and $k = 50$.

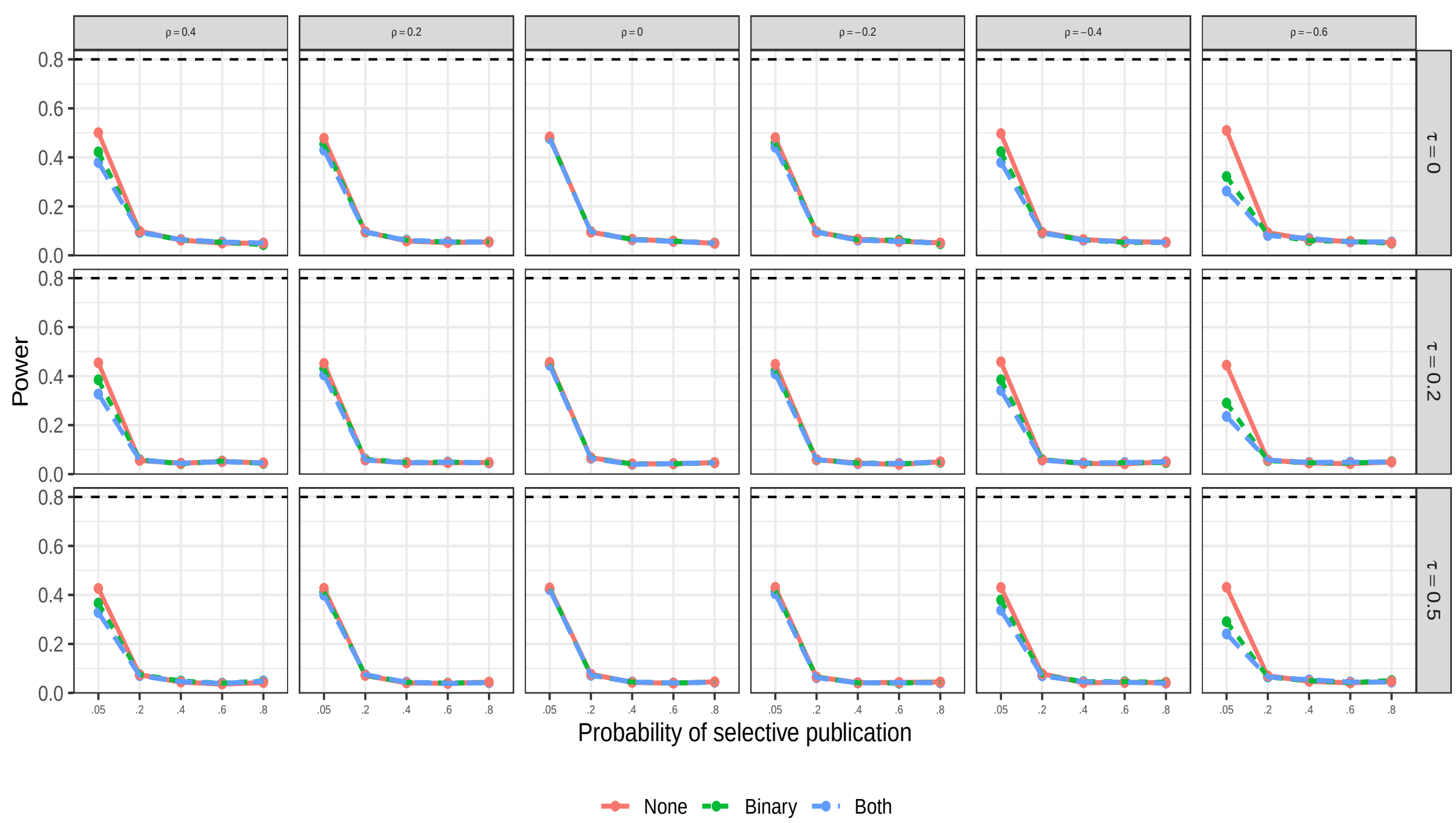

Figure S48: Power of Egger-SE when $\beta_1 = \beta_2 = 0$ and $k = 80$.

S4.2.2 Power of 3PSM when $\beta_1 = \beta_2 = 0$

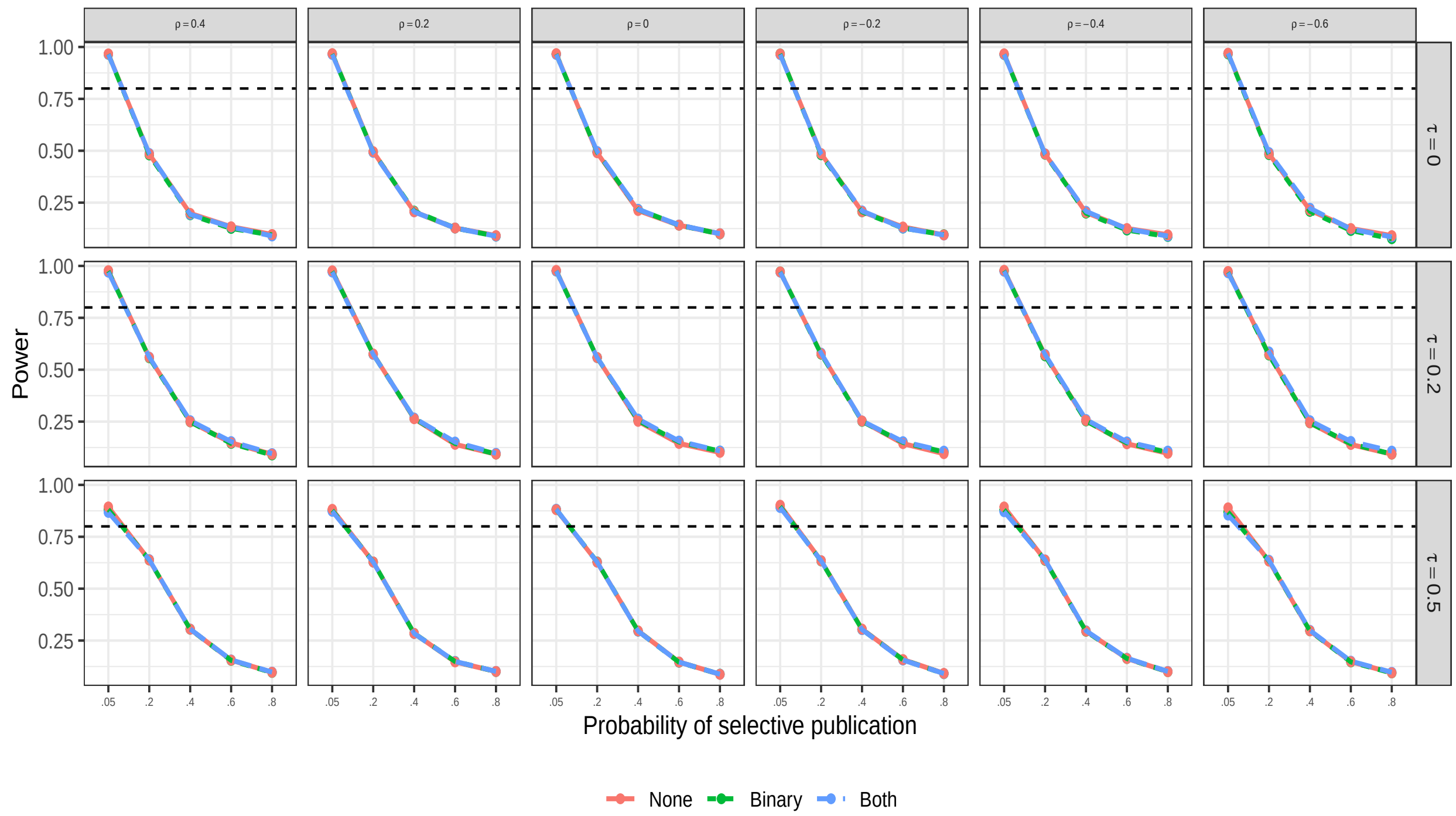

Figure S49: Power of 3PSM when $\beta_1 = \beta_2 = 0$ and $k = 20$.

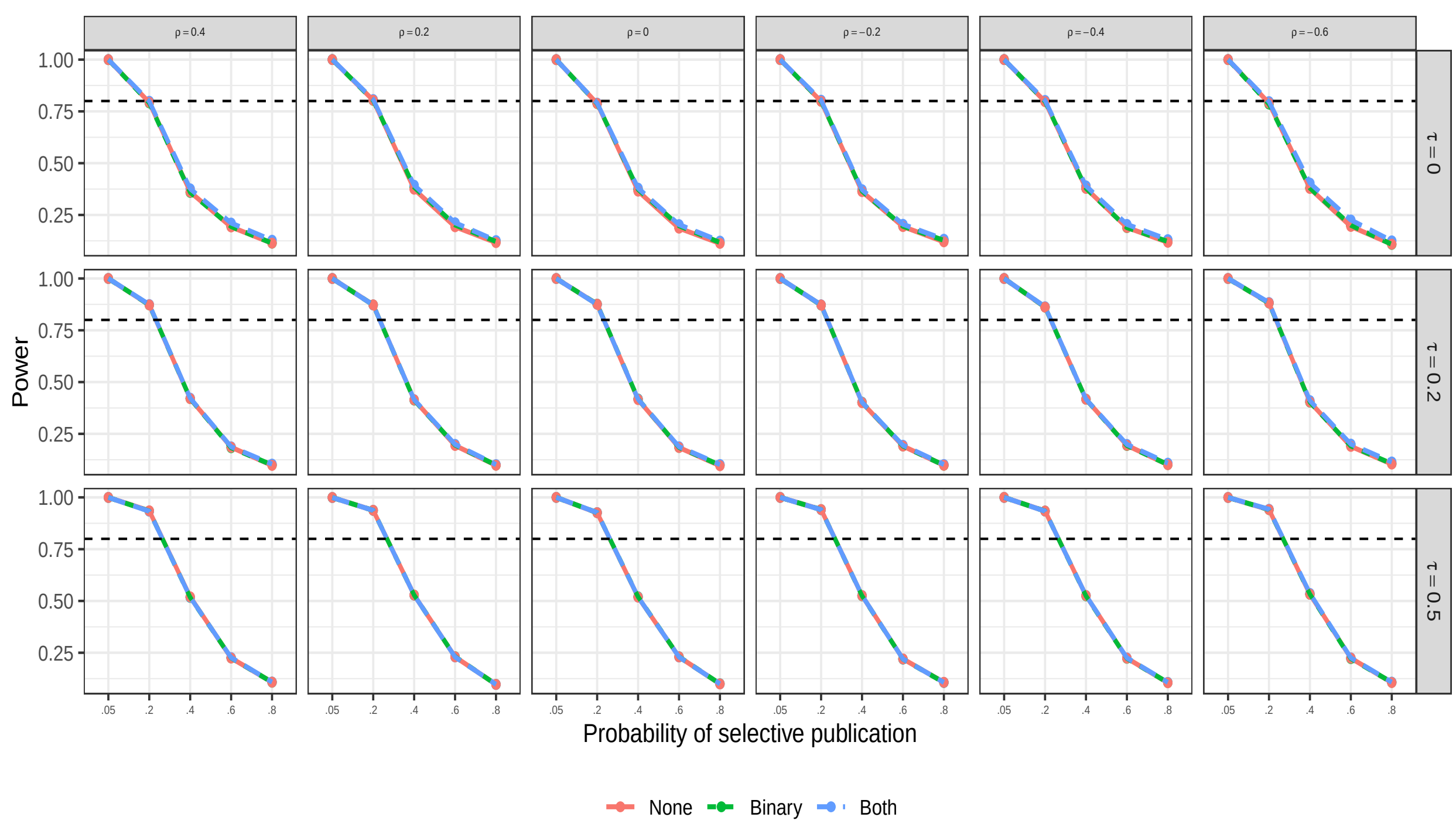

Figure S50: Power of 3PSM when $\beta_1 = \beta_2 = 0$ and $k = 50$.

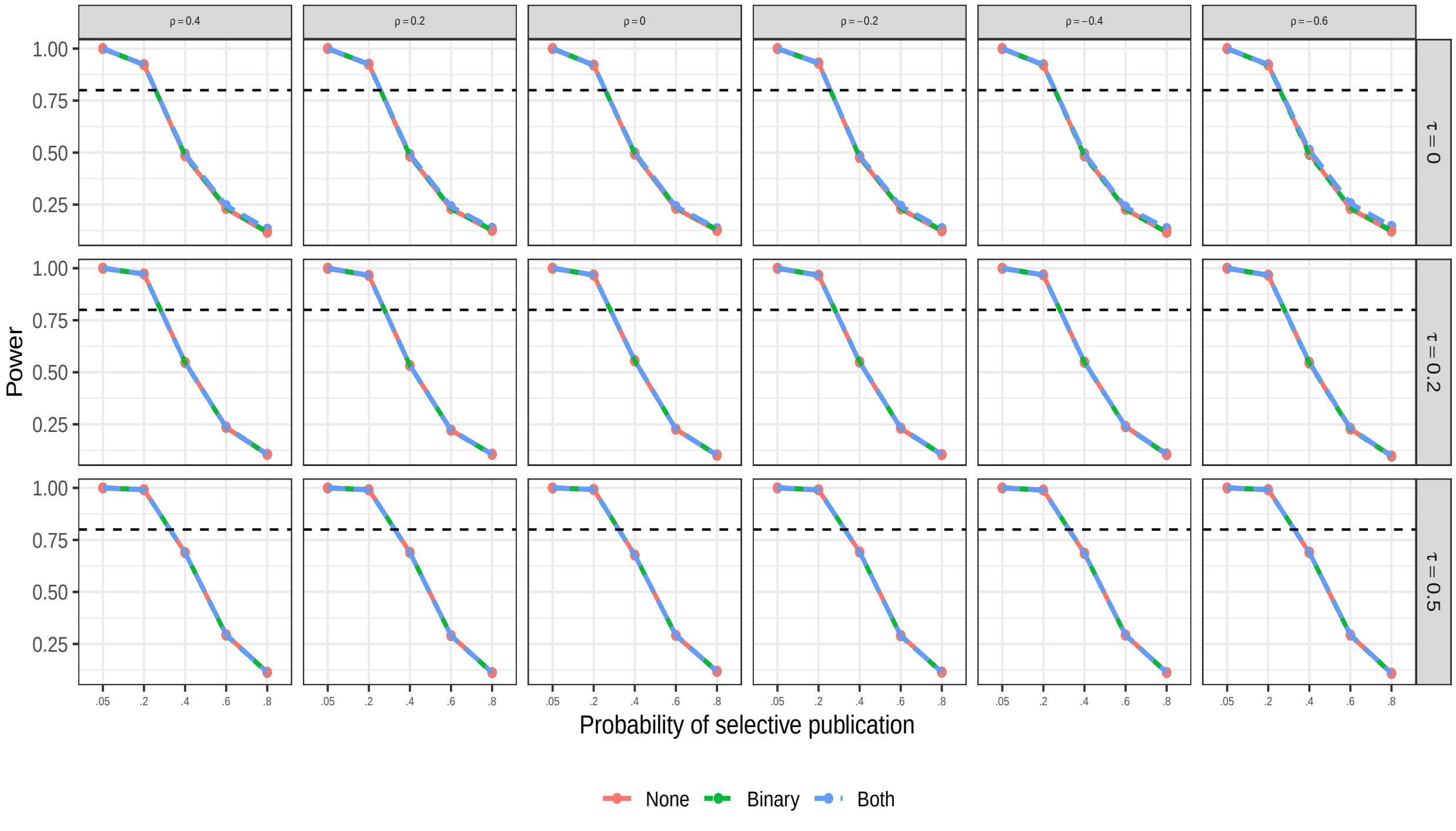

Figure S51: Power of 3PSM when $\beta_1 = \beta_2 = 0$ and $k = 80$.

S4.3 $\beta_1 = 0$, $\beta_2 \neq 0$

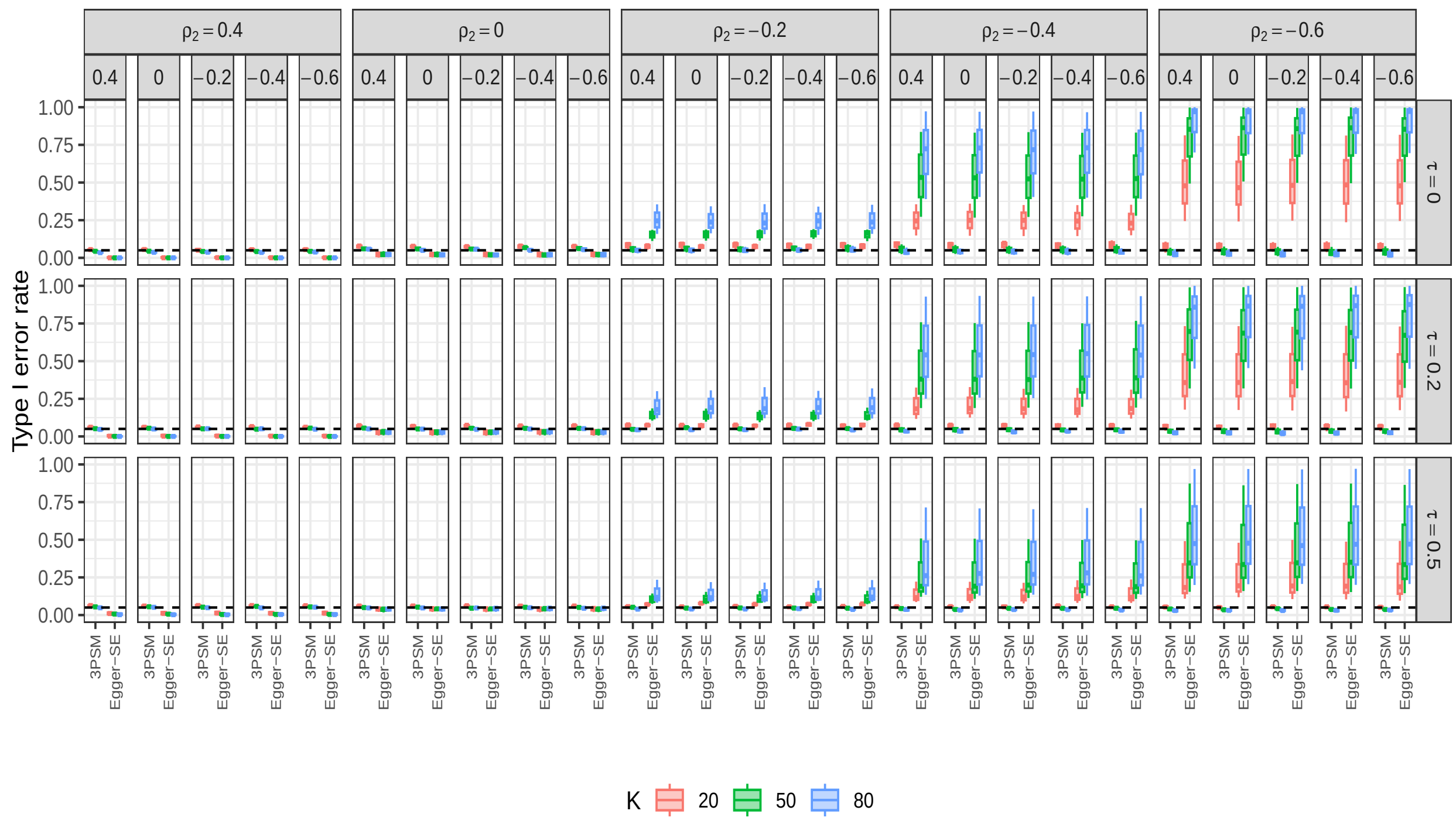

Figure S52: Type I error rates ignoring covariates when $\beta_1 = 0$ but $\beta_2 \neq 0$.

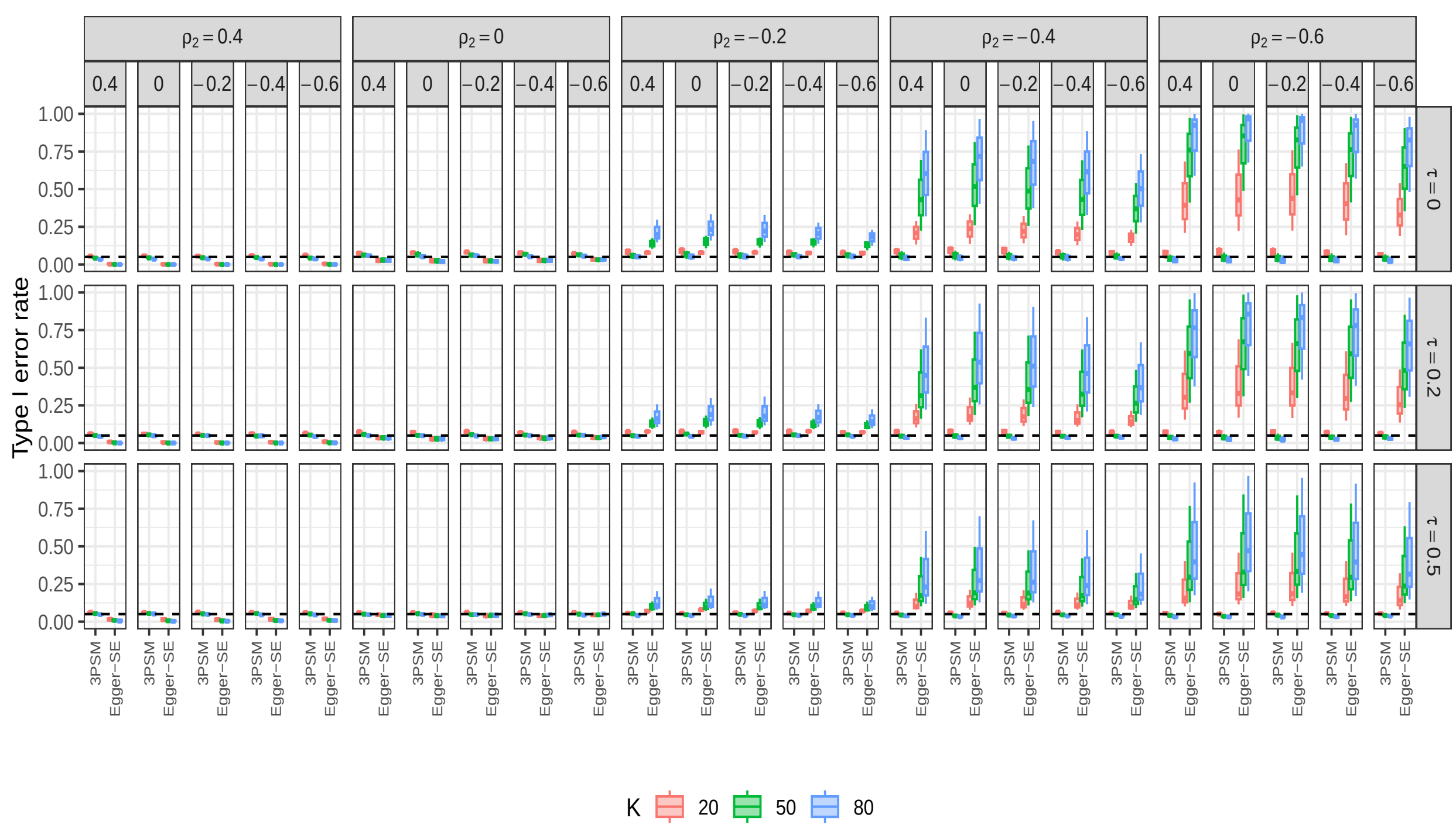

Figure S53: Type I error rates modeling binary covariate when $\beta_1 = 0$ but $\beta_2 \neq 0$.

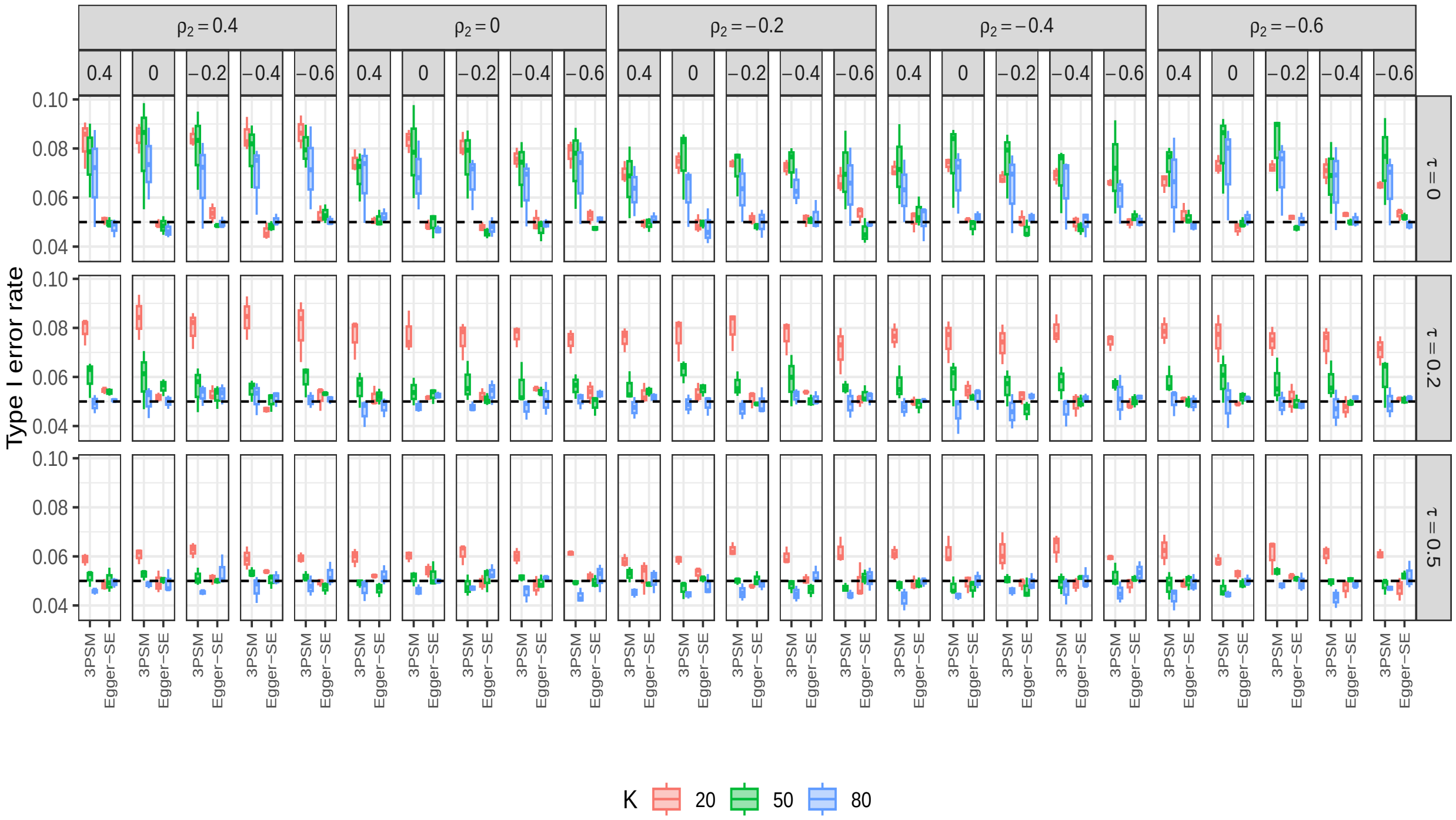

Figure S54: Type I error rates modeling both covariates when $\beta_1 = 0$ but $\beta_2 \neq 0$.

S4.3.1 $\beta_2 = 0.1$

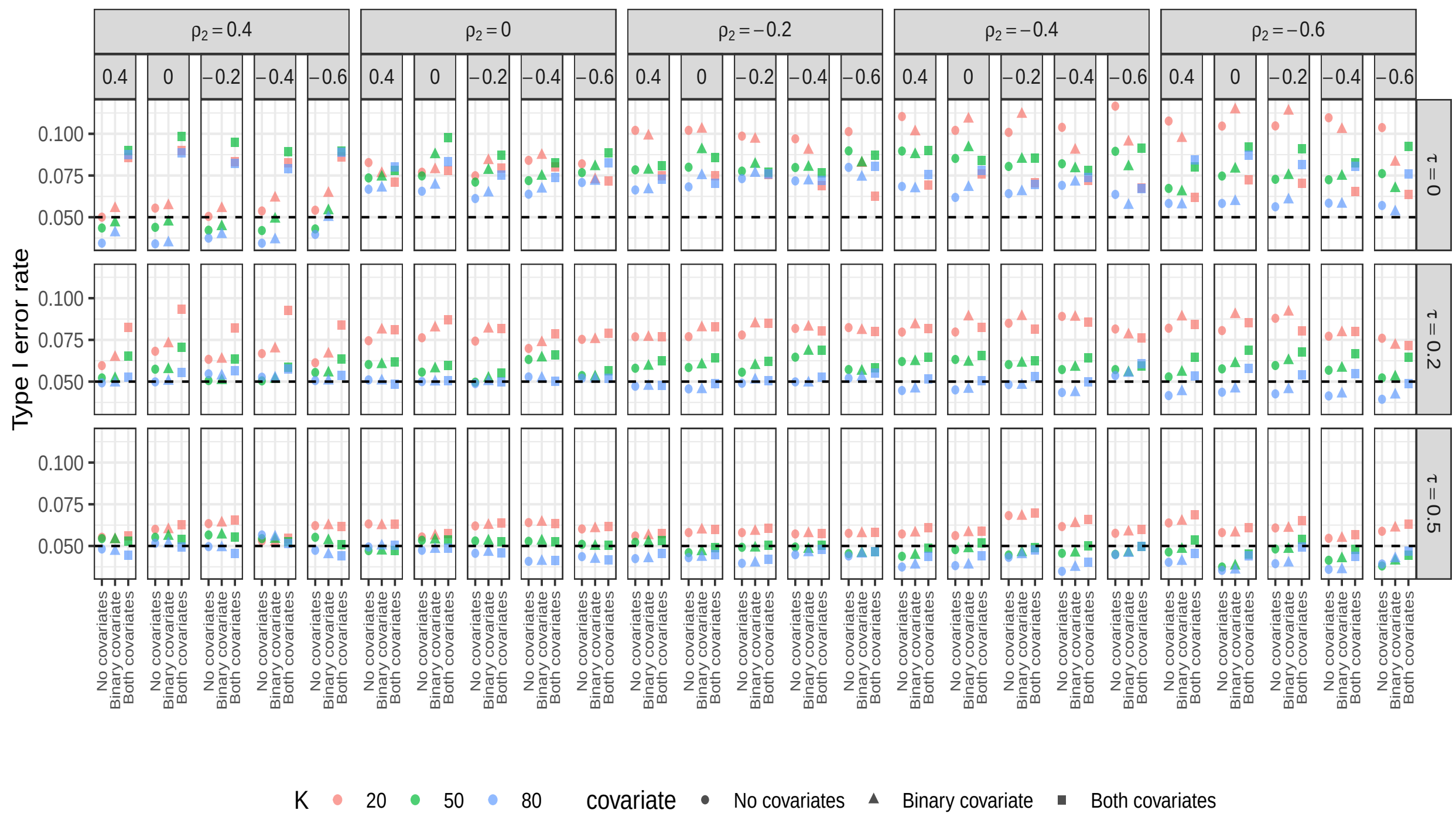

Figure S55: Type I error rates of 3PSM when $\beta_1 = 0$ but $\beta_2 = 0.1$.

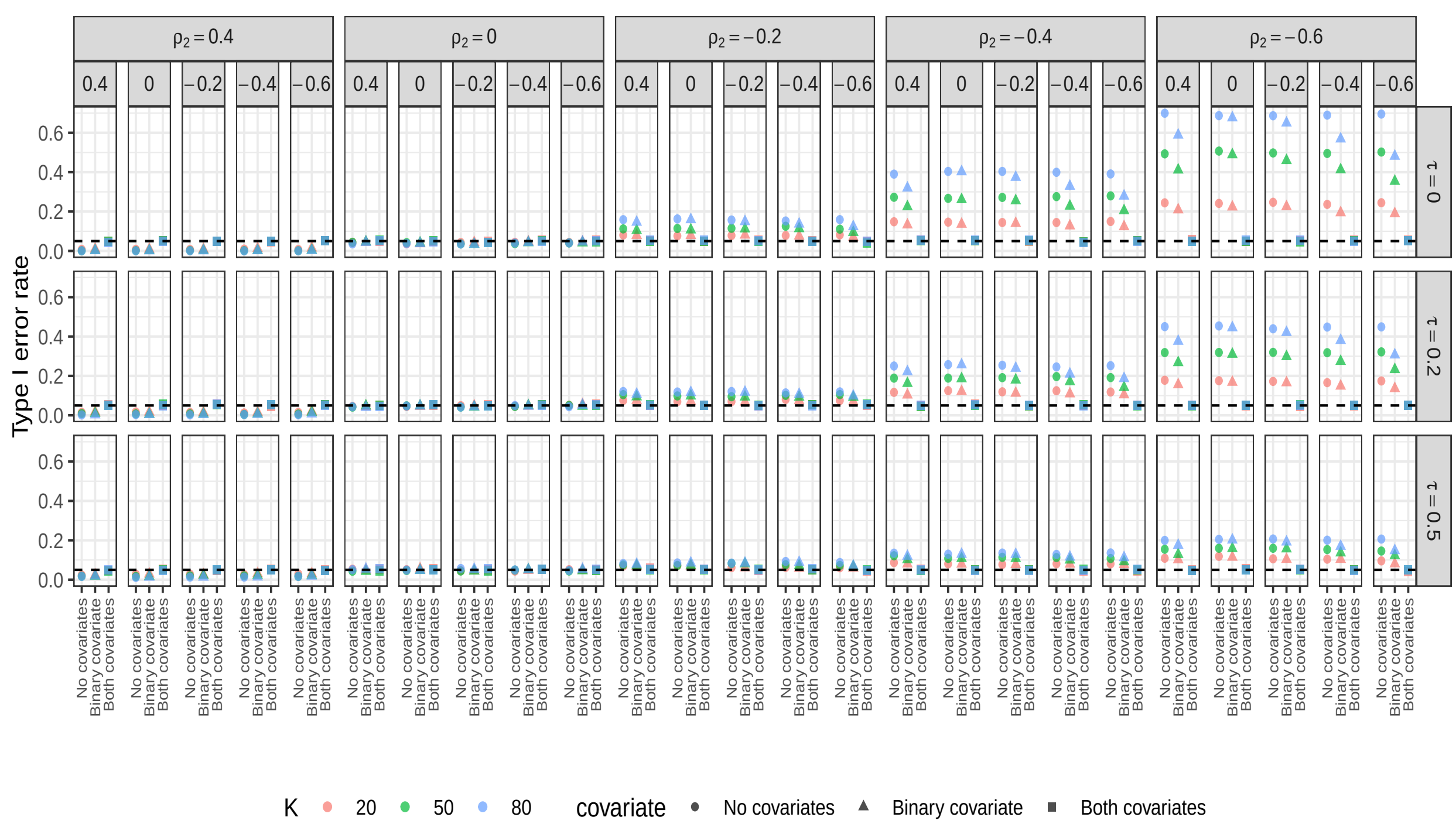

Figure S56: Type I error rates of Egger-SE when $\beta_1 = 0$ but $\beta_2 = 0.1$.

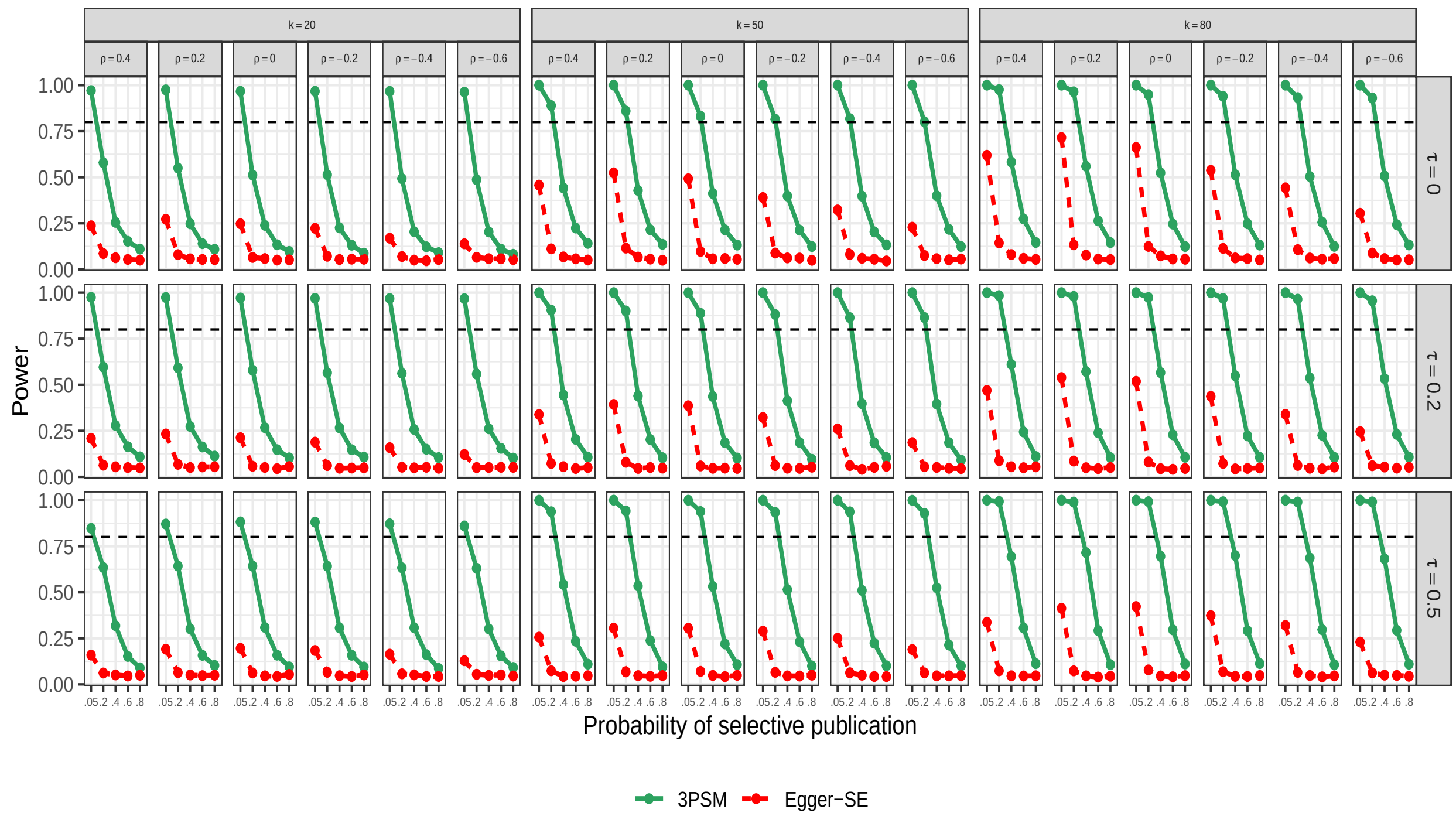

Figure S57: Power of Egger-SE and 3PSM modeling both covariates when $\beta_1 = 0$ and $\beta_2 = 0.1$.

S4.3.2 $\beta_2 = 0.2$

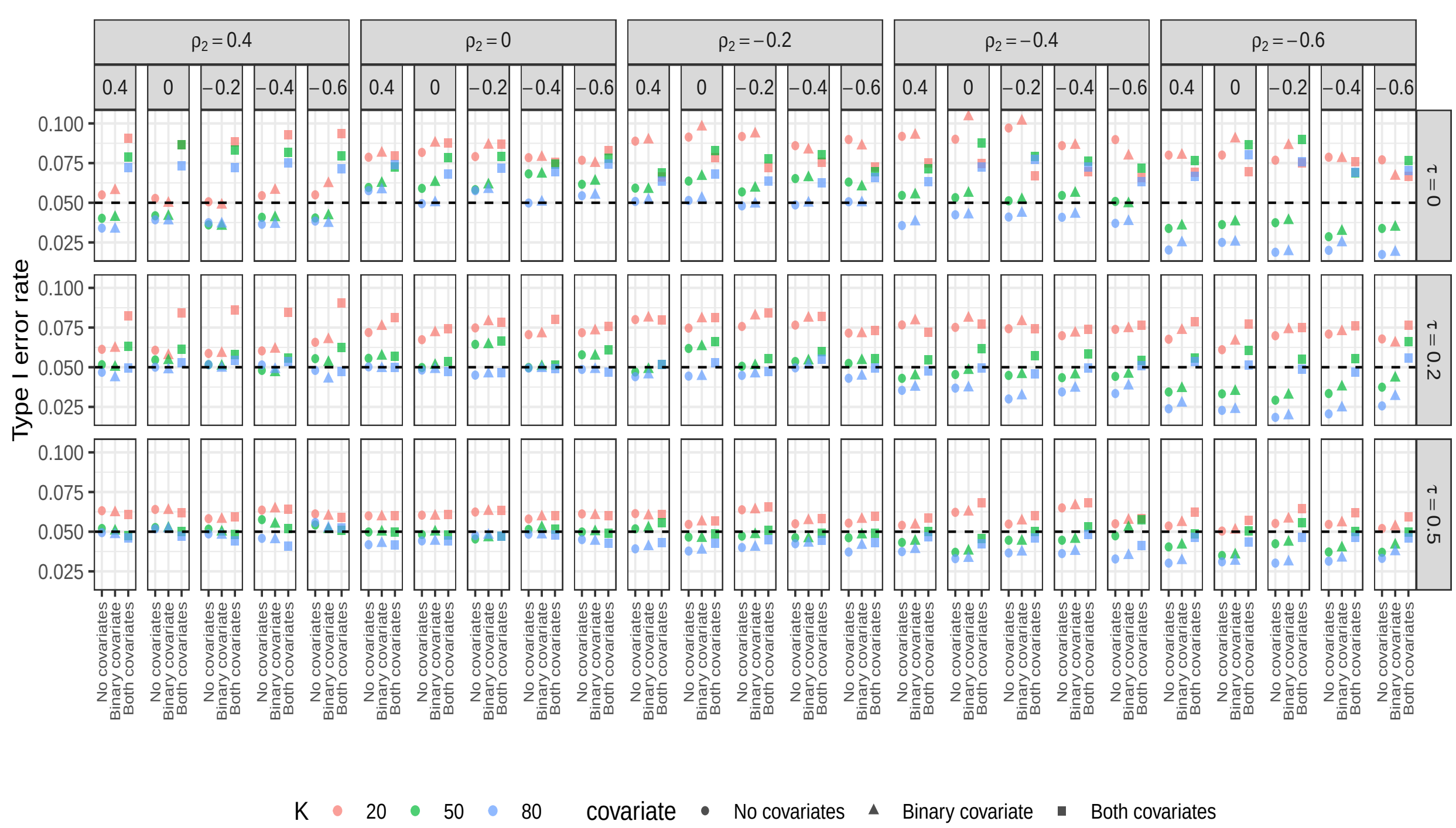

Figure S58: Type I error rates of 3PSM when $\beta_1 = 0$ but $\beta_2 = 0.2$.

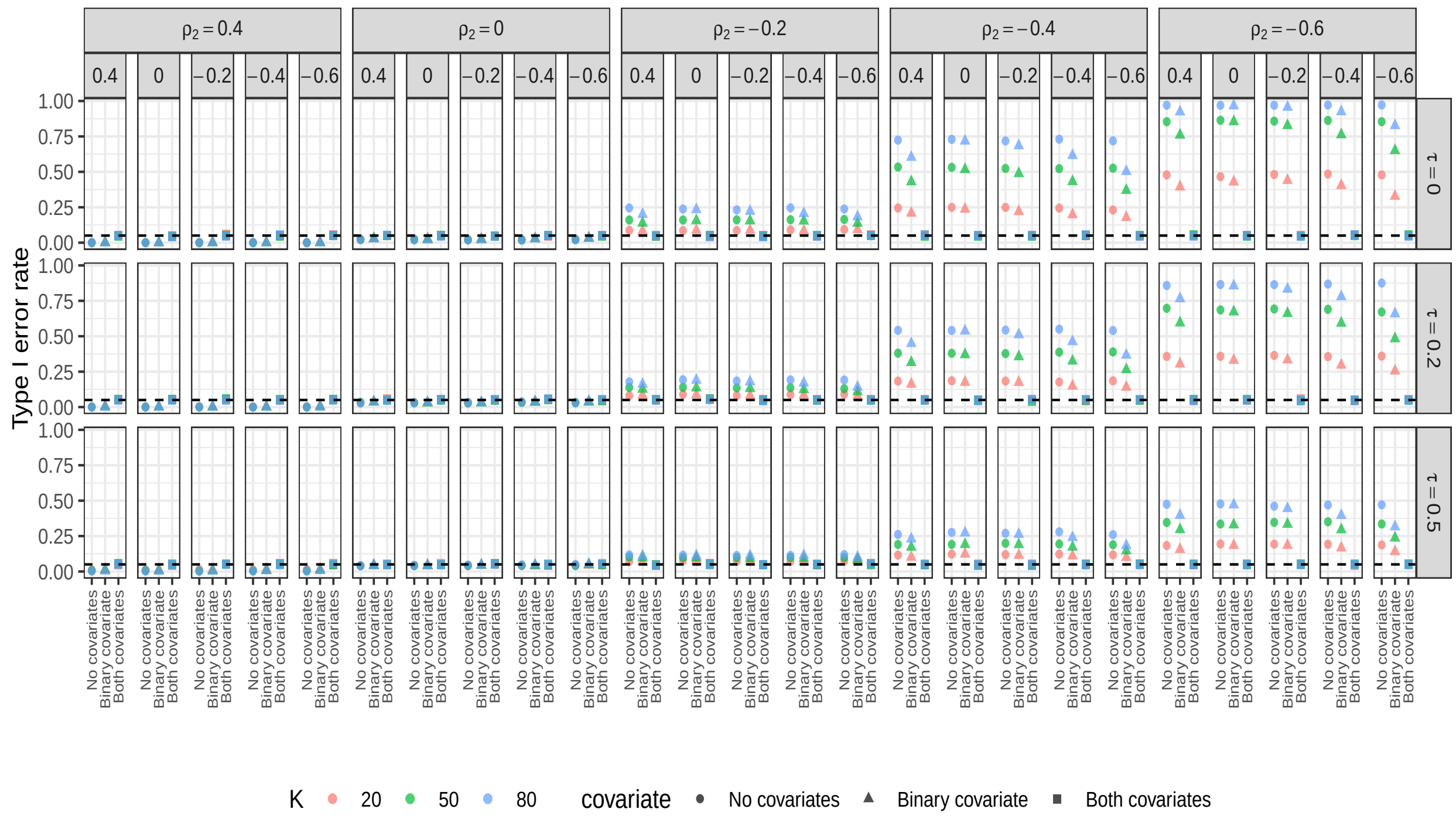

Figure S59: Type I error rates of Egger-SE when $\beta_1 = 0$ but $\beta_2 = 0.2$.

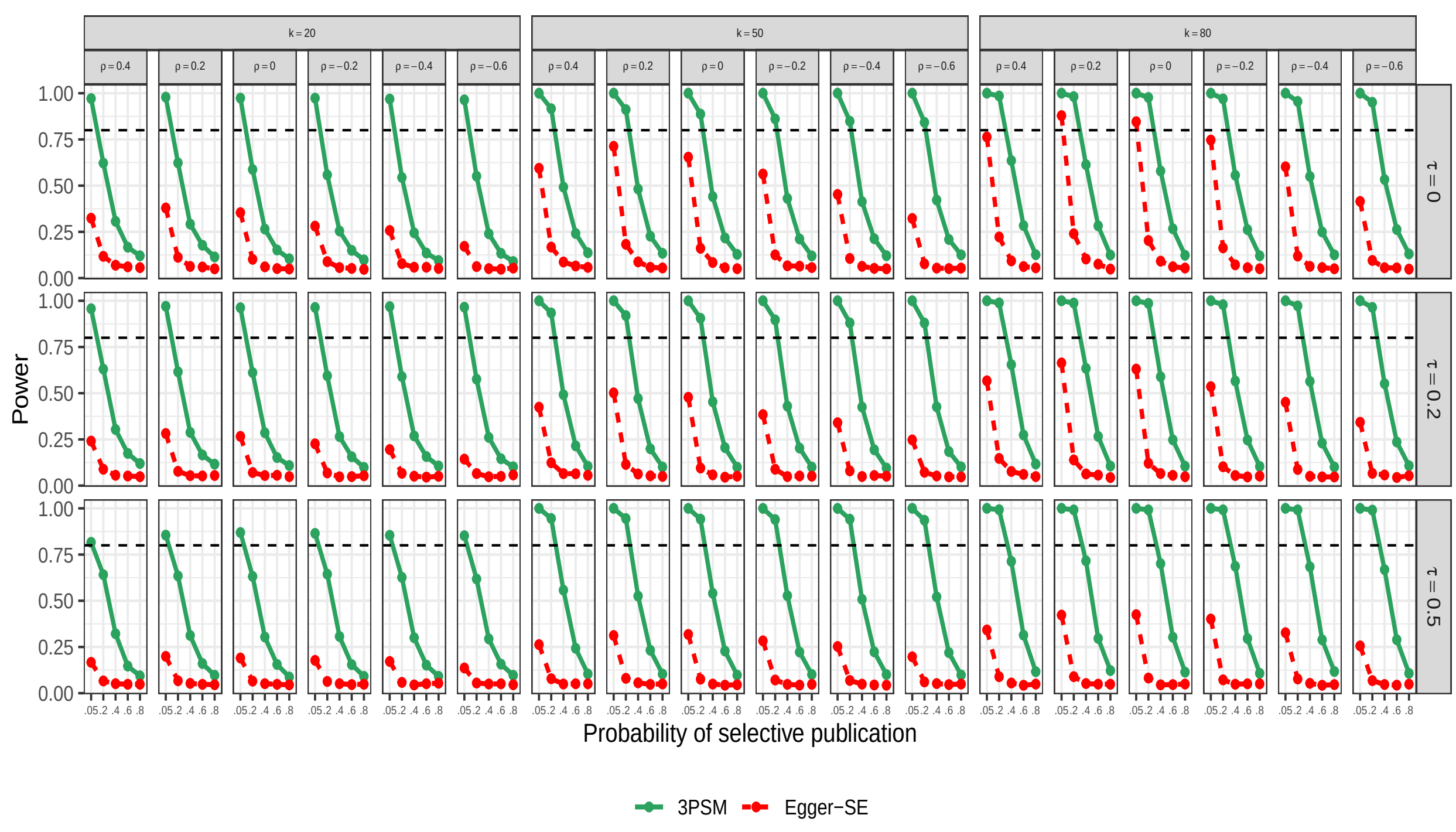

Figure S60: Power of Egger-SE and 3PSM modeling both covariates when $\beta_1 = 0$ and $\beta_2 = 0.2$.

S4.3.3 $\beta_2 = 0.5$

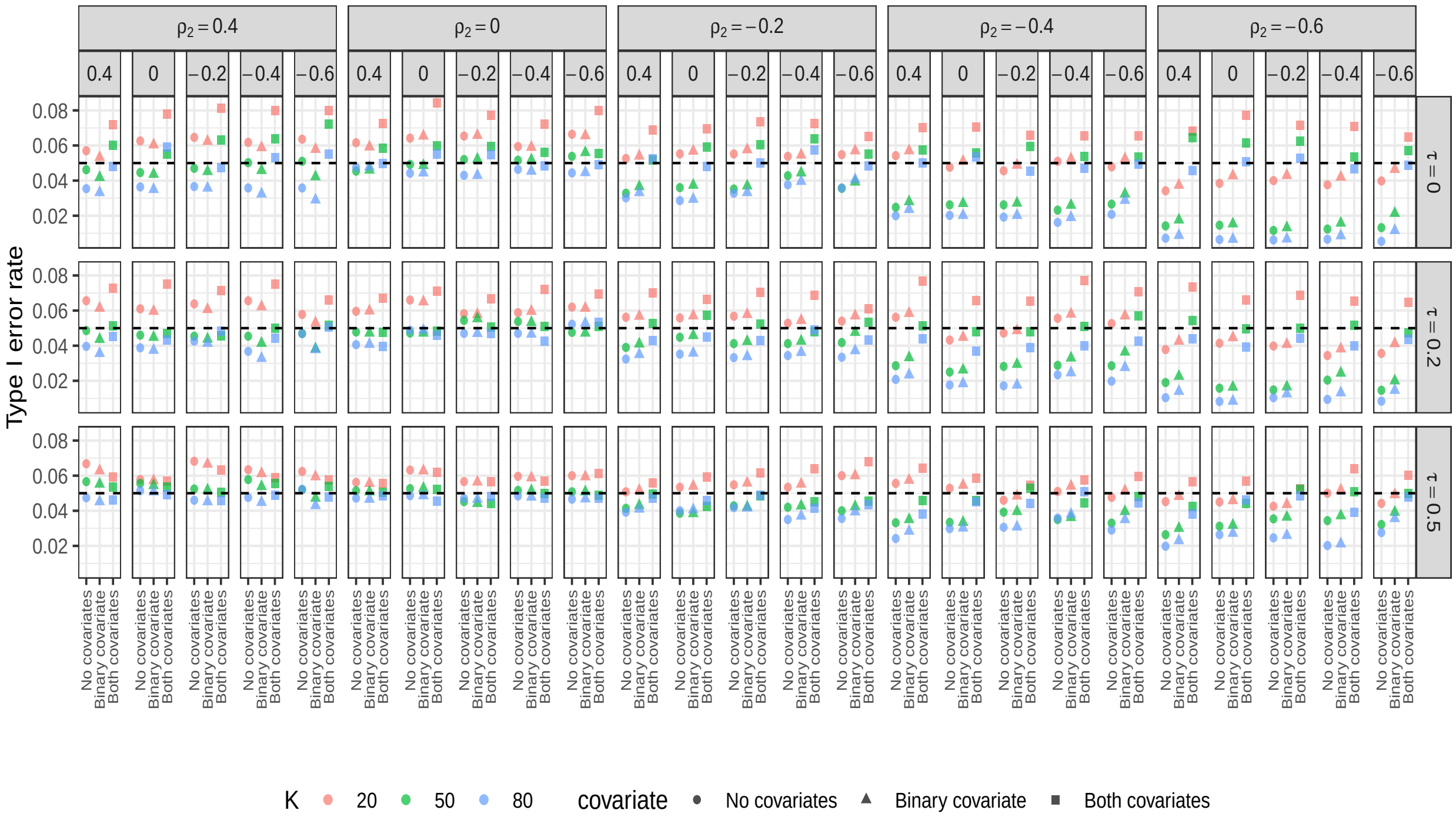

Figure S61: Type I error rates of 3PSM when $\beta_1 = 0$ but $\beta_2 = 0.5$.

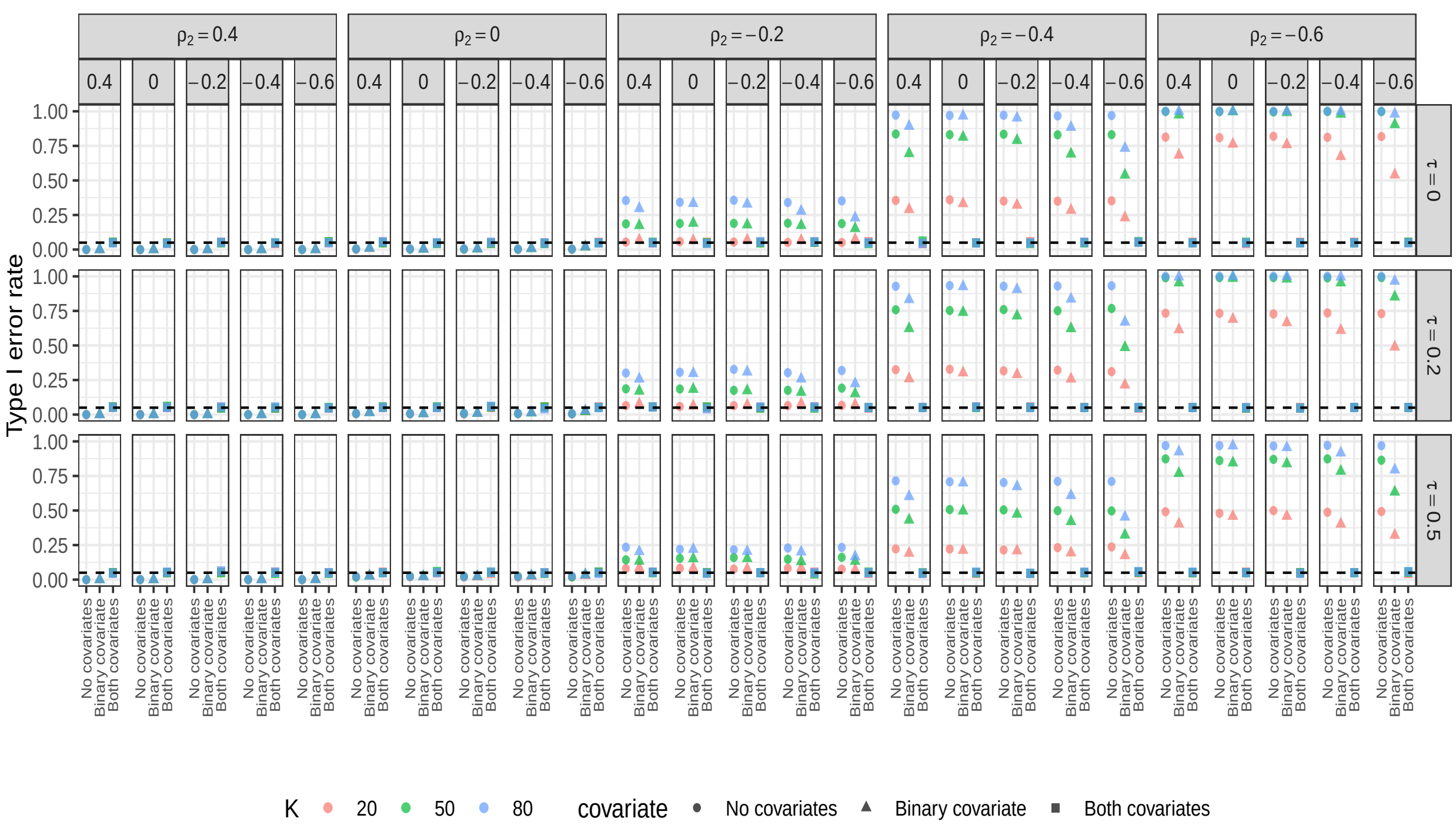

Figure S62: Type I error rates of Egger-SE when $\beta_1 = 0$ but $\beta_2 = 0.5$.

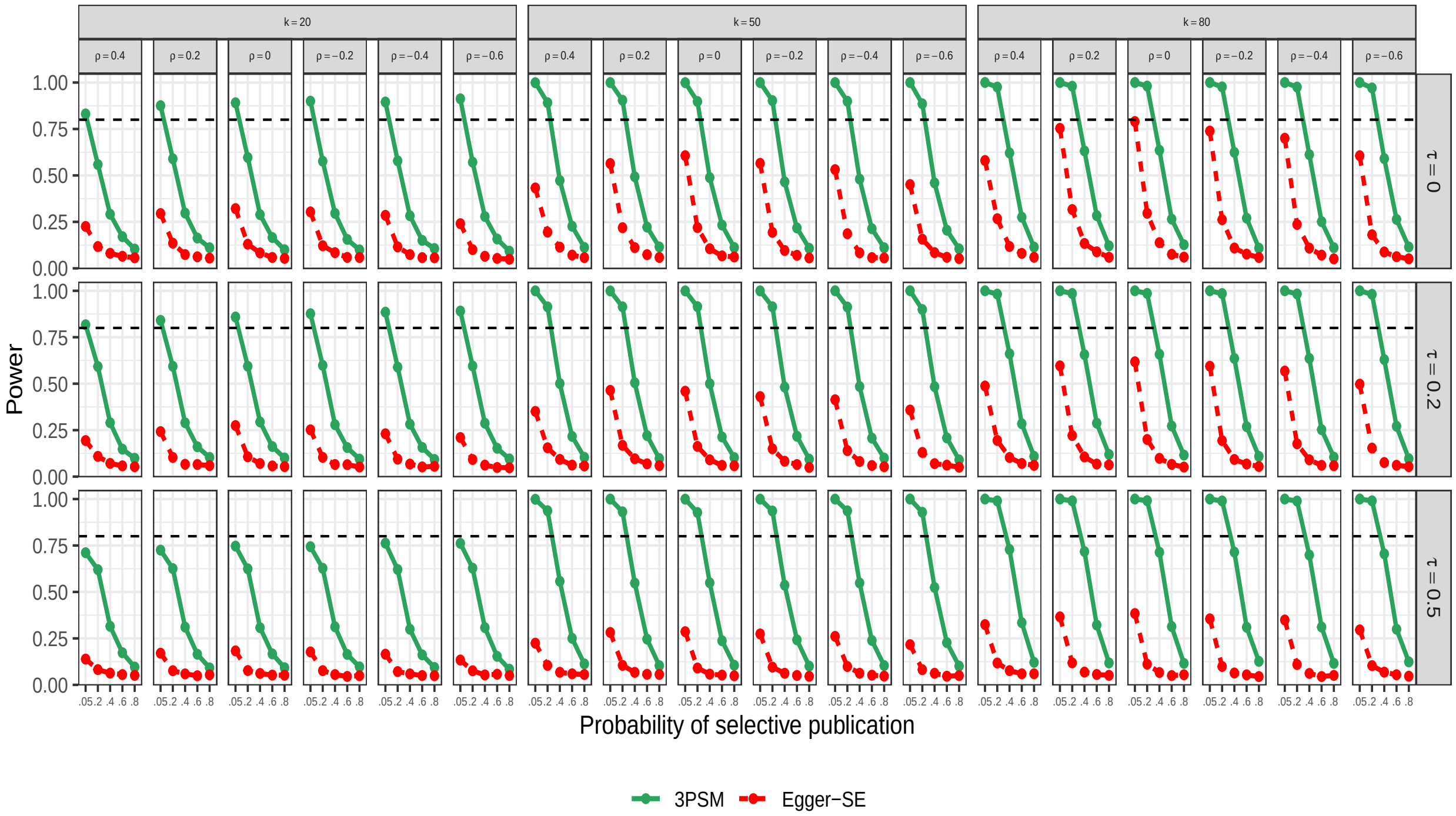

Figure S63: Power of Egger-SE and 3PSM modeling both covariates when $\beta_1 = 0$ and $\beta_2 = 0.5$.
